# Phosphinophosphoranes: Mixed-Valent Phosphorus Compounds with Ambiphilic Properties

**DOI:** 10.1021/acs.inorgchem.2c03166

**Published:** 2022-12-01

**Authors:** Natalia Szynkiewicz, Jarosław Chojnacki, Rafał Grubba

**Affiliations:** Department of Inorganic Chemistry, Faculty of Chemistry, Gdańsk University of Technology, 11/12 Gabriela Narutowicza Street, 80-233Gdańsk, Poland

## Abstract

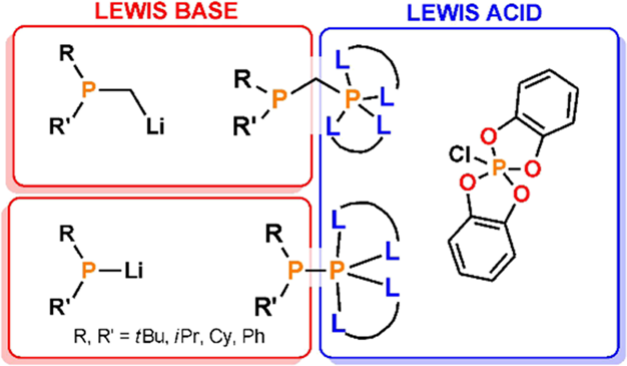

Herein, we present a simple synthesis of mixed-valent phosphinophosphoranes bearing three- and five-coordinate phosphorus centers. Compounds with phosphorus–phosphorus bonds were synthesized via a reaction of lithium phosphides RR′PLi with cat_2_PCl (cat = catecholate), whereas derivatives with methylene-linked phosphorus centers were obtained via a reaction of phosphanylmethanides RR′CH_2_Li with cat_2_PCl. The presence of accessible lone-pair electrons on the P-phosphanyl atom of phosphinophosphoranes during the reaction of the title compounds with H_3_B·SMe_2_, where phosphinophosphorane-borane adducts were formed quantitatively, was confirmed. Furthermore, the Lewis basic and Lewis acidic properties of the phosphinophosphoranes in reactions with phenyl isothiocyanate were tested. Depending on the structure of the starting phosphinophosphorane, phosphinophosphorylation of PhNCS or formation of a five-membered zwitterionic adduct was observed. The structures of the isolated compounds were unambiguously determined by heteronuclear nuclear magnetic resonance spectroscopy and single-crystal X-ray diffraction. Moreover, by applying density functional theory calculations, we compared the Lewis basicity and nucleophilicity of diversified trivalent P-centers.

## Abstract 2

The syntheses, structures, and reactivity of phosphinophosphoranes containing P−P or P−CH_2_−P structural motifs are presented.

## 1 Introduction

**Scheme 1:**
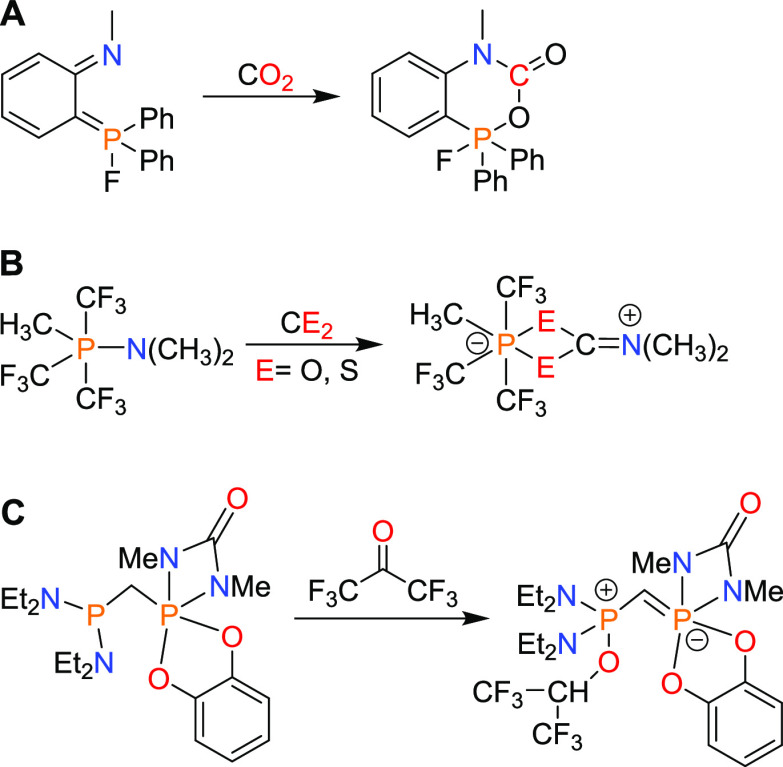
Examples of P(V)-Based FLPs Activating Small Molecules

In the field of metal-free catalysis, the main focus has been on frustrated Lewis pairs (FLPs) based on phosphorus and boron, which are considered the most effective and multipurpose agents for small-molecule activation.^1−4^ This includes ambiphilic compounds, which, despite the presence of a direct P–B bond, retain FLP-like reactivity.^5−9^ Although boron-based Lewis acids seem inseparable from the FLP concept, a study on potential other main-group Lewis acids has gained increasing interest.^10−12^ One promising research direction is the application of P(V)-based systems, which are readily acknowledged as Lewis acids; pentacoordinated phosphoranes with electron-withdrawing substituents are Lewis acidic because of their low-lying σ* orbitals.^13^ Although phosphorus compounds are generally employed as Lewis bases in FLPs, recent advances in phosphorus-based Lewis acid catalysis have proven that some phosphorus derivatives can also be applied as electron-deficient counterparts in these systems.^13−15^ One example of an active P(V)-based FLP is an N-base/P-acid species capable of irreversible CO_2_ fixation with ring-strained aminofluorophosphorane.^16^ Exposure to 1 atm of CO_2_ at ambient temperature leads to the insertion of CO_2_ into the P–N bond, generating a six-membered-ring product (Scheme 1A).

Another example of such reactivity is the insertion of CO_2_ and CS_2_ molecules into the polarized P–N bonds of aminophosphorane, leading to the formation of neutral six-coordinate carbamate and thiocarbamate derivatives (Scheme 1B).^17^ Herein, the directly bound nitrogen Lewis base and the phosphorus(V) Lewis acid activate the CE_2_ molecule with concomitant formation of an N–C bond and two P–E bonds, respectively. This type of reactivity resembles the insertion of CO_2_ and CS_2_ into P–P bonds^18^ and the insertion of CO_2_ into P–B bonds,^19^ as we have recently reported, and confirms that boron Lewis acids can be successfully replaced by phosphorus Lewis acids in the activation of small molecules.

Expectedly, an amine group acting as a Lewis basic counterpart may be successfully replaced by a phosphine moiety. In the literature, there have been only a few reports on the synthesis and reactivity of phosphinophosphoranes, including species with direct P–P^20−25^ bonds as well as methylene- and sulfur-bridged geminal phosphinophosphoranes.^22,26,27^ The presented results show that Lewis acidic and Lewis basic centers in ambiphilic P-systems can cooperate in reactions with small organic molecules, such as ketones, isocyanates, and azides, to yield phosphinophosphorylation products (Scheme 1C). Despite promising preliminary research on geminal phosphinophosphoranes, these systems seem to fade into oblivion, and their chemistry remains unexplored. Moreover, the reactivity of phosphinophosphoranes with direct P–P bonds has not been tested at all.

**Chart 1:**
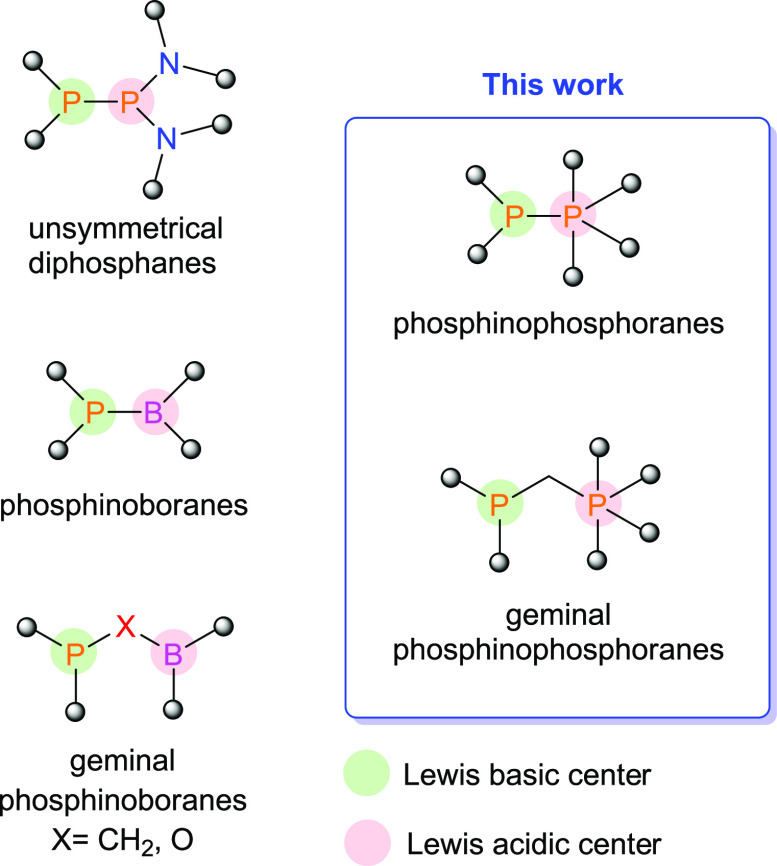
Comparison of Amphiphilic Phosphorus Compounds: Unsymmetrical Diphosphanes, Phosphinoboranes, and Phosphinophosphoranes

To develop active, metal-free systems that emulate the electronic environment of transition metals and consequently mimic their reactivity toward small molecules, we have turned to boron-free systems. Taking into account the advances in the field of FLP chemistry and the results of our research on the activation of P–P^18^ and P–B^19^ bond systems, we decided to study ambiphilic compounds based exclusively on phosphorus to obtain compounds that vary in the philicity of the P-center.^13,28^ In this study, we decided to design and synthesize two types of species that may act as small-molecule activators: phosphinophosphoranes and geminal phosphinophosphoranes (Chart 1).

Members of the first group—phosphinophosphoranes bearing P–P bonds—resemble unsymmetrical diphosphanes because of the presence of direct polarized P–P bonds and the various substituents attached to the P-centers, as well as phosphinoboranes due to the presence of Lewis acids and Lewis bases bound to each other. Both diphosphanes and phosphinoboranes have been found to insert small molecules into reactive P–P and P–B bonds via diphosphination and phosphinoboration reactions, respectively. Hence, we expected to observe analogous reactivity for mixed-valent phosphorus species, with the incorporation of a small molecule into a P–P bond via a phosphinophosphorylation reaction. Members of the second group, methylene-bridged geminal phosphinophosphoranes, resemble geminal FLPs in which the carbon or oxygen atom separates the Lewis acid and Lewis base.^6,29−31^

## 2 Results and Discussion

**Scheme 2:**
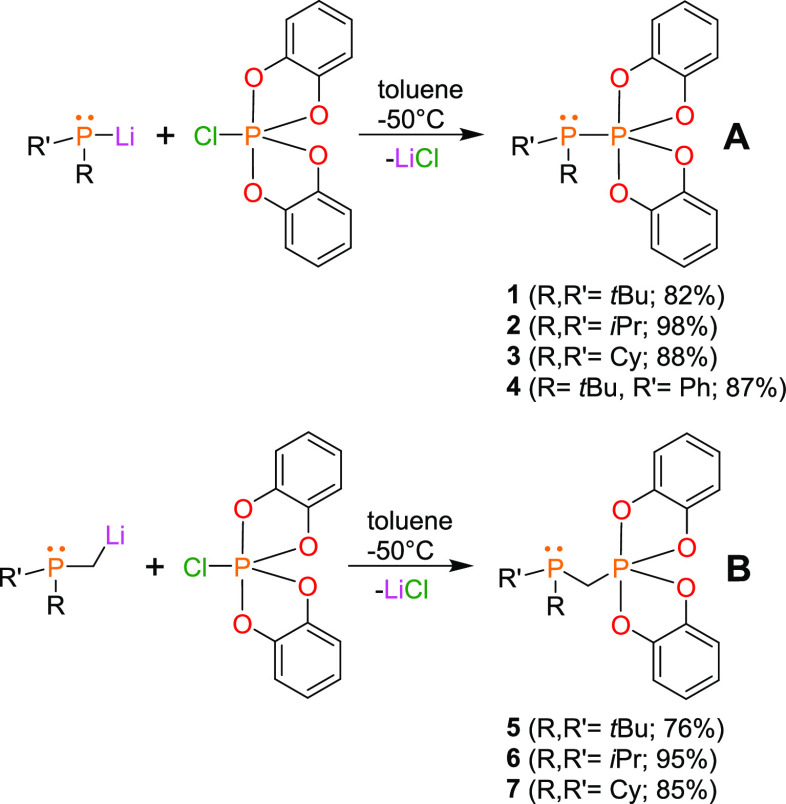
Syntheses of Phosphinophosphoranes **1**–**7**

To obtain mixed-valent diphosphorus species possessing a P–P bond, we utilized a simple metathesis reaction of lithium phosphide RR′PLi with chloro-substituted phosphorane cat_2_PCl (cat = catecholate) in toluene at −50 °C (Scheme 2A). Monitoring the progress of this synthesis by ^31^P NMR spectroscopy revealed that desired phosphinophosphoranes **1**–**4** were the major or only phosphorus-containing products. Therefore, analytically pure **1**–**4** were isolated by crystallization from the concentrated reaction mixture at low temperatures (**1**) or simply by evaporation of the solvent under high vacuum (**3**–**4**) as colorless crystals or white solids in high yields (82–98%). The ^31^P{^1^H} spectra of **1**–**4** display a set of two doublets attributed to the P1-phosphanyl (33.9–88.9 ppm) and P2-phosphoryl (−12.2 to 11.4 ppm) atoms. The large absolute values of the ^1^*J*_PP_ couplings in the range of 334–458 Hz confirm the presence of a P–P bond.

Geminal phosphinophosphoranes bearing methylene fragments between the phosphorus atoms were obtained using an analogous synthetic method. In this case, instead of RR′PLi, we used phosphanylmethanides RR′CH_2_Li as precursors of three-coordinate phosphorus fragments (Scheme 2B). The geminal phosphinophosphoranes **5**–**7** were formed quantitatively and isolated in the same manner as **1**–**4** in the form of white or yellowish solids in high yields (76–95%). The spectra of the geminal phosphinophosphoranes showed ^31^P{^1^H} resonances of the phosphanyl group in the range from −12.8 to 17.4 ppm, whereas signals of the phosphoryl fragment were observed at approximately 3 ppm. In comparison to those of **1**–**4**, the absolute values of phosphorus-phosphorus couplings in **5–7** were significantly smaller (73–87 Hz). Furthermore, the ^1^H and ^13^C NMR spectra of **5**–**7** showed characteristic doublets of doublets coupled with both phosphorus atoms assigned to protons and the carbon atom of the methylene bridge (see the ESI for details).

Notably, synthesis involving Ph_2_PLi, Ph*t*BuCH_2_Li, and Ph_2_PCH_2_Li also generated the expected phosphinophosphoranes of types **A** and **B**. However, the formation of other unidentified phosphorus compounds, likely the products of undesirable radical side reactions, was detected by ^31^P NMR spectroscopy. Therefore, we could not isolate the pure compounds of types **A** and **B** with Ph_2_P fragments and species of type **B** containing the *t*BuPh group.

Phosphinophosphoranes **1**–**7** were found to be stable under an inert atmosphere at room temperature. They were also shown to be prone to oxidation and hydrolysis, forming products with P=O and P–H functionalities.

**Figure 1:**
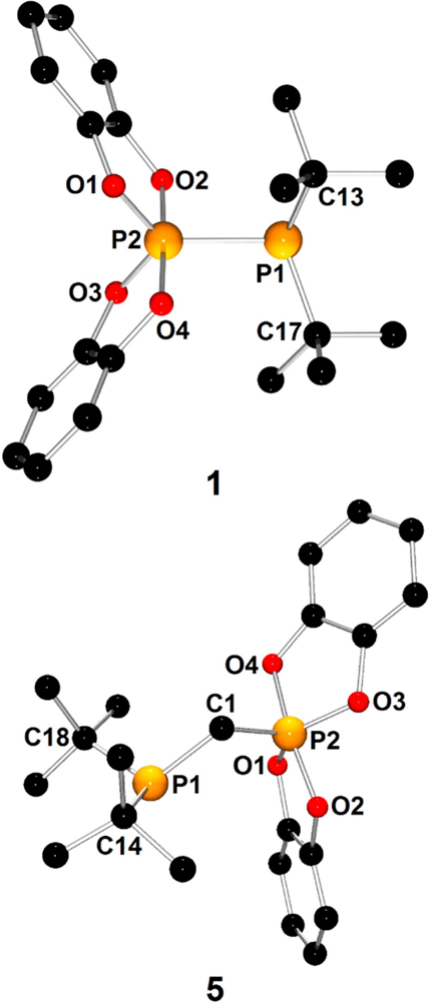
X-ray structures of **1** and **5** showing the atom-numbering scheme. H atoms are omitted for clarity. In the case of **5**, one of the two molecules present in the asymmetric unit was selected.

For all phosphinophosphoranes except **2**, X-ray-quality crystals were obtained, which allowed us to determine their molecular structures by single-crystal X-ray diffraction. The X-ray structures of **1** and **5** are presented in Figure 1, whereas the structures of the remaining phosphinophosphoranes are shown in Figures S5, S6, S11, and S13. The selected metric parameters of phosphinophosphoranes are collected in Table 1. The species belonging to groups **A** or **B** exhibited common structural features. Therefore, we discuss only representatives for each group in detail, namely, structures **1** and **5**. X-ray diffraction confirmed that phosphinophosphorane **1** (type **A**) contains mixed-valent phosphorus centers directly connected by chemical bonds: three-coordinate P1 atoms and pentacoordinate P2 atoms. The P1 atom of the phosphanyl group was found to have a pyramidal geometry with a sum of angles equal to 318.48°. The P2 atom, constituting the center of the phosphoryl group, exhibited a distorted trigonal bipyramidal geometry. The P2 atom shares a plane with the P1, O1, and O3 atoms, which occupy the equatorial positions of the bipyramid, whereas the remaining oxygen atoms O2 and O2 occupy the axial sites of the bipyramid. The P1–P2 distance of 2.236(1) Å was determined to be slightly longer than the expected distance for a single P–P bond (2.22 Å),^32^ consistent with the calculated P1–P2 Wiberg bond order of 0.875. The pyramidal geometry around the P1 atom and the long P1–P2 bond preclude the formation of significant π-interactions between the phosphorus centers and confirm the presence of accessible lone electron pair at the P1 atom. Furthermore, the axial phosphorus–oxygen bond distances were found to be in the range of 1.716(2)–1.735(2) Å and were significantly longer than their equatorial counterparts (1.638(2)–1.639(2) Å).

**Table 1:** Selected Bond Distances [Å] and Angles [°] for Phosphinophosphoranes

compound	**1**	**3**	**4**	**5**^a^	**6**^a^	**7**
P1–P2	2.236(1)	2.1902(6)	2.191(1)			
P1–CH_2_				1.862(6)	1.878(5)	1.885(5)
				1.867(7)	1.863(6)	
P2–CH_2_				1.795(6)	1.800(6)	1.794(5)
				1.803(6)	1.798(5)	
P2–O_eq_	1.639(2)	1.637(1)	1.640(3)	1.626(4)	1.644(4)	1.650(4)
	1.638(2)	1.639(1)	1.642(3)	1.641(4)	1.639(4)	1.642(4)
				1.558(9)	1.647(4)	
				1.631(5)	1.654(4)	
P2–O_ax_	1.735(2)	1.717(1)	1.718(3)	1.714(5)	1.712(4)	1.709(4)
	1.716(2)	1.723(1)	1.711(3)	1.713(4)	1.710(4)	1.695(4)
				1.750(8)	1.706(4)	
				1.710(4)	1.698(4)	
P1–P2–O_eq_	126.71(7)	110.95(3)	124.5(1)			
	119.88(7)	125.18(4)	115.9(1)			
P1–P2–O_ax_	86.88(7)	92.03(3)	88.87(9)			
	95.42(7)	95.67(3)	95.5(1)			
P1–CH_2_–P2				115.6(3)	112.1(3)	114.1(3)
				113.7(3)	113.7(3)	
H_2_C–P2–O_eq_				119.5(2)	112.8(2)	113.2(2)
				112.9(2)	115.8(2)	110.1(2)
				119.5(3)	113.2(2)	
				113.4(4)	111.8(2)	
H_2_C–P2–O_ax_				95.7(2)	94.2(2)	96.2(2)
				93.7(2)	96.7(2)	98.3(2)
				96.0(3)	95.4(2)	
				92.5(3)	97.9(2)	
O_eq_–P2–O_eq_	113.21(9)	123.86(5)	119.4(1)	127.6(2)	131.4(2)	136.7(2)
				127.0(4)	135.0(2)	
O_ax_–P2–O_eq_	90.35(9)	90.91(4)	91.1(1)	84.6(2)	84.5(2)	90.4(2)
	87.28(9)	84.61(4)	87.7(1)	90.7(2)	90.7(2)	84.5(2)
	91.51(9)	86.25(5)	86.2(1)	86.2(2)	90.9(2)	90.0(2)
	88.41(9)	90.82(5)	90.6(1)	90.3(2)	85.0(2)	84.4(2)
				91.4(4)	90.6(2)	
				89.8(3)	84.3(2)	
				80.3(3)	84.5(2)	
				91.1(2)	90.5(2)	

a For **5** and **6**, two molecules are present in the independent part of the unit cell; therefore, two sets of metric parameters are provided; O_eq_ – equatorial O atom; O_ax_ – axial O atom.

In the molecular structure of **5**, which is representative of geminal phosphinophosphoranes (type **B**), the geometries of the phosphanyl and phosphoryl groups resemble those observed for **1**. The most characteristic structural feature of **5** is the presence of a methylene group between P1 and P2. The C1–P1 and C1–P2 distances of 1.862(6) and 1.795(6) Å, respectively, were found to be close to typical single bond distances (1.86 Å).^32^ The P1–C1–P2 bond angle of 115.6(3)° is wider than the expected angle for sp^3^-hybridized carbon atoms, which can be explained by the steric hindrance of the substituents at P1 and P2.

**Figure 2:**
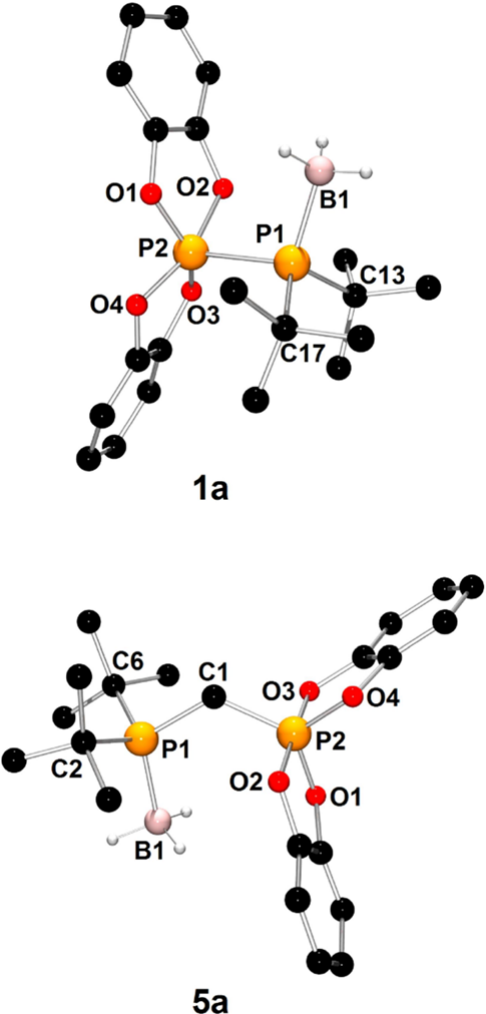
X-ray structures of **1a** and **5a** showing the atom-numbering scheme. H atoms are omitted for clarity, except those bonded to the boron atom.

**Scheme 3:**
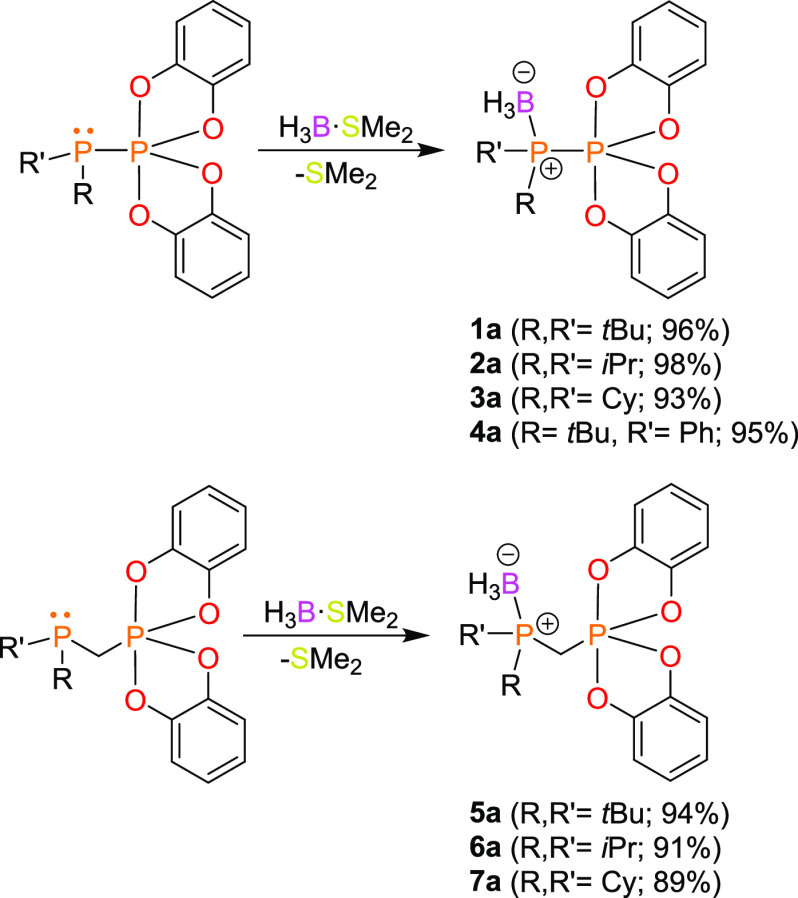
Syntheses of Phosphinophosphorane-Borane Adducts **1a**–**7a**

The Lewis basic properties of phosphinophosphoranes **1**–**7** were manifested in the reaction with H_3_B·SMe_2_ (Scheme 3). The formation of borane adducts **1a**–**7a** was confirmed collectively by NMR spectroscopy and X-ray diffraction. Monitoring the progress of these reactions by ^31^P and ^11^B NMR spectroscopy revealed the completed conversion of substrates within one hour. The reactions mentioned above proceeded very cleanly, and analytically pure borane adducts **1a**–7**a** were obtained in high yields (89–98%) by evaporation of the solvent and SMe_2_ under reduced pressure. Compared to that of the parent compounds **1**–**7**, the ^31^P{^1^H} NMR resonance of the P1 atom of the product compounds was significantly downfield shifted (except in **3a**), which corroborates the formation of a coordination bond between this atom and the boron atom of the BH_3_ moiety. On the other hand, the signal of the P2-phosphoryl atom was shifted only slightly upfield. Interestingly, for **1a**–**4a**, the absolute values of ^1^*J*_PP_ were significantly decreased compared to those of the parent phosphinophosphoranes and ranged from 22 to 106 Hz. A similar trend was observed for the adducts derived from geminal phosphinophosphoranes, where ^2^*J*_PP_ was not detectable (**5a**) or was lower than the values of the parent phosphorus substrates (**6a** and **7a**: 22 Hz). The ^11^B NMR spectra showed broad multiplets at approximately −40 ppm, consistent with the presence of a tetracoordinate boron center directly bonded to the phosphorus atom. The X-ray structures of representative adducts **1a** and **5a** are depicted in Figure 2. The X-ray diffraction analysis clearly shows the formation of a new P1–B1 bond as a result of the interaction of the lone electron pair of the phosphanyl group with the Lewis acidic boron atom. In comparison to parent compounds **1**–**7**, upon coordination of the BH_3_ molecule, the measured parameters of the phosphinophosphorane moiety in the structures of **1a**–**7a** were affected only slightly (Table 2).

**Table 2:** Selected Bond Distances [Å] and Angles [°] for Phosphinophosphorane-Borane Adducts

compound	**1a**	**2a**	**4a**	**5a**	**6a**^a^
P1–BH_3_	1.95(1)	1.937(1)	1.933(4)	1.935(2)	1.918(5)
					1.919(4)
P1–P2	2.266(9)	2.2051(5)	2.219(1)		
P1–CH_2_				1.851(2)	1.841(3)
					1.834(3)
P2–CH_2_				1.821(2)	1.809(3)
					1.806(3)
P2–O_eq_	1.610(7)	1.6283(8)	1.621(2)	1.627(1)	1.630(2)
	1.556(8)	1.6270(9)	1.628(2)	1.629(1)	1.619(2)
					1.619(2)
					1.624(2)
P2–O_ax_	1.801(8)	1.7094(9)	1.708(2)	1.713(1)	1.714(2)
	1.66(1)	1.7072(9)	1.701(2)	1.708(1)	1.707(2)
					1.723(2)
					1.703(2)
P1–P2–O_eq_	104.3(4)	122.07(3)	120.64(7)		
	111.0(4)	115.92(3)	120.52(7)		
P1–P2–O_ax_	91.6(3)	91.35(3)	91.53(7)		
	107.0(5)	91.13(3)	89.22(6)		
P1–CH_2_–P2				121.3(1)	116.8(2)
					117.0(2)
H_2_C–P2–O_eq_				130.65(8)	124.1(1)
				113.89(8)	114.8(1)
					115.2(1)
					123.2(1)
H_2_C–P2–O_ax_				88.91(7)	92.0(1)
				92.75(7)	91.5(1)
O_eq_–P2–O_eq_	143.5(5)	122.00(4)	118.82(9)	115.44(6)	121.1(1)
					121.6(1)
O_ax_–P2–O_eq_	87.1(4)	91.63(4)	88.73(9)	90.77(6)	90.5(1)
	85.0(5)	88.08(4)	91.64(9)	87.20(6)	88.3(1)
	83.3(4)	86.62(4)	87.59(9)	88.73(6)	85.9(1)
	93.0(5)	91.20(4)	91.28(9)	91.73(6)	91.6(1)
					86.3(1)
					91.3(1)
					87.7(1)
					91.4(1)

a For **6a**, two molecules are present in the independent part of the unit cell; therefore, two sets of metric parameters are provided; O_eq_—equatorial O atom; O_ax_—axial O atom.

**Figure 3:**
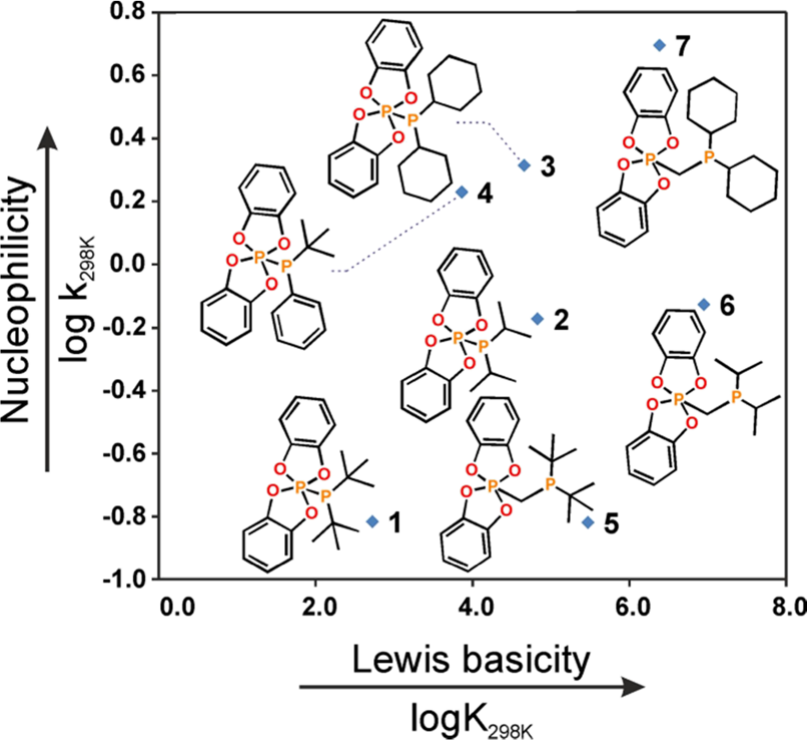
Comparison of the relative nucleophilicity and Lewis basicity of RR′**P** centers in phosphinophosphoranes and germinal phosphinophosphoranes.

Although the features of the Lewis basic counterpart are not the only factors determining the effective activation of small molecules by ambiphilic compounds, since we considered species with diversified RR′**P** centers, we focused solely on comparing trivalent P atoms. Hence, we investigated how the electronic and steric properties of the Lewis basic moiety influence their reactivity. To this end, we performed DFT calculations to elucidate the kinetics and thermodynamics of the reaction with the representative Lewis acid BH_3_. The values of the free energies Δ*G*^0^_298K_ and related equilibrium constants *K*_298K_ were consistent with the RR′**P** Lewis basicity, while the energy barriers Δ*G*^#^_298K_ were used to calculate rate constants *k*_298K_, providing information about the nucleophilicity of these centers (see the ESI Scheme S2, eq S1 and Table S24 for computational details).^33^ The analysis of the DFT results showed that species with separated P-atoms (PCH_2_P) were generally more Lewis basic than P–P bond-containing systems (with a maximum value for the *i*Pr-substituted **6**, Figure 3).

Since all the compounds except for **4** bear electron-donating substituents, the main factor contributing to the differences in nucleophilicity is steric congestion around the P-center (Figure 3). Indeed, Cy-substituted **3** and **7** were the most nucleophilic, while *t*Bu-substituted **1** and **5** were the least nucleophilic systems, indicating that the presence of a CH_2_ linker separating trivalent and pentavalent P atoms did not influence the nucleophilicity. Conversely, the basicity decreased once the electron density shifted toward Lewis acidic P atoms.

**Figure 4:**
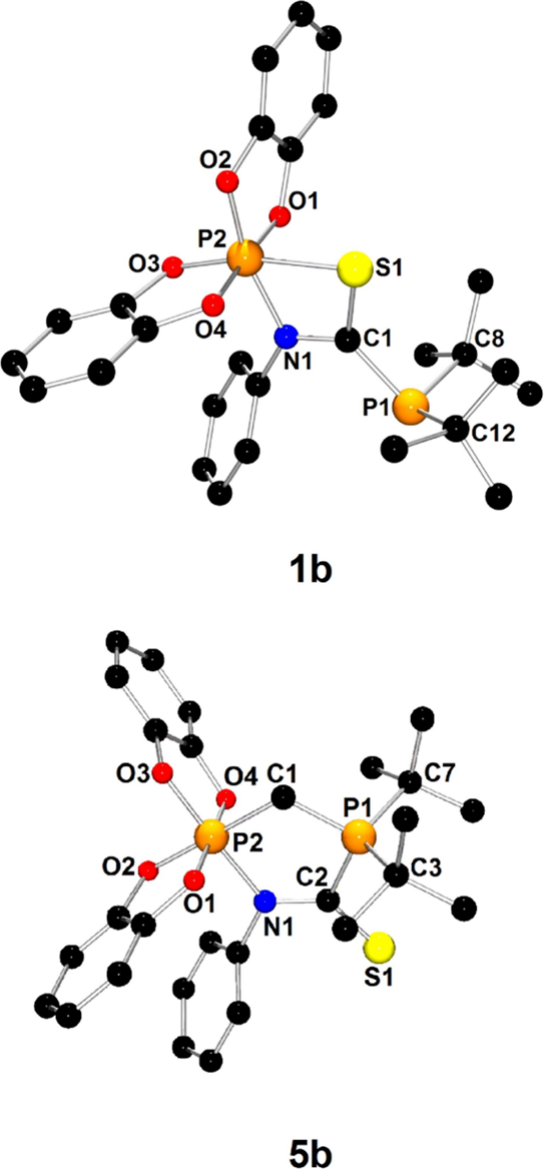
X-ray structures of **1b** and **5b** showing the atom-numbering scheme. H atoms are omitted for clarity.

**Scheme 4:**
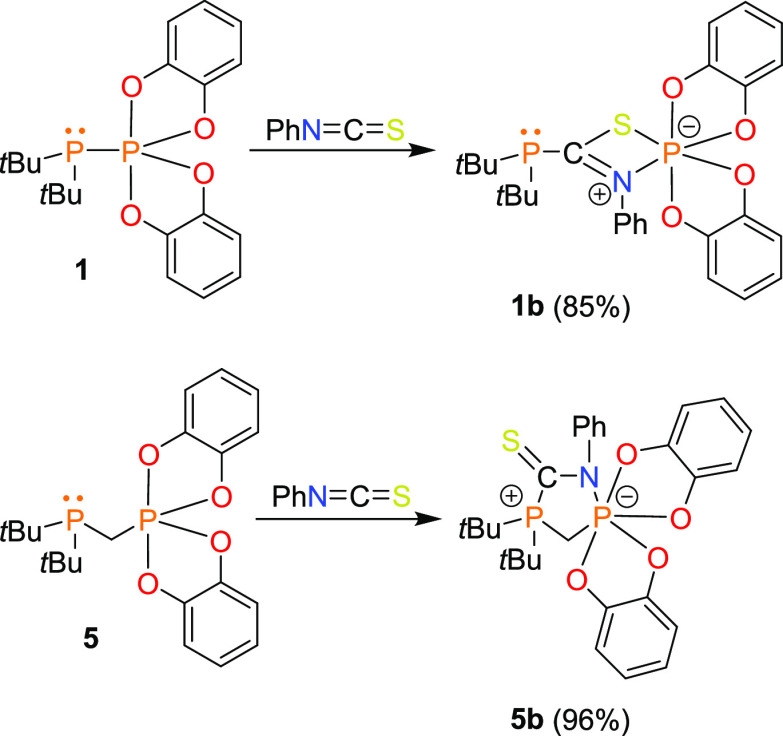
Reactions of Phosphinophosphoranes with PhNCS

Next, we tested the ambiphilic properties of phosphinophosphoranes in reactions with phenyl isothiocyanate. For these experiments, we selected representative compounds **1** and **5**. Phosphinophosphorane **1** reacted with PhNCS to generate phosphinophosphorylation product **1b** (Scheme 4). The ^31^P{^1^H} NMR spectrum of **1b** contained two singlets attributed to P1-phosphanyl P2-phosphoryl atoms, which were significantly upfield shifted in comparison to those of the starting phosphorus substrate (P1: 88.9 ppm for **1** vs 27.6 ppm for **1b**; P2: −12.2 ppm for 1 vs −83.6 ppm for **1b**). The X-ray diffraction analysis of **1b** confirmed the insertion of the PhNCS molecule into the P1–P2 bond with the formation of new C1–P1, N1–P2, and S1–P2 bonds (Figure 4). The phosphanyl group retained its pyramidal geometry (ΣP1 = 313,11°), whereas the geometry around the P2 atom became distorted octahedral because of the coordination of four oxygen atoms from two catecholate ligands and the nitrogen and sulfur atoms of the PhNCS moiety.

The structure of **1b** contains a planar four-membered ring composed of C1, N1, P2, and S1 atoms. The C1–N1 (1.318(3) Å) and C1–S1 (1.717 (3) Å) distances were found to be shorter than the expected distances for single covalent bonds (C–N: 1.46 Å; C–S: 1.78 Å),^32^ and the geometries around the C1 and N1 atoms were planar, indicating sp^2^ hybridization of these atoms and the presence of significant π-interactions between them. Indeed, natural bond orbital analysis (NBO) and calculated Wiberg bond orders for C1–N1 (1.48) and C1–S1 (1.28) supported this assumption. On the other hand, the N1–P2 and S1–P2 distances, with values of 1.875(2) and 2.2808(8) Å, respectively, were found to be longer than typical single covalent bonds (N–P: 1.82 Å; S–P: 2.14 Å).^32^

In contrast to the reaction involving **1**, geminal phosphinophosphorane **5** reacts with PhNCS, yielding zwitterionic adduct **5b** (Scheme 4). In the ^31^P{^1^H} NMR spectrum of **5b**, in comparison to that of **5**, the resonance of the P1 atom shifted more downfield, whereas the resonance of the P2 atom shifted significantly upfield (P1: 17.4 ppm for **5** vs 29.6 ppm for **5b**; P2: 2.7 ppm for **5** vs −91.2 ppm for **5b**), indicating substantial changes in the electron density at the P1 and P2 centers upon the addition of the PhNCS molecule. The X-ray structure analysis provided more insights into the structure of the **5b** adduct (Figure 4). As a result of electron pair donation from the Lewis basic P1 atom to the electron-deficient C1 atom of the PhNCS molecule and acceptance of the electron pair of the N1 atom of the PhNCS molecule by the Lewis acidic P2 atom, a five-membered ring formed. Furthermore, these donor–acceptor interactions led to changes in the geometries around the phosphorus centers, where the P1 and P2 atoms adopted distorted tetrahedral and octahedral geometries, respectively. Similar to the structure of **1b**, the geometries around the C1 and N1 atoms were almost planar, and the C1–N1 bond exhibited a partial double bond character, as confirmed by the relatively short C1–N1 bond distance of 1.329(2) Å and the calculated Wiberg bond order of 1.37. Otherwise, the N1–P2 bond distance of 1.910(1) Å substantially exceeded the expected bond length for a single covalent N–P bond of 1.82 Å.^32^ In contrast to the structure of **1b**, in the case of **5b**, the S1 atom was not directly connected to the P2 atom but formed a terminal double bond with the C1 atom. DFT calculations confirmed that this substitution pattern, resulting from the formation of P–C and P–N bonds, is more thermodynamically privileged than the P–C and P–S bond isomer (see Scheme S1 in the ESI for details).

**Figure 5:**
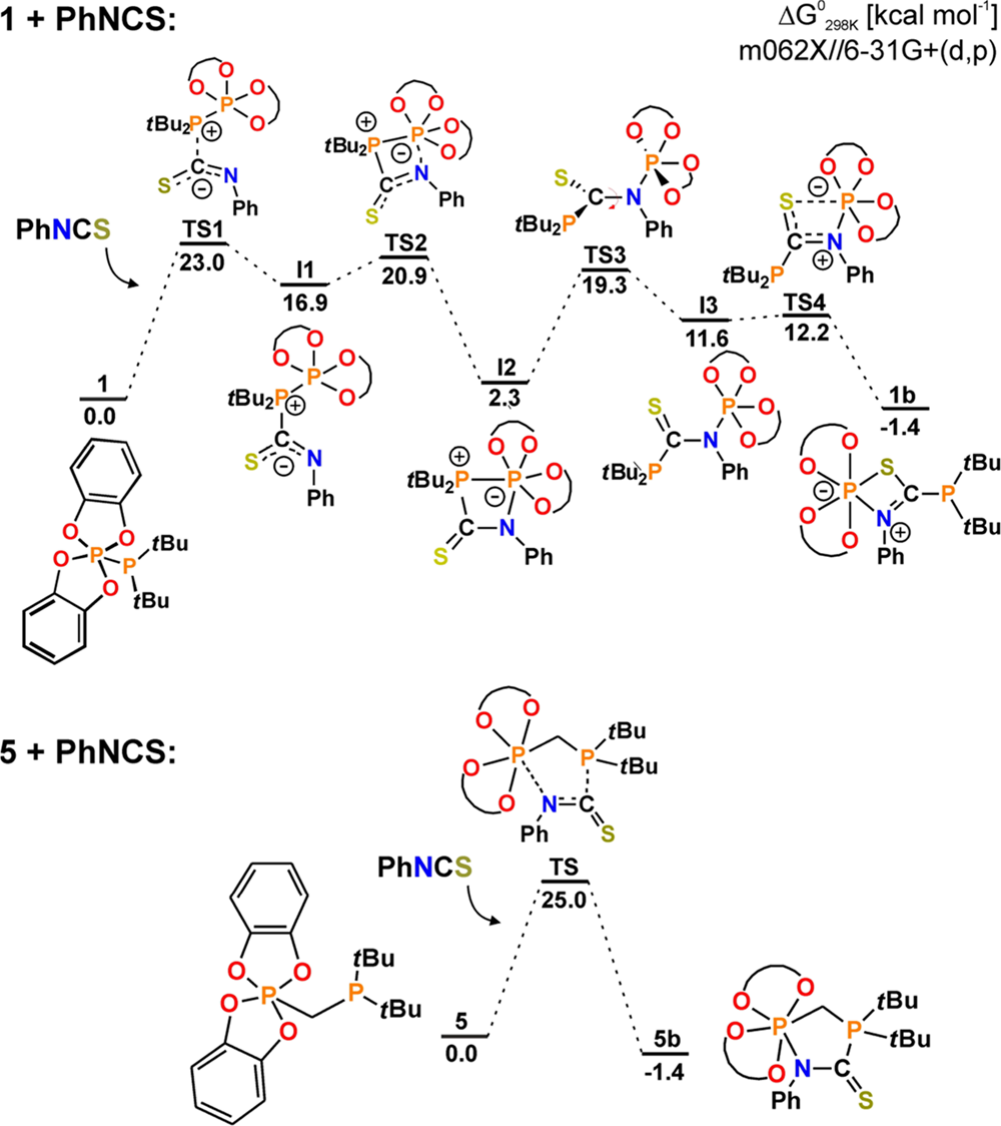
Mechanism of the PhNCS activation by **1** and **5** calculated at the M06-2X//6-31 + G(d,p) level of theory.

In the reaction of PhNCS with diphosphanes bearing polarized P–P bonds, one of the trivalent P-centers acts as soft acidic center binding with a soft base, S atom.^34^ Conversely, the pentavalent cat_2_**P** atom is a typical Lewis acid; hence, it reacts preferably with the hard basic center in PhNCS, the N atom (Figure 5). In the case of **5**, the reaction proceeds via a single transition state involving the simultaneous formation of P–C and P–N bonds to give the final product **5b**. Activation of PhNCS by **1** is a multi-step process that results in the formation of both P–N and P–S bonds. The reaction starts with a nucleophilic attack of **P***t*Bu_2_ on the C atom followed by binding of the P(V) center to the nitrogen atom to yield intermediate four-membered-cycle **I2**. By rotation about the C–N bond, the P–P bond is cleaved and replaced by S···P interactions (**I3**), consequently giving the final heterocyclic product **1b**.

## 3 Conclusions

The described synthetic methods offer simple synthetic access to two types of mixed-valence phosphorus compounds – phosphinophosphoranes containing P–P or P–CH_2_–P structural motifs. Our reactivity study revealed that these species have ambiphilic properties, with three-coordinate P-phosphanyl atoms and five-coordinate P-phosphoryl atoms acting as Lewis basic and Lewis acidic sites, respectively. The proximity of the reactive P-centers enhances their reactivity and supports the activation of small molecules. As shown in the reactions involving PhNCS, two reactivity patterns can be distinguished depending on the structure of phosphinophosphoranes: phosphinophosphorylation (insertion of a small molecule into the P–P bond) or the formation of cyclic five-membered zwitterionic products. The products generated by the insertion of a small molecule into the P–P bond may be easily applied as ligands in organometallic chemistry or may serve as substrates for the synthesis of more complex compounds that are difficult or impossible to obtain by other means. Both types of phosphinophosphoranes offer an unprecedented approach in the field of metal-free catalysis that may trigger further developments in the facile fixation and functionalization of organic and inorganic molecules. Theoretical investigations of Lewis basicity and nucleophilicity of diversified trivalent P-centers revealed that increased basicity is associated with the presence of a CH_2_ linker precluding delocalization of electron density toward a Lewis acidic cat_2_P center. Conversely, the key factor contributing to the nucleophilicity is the decreased bulkiness of substituents bound to the RR′**P** center.

## Supporting Information Available

Crystallographic details; NMR spectroscopic details; and computational details (PDF)
