# Supplementary material for: Phosphinophosphoranes: Mixed-Valent Phosphorus Compounds with Ambiphilic Properties
Source: Inorg Chem. 2022 Dec 1;61(49):19925–32. doi: 10.1021/acs.inorgchem.2c03166 (PMC9749023; doi:10.1021/acs.inorgchem.2c03166)
Supplement: Supplementary file 1 — ic2c03166_si_001.pdf [file ic2c03166_si_001.pdf]

Supplementary Material for

Phosphinophosphoranes: Mixed-valent phosphorus compounds with ambiphilic properties

Natalia Szynkiewicz, Jarosław Chojnacki**

*and Rafał Grubba*ǂ*

ǂ*Corresponding author, e-mail: rafal.grubba@pg.edu.pl*

*Department of Inorganic Chemistry, Faculty of Chemistry,

Gdansk University of Technology, G. Narutowicza St. 11/12. PL-80-233, Gdansk, Poland.

CONTENTS:

Experimental section 4

Preparation of 1 4

Preparation of 1a 5

Preparation of 1b 6

Preparation of 2 7

Preparation of 2a 8

Preparation of 3 9

Preparation of 3a 10

Preparation of 4 10

Preparation of 4a 11

Preparation of 5 12

Preparation of 5a 13

Preparation of 5b 14

Preparation of 6 15

Preparation of 6a 16

Preparation of 7 17

Preparation of 7a 18

X-ray structures analysis 20

General methods 20

Specific details for individual structures 20

Single crystal X-ray structure analysis of 1 26

Single crystal X-ray structure analysis of 1a 27

Single crystal X-ray structure analysis of 1b 28

Single crystal X-ray structure analysis of 2a 29

Single crystal X-ray structure analysis of 3 30

Single crystal X-ray structure analysis of 4 31

Single crystal X-ray structure analysis of 4a 32

Single crystal X-ray structure analysis of 5 33

Single crystal X-ray structure analysis of 5a 34

Single crystal X-ray structure analysis of 5b 35

Single crystal X-ray structure analysis of 6 36

Single crystal X-ray structure analysis of 6a 37

Single crystal X-ray structure analysis of 7 38

Spectroscopic data 39

NMR spectra of isolated compounds....39
NMR spectra of 1....39
NMR spectra of 1a....40
NMR spectra of 1b....42
NMR spectra of 2....44
NMR spectra of 2a....45
NMR spectra of 3....47
NMR spectra of 3a....49
NMR spectra of 4....51
NMR spectra of 4a....52
NMR spectra of 5....54
NMR spectra of 5a....56
NMR spectra of 5b....58
NMR spectra of 6....59
NMR spectra of 6a....61
NMR spectra of 7....63
NMR spectra of 7a....64
IR spectra of isolated compounds....67
DFT calculations....75
General methods....75
Values of free energy of formation of considered products....76
Optimized structures and Cartesian coordinates....78
References....79

Experimental section

All manipulations were carried out under a dry argon atmosphere using flame-dried Schlenk-type glassware on a vacuum line or in a glove-box. Solvents were dried by standard procedures over Na(K)/K/Na/benzophenone and distilled under argon. 1D (^{31}P, ^{13}C, ^{11}B and ^{1}H) and 2D NMR spectra in C_6D_6 or toluene-d_8 solution were recorded on a Bruker AV400 MHz spectrometer (external standard TMS for ^{1}H and ^{13}C; 85% H_3PO_4 for ^{31}P) at an ambient or lower temperature. Reaction progress was monitored by $^{31}P\{^{1}H\}$ and ^{31}P spectra of reaction mixtures. The FTIR spectra of crystalline products were recorded using a Nicolet iS50 FT-IR spectrometer equipped with the Specac Quest single-reflection diamond attenuated total reflectance (ATR) accessory. Spectral analysis was carried out by using the OMNIC software package. Elemental analyses were performed using a Elementar's Vario El Cube CHNS micro elemental analyzer. The basic principle of quantitative CHNS analysis is the high-temperature, oxidative combustion of samples. The gaseous products of combustion are purified, separated in absorption columns into individual components (nitrogen, carbon dioxide, sulphur dioxide, water vapour) and detected in the measuring cell of a TCD detector (thermal conductivity detector). **R_2CH_2PLi** (R = *i*Pr, Cy, *t*Bu) were synthesized *via* the procedure described in [1] and **cat_2PCl** was obtained as described in [2].

Preparation of 1

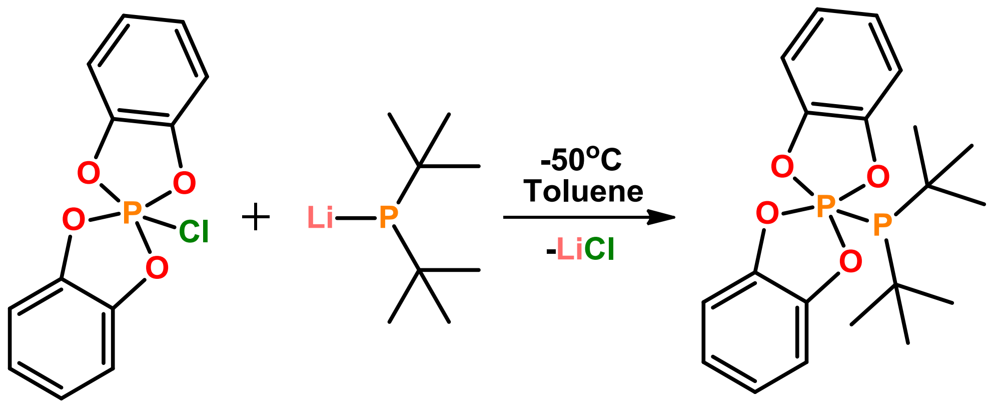

1

To a suspension of $t\mathrm{Bu_2PLi}$ (0.152 g, 1.00 mmol) in 15 cm^3 of toluene cooled to -50°C, a suspension of cat_2PCl (0.283 g, 1.00 mmol) in 5 cm^3 of toluene was added dropwise. The reaction mixture was stirred at -50°C for 3 hours and then allowed to warm to room temperature and kept stirring for an hour. The reaction mixture was filtered and the solution was concentrated and left at -30°C to afford X-ray quality crystals of **1**. The crystalline product was separated and dried under vacuum (0.01 Torr), giving **1** as analytically pure white solid. Yield 82% (0.322 g, 0.821 mmol).

NMR:

$^{31}P\{^{1}H\}$ NMR (C_6D_6, 298K): δ 88.9 (d, $^{1}J_{PP}$ = 457.8 Hz, P*t*Bu$_2$, 12.2 (d, $^{1}J_{PP}$ = 457.8 Hz, Pcat$_2$).

^{1}H NMR (C_6D_6, 298K): δ 6.82 (m, 4H, *m*-C*H*), 6.60 (m, 4H, *p*-C*H*), 1.30 (dd, 18H, $^{3}J_{PH}$ = 11.9 Hz, $^{4}J_{PH}$ = 2.0 Hz, C*H*$_3$).

$^{13}C\{^1H\}$ NMR (C_6D_6, 298K): δ 144.8 (s, *ortho*-C), 121.9 (s, *para*-CH), 111.0 (d, $^3J_{PC}$ = 10.3 Hz, *meta*-CH), 35.4 (dd, $^1J_{PC}$ = 35.9 Hz, $^2J_{PC}$ = 6.6 Hz, *C*(CH_3)$_3$), 30.7 (dd, $^2J_{PC}$ = 14.7 Hz, $^3J_{PC}$ = 12.5 Hz, C(*CH$_3$*)$_3$).

Elemental analysis: calcd. for $C_{20}H_{26}O_4P_2$: C, 61.22; H, 6.68. Found: C, 61.13; H, 6.628.

IR (solid): $\tilde{\nu}$ = 2978, 2954, 2892, 2860, 1478, 1359, 1340, 1254, 1239, 1195, 1171, 1104, 1097, 1010, 888, 840, 783, 766, 736, 682, 655, 626, 552, 515, 455, 441.

Preparation of 1a

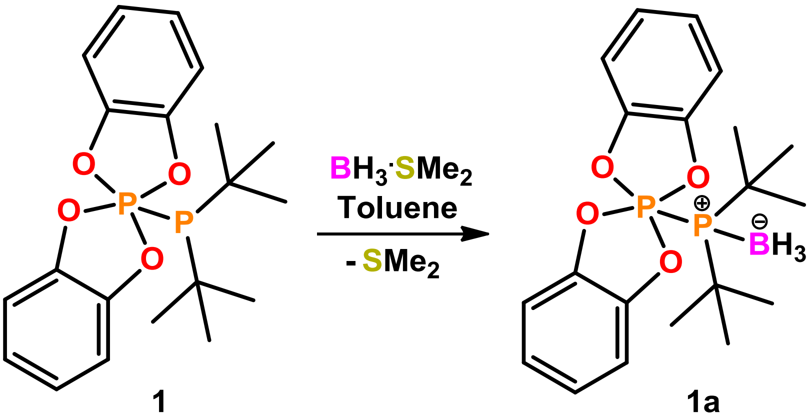

To a solution of **1** (98 mg, 0.250 mmol) in 5 cm^3 of toluene $BH_3{\cdot}SMe_2$ (0.025 cm^3, 19 mg, 0.25 mmol) was added at room temperature. The solution was stirred for an hour. $^{31}P\{^1H\}$ NMR of the reaction mixture revealed the complete conversion of **1** into **1a**. The solvent was evaporated, and the residue was dried under a vacuum (0.01 Torr) at 50°C for 2 hours to remove all volatiles. The oily product slowly solidifies at -20°C to afford X-ray quality crystals of **1a**. Yield 96% (97 mg, 0.239 mmol).

NMR:

$^{31}P\{^1H\}$ NMR (C_6D_6, 298K): δ 106.7 (broad m, P*t*Bu$_2$), -2.2 (d, $^1J_{PP}$ = 29.1 Hz, Pcat$_2$).

^{11}B (C_6D_6, 298K): -37.5 (broad m).

^1H NMR (C_6D_6, 298K): δ 6.79 (m, 4H, *m*-C*H*), 6.60 (m, 4H, *p*-C*H*), 1.37 (overlapped broad m, 3H, B*H$_3$*), 1.35 (d, 9H, $^3J_{PH}$ = 13.6 Hz, C*H$_3$*), 1.34 (d, 9H, $^3J_{PH}$ = 13.6 Hz, C*H$_3$*).

$^{13}C\{^1H\}$ NMR (C_6D_6, 298K): δ 144.3 (s, *ortho*-C), 122.3 (s, *para*-CH), 111.0 (d, $^3J_{PC}$ = 11.8 Hz, *meta*-CH), 36.8 (d, $^1J_{PC}$ = 10.0 Hz, *C*(CH_3)$_3$), 36.8 (d, $^1J_{PC}$ = 6.3 Hz, *C*(CH_3)$_3$), 29.2 (d, $^2J_{PC}$ = 5.4 Hz, C(*CH$_3$*)$_3$), 29.1 (d, $^2J_{PC}$ = 5.4 Hz, C(*CH$_3$*)$_3$).

Elemental analysis: calcd. for $C_{20}H_{29}BO_4P_2$: C, 59.14; H, 7.20. Found: C, 58.94; H, 7.088.

IR (solid): $\tilde{\nu}$ = 2969, 2930, 2902, 2870, 2404 (**B-H**), 1480, 1392, 1368, 1351, 1261, 1239, 1205, 1173, 1152, 1103, 1069, 1009, 906, 859, 793, 741, 718, 646, 625, 587, 538, 521, 456.

Preparation of 1b

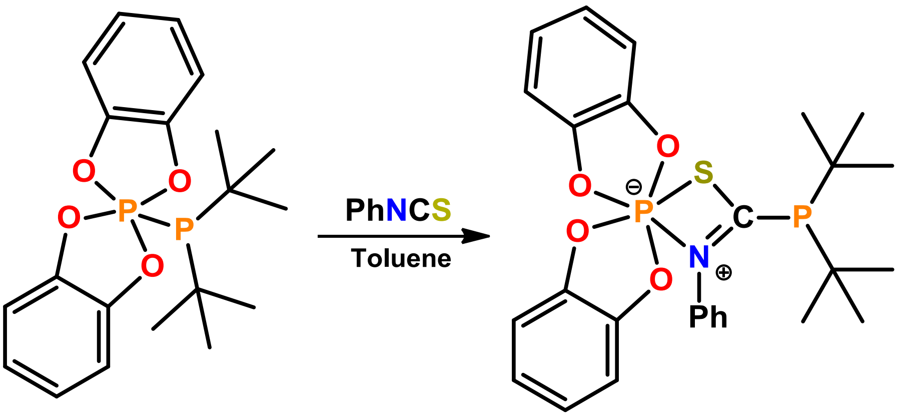

To a solution of **1** (196 mg, 0.5 mmol) in 5 cm^3 of toluene, an excess of PhNCS (0.108 cm^3, 120 mg, 1.0 mmol) was added at room temperature. The solution was stirred for 10 days. $^{31}P\{^1H\}$ NMR of the reaction mixture revealed the complete conversion of **1** into **1b**. The solvent was evaporated, and the residue was dried under a vacuum (0.01 Torr) at 50°C for an hour to remove all volatiles. Then it was washed with pentane and dried again to finally afford **1b** as yellow solid (224 mg, 0.425 mmol) in 85% yield. X-ray quality crystals were grown from dichloromethane solution layered with pentane at 4°C.

NMR:

$^{31}P\{^1H\}$ NMR ($CDCl_3$, 298K): δ 27.6 (s, P*t*Bu_2), -83.6 (s, $Pcat_2$).

^1H NMR ($CDCl_3$, 298K): δ 7.19 – 6.30 (overlapped broad m, 13H, *o,m,p*-C*H* of NPh and $Pcat_2$), 1.20 (broad m, 18H, C*H_3*).

$^{13}C\{^1H\}$ NMR ($CDCl_3$, 298K): δ 204.3 (dd, $^1J_{PC}$ = 71.8 Hz, $^2J_{PC}$ = 8.2 Hz, N*C*S), 147.5 (broad m, *o*-C, $Pcat_2$), 146.5 (broad m, *o*-C, $Pcat_2$), 143.6 (broad m, *o*-C, $Pcat_2$), 142.0 (d, $^2J_{PC}$ = 5.4 Hz, $^3J_{PC}$ = 4.5 Hz, *ipso*-C, NPh), 128.6 (d, $^3J_{PC}$ = 2.7 Hz, *o*-CH, NPh), 128.4 (s, *m*-CH, NPh), 126.9 (s, *p*-CH, NPh), 121.0 (broad s, *p*-CH, $Pcat_2$), 111.7 (broad m, *m*-CH, $Pcat_2$), 110.5 (broad m, *m*-CH, $Pcat_2$), 109.7 (broad m, *m*-CH, $Pcat_2$), 34.3 (braod m *C*(CH_3)$_3$), 30.0 (d, $^2J_{PC}$ = 14.5 Hz, *C*(CH_3)$_3$).

Elemental analysis: calcd. for $C_{27}H_{31}NO_4P_2S$: C, 61.47; H, 5.92; N, 2.66; S, 6.08. Found: , 61.25; H, 5.905; N, 2.61; S, 5.982.

IR (solid): $\tilde{\upsilon}$ = 3198, 3132, 3050, 2974, 1604, 1487 (**C=N**), 1387, 1371, 1245, 1206, 1178, 1101, 1014, 909, 893, 875, 820, 726, 642, 542, 488, 472.

Preparation of 2

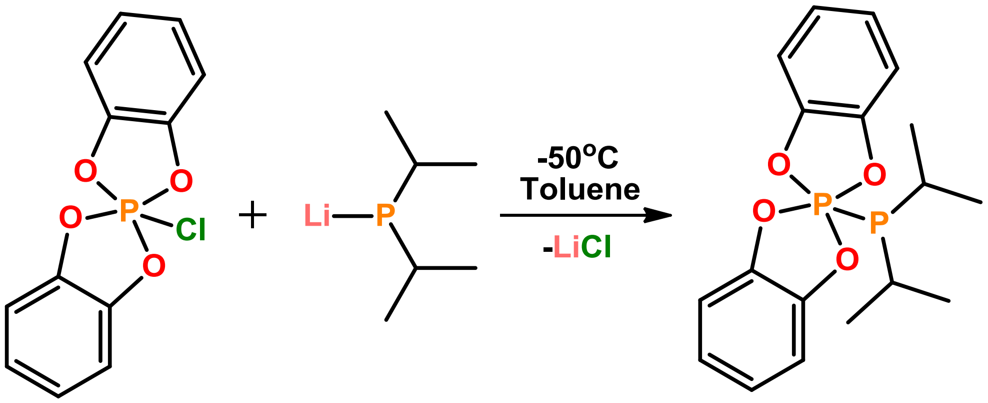

2

To a suspension of *i*Pr_2PLi (0.124 g, 1.00 mmol) in 15 cm^3 of toluene cooled to -50°C, a suspension of cat_2PCl (0.283 g, 1.00 mmol) in 5 cm^3 of toluene was added dropwise. The reaction mixture was stirred at -50°C for 2 hours and then allowed to warm to room temperature and kept stirring for an hour. The solvent was evaporated, and the residue was dried under a vacuum (0.01 mmHg) for 30 minutes at 50°C to remove all volatiles. The crude product was dissolved in 10 cm^3 of petroleum ether and filtered. Removal of the solvent under vacuum afforded 0.358 g (0.983 mmol) of **2** as a yellowish powder in 98% yield.

NMR:

$^{31}P\{^1H\}$ NMR (C_6D_6, 298K): δ 41.4 (d, $^1J_{PP}$ = 377.8 Hz, P*i*Pr_2), 10.9 (d, $^1J_{PP}$ = 377.8 Hz, $Pcat_2$).

^1H NMR (C_6D_6, 298K): δ 6.81 (m, 4H, *m*-C*H*), 6.60 (m, 4H, *p*-C*H*), 2.37 (m, 1H, C*H*CH_3), 2.31 (m, 1H, C*H*CH_3), 1.16 (broad m, 6H, CH_3), 1.06 (broad m, 6H, CH_3).

$^{13}C\{^1H\}$ NMR (C_6D_6, 298K): δ 145.0 (s, *ortho*-C), 121.9 (s, *para*-CH), 110.9 (d, $^3J_{PC}$ = 10.0 Hz, *meta*-CH), 24.2 (d, $^1J_{PC}$ = 21.8 Hz, *CH*CH_3), 24.1 (d, $^1J_{PC}$ = 20.9 Hz, *CH*CH_3), 22.0 (broad d, $^2J_{PC}$ = 13.6 Hz, CH_3), 21.9 (broad d, $^2J_{PC}$ = 13.6 Hz, CH_3), 20.6 (broad d, $^2J_{PC}$ = 10.9 Hz, CH_3), 20.4 (broad d, $^2J_{PC}$ = 10.9 Hz, CH_3).

Elemental analysis: calcd. for $C_{18}H_{22}O_4P_2$: C, 59.34; H, 6.09. Found: C, 59.10; H, 6.087.

IR (solid): $\tilde{\upsilon}$ = 3069, 2961, 2926, 2867, 1619, 1479, 1341, 1277, 1238, 1199, 1179, 1151, 1100, 1008, 952, 920, 843, 787, 764, 733, 685, 549, 520, 459, 444.

Preparation of 2a

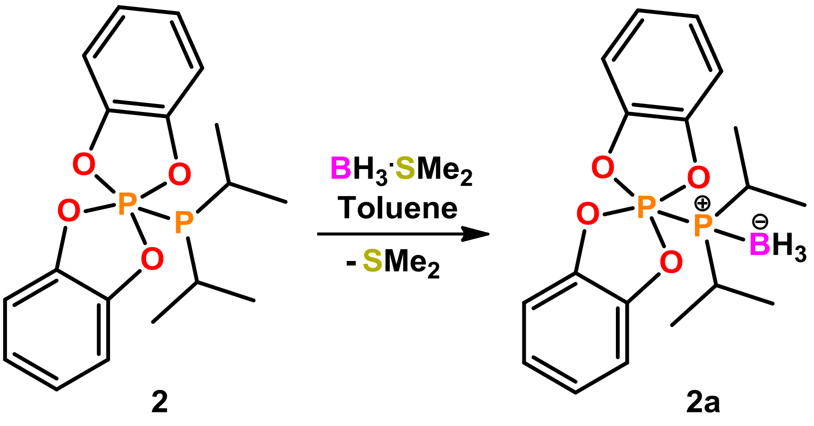

To a solution of **2** (91 mg, 0.250 mmol) in 5 cm^3 of toluene $BH_3{\cdot}SMe_2$ (0.025 cm^3, 19 mg, 0.25 mmol) was added at room temperature. The solution was stirred for an hour. $^{31}P\{^1H\}$ NMR of the reaction mixture revealed the complete conversion of **2** into **2a**. The solvent was evaporated, and the residue was dried under a vacuum (0.01 Torr) at 50°C for 2 hours to remove all volatiles. The oily product slowly solidifies at -20°C to afford X-ray quality crystals of **2a**. Yield 98% (93 mg, 0.241 mmol).

NMR:

$^{31}P\{^1H\}$ NMR (C_6D_6, 298K): δ 72.2 (broad m, P*i*Pr$_2$), -4.4 (d, $^1J_{PP}$ = 58.1 Hz, Pcat$_2$).

^{11}B (C_6D_6, 298K): -40.7 (broad m).

^1H NMR (C_6D_6, 298K): δ 6.77 (m, 4H, *m*-C*H*), 6.59 (m, 4H, *p*-C*H*), 2.21 (overlapped m, 2H, C*H*CH$_3$), 1.32 (overlapped broad m, 3H, B*H$_3$*), 1.15 (dd, $^3J_{HH}$ = 7.0 Hz, $^3J_{PH}$ = 15.2 Hz, 6H, C*H$_3$*), 1.07 (ddd, $^3J_{HH}$ = 7.1 Hz, $^3J_{PH}$ = 15.1 Hz, $^4J_{PH}$ = 1.2 Hz, 6H, C*H$_3$*).

$^{13}C\{^1H\}$ NMR (C_6D_6, 298K): δ 144.5 (s, *ortho*-C), 122.3 (s, *para*-CH), 110.9 (d, $^3J_{PC}$ = 11.7 Hz, *meta*-CH), 24.7 (d, $^1J_{PC}$ = 20.5 Hz, *CH*CH$_3$), 24.6 (d, $^1J_{PC}$ = 20.5 Hz, *CH*CH$_3$), 18.0 (d, $^2J_{PC}$ = 5.9 Hz, CH$_3$), 17.7 (d, $^2J_{PC}$ = 5.1 Hz, CH$_3$).

Elemental analysis: calcd. for $C_{18}H_{25}BO_4P_2$: C, 57.17; H, 6.66. Found: C, 57.09; H, 6.532.

IR (solid): $\tilde{\upsilon}$ = 3064, 2971, 2938, 2875, 2389 (**B-H**), 2377 (**B-H**), 2343 (**B-H**), 1624, 1597, 1479, 1339, 1236, 1193, 1103, 1060, 1032, 1010, 926, 905, 869, 789, 760, 743, 706, 621, 537, 518, 470, 452.

Preparation of 3

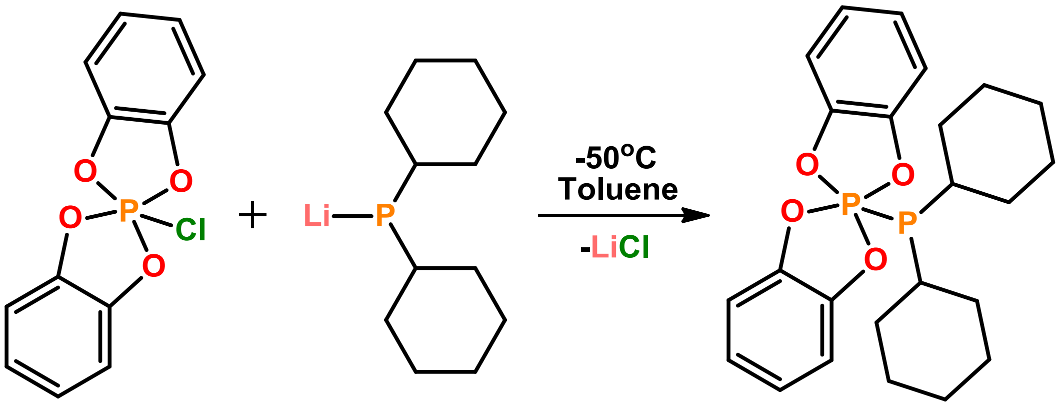

3

To a suspension of Cy_2PLi (0.204 g, 1.00 mmol) in 15 cm^3 of toluene cooled to -50°C, a suspension of cat_2PCl (0.283 g, 1.00 mmol) in 5 cm^3 of toluene was added dropwise. The reaction mixture was stirred at -50°C for 2 hours and then allowed to warm to room temperature and kept stirring for an hour. The solvent was evaporated, and the residue was dried under a vacuum (0.01 mmHg) for 30 minutes at 50°C to remove all volatiles. The crude product was dissolved in 10 cm^3 of petroleum ether and filtered. Removal of the solvent under vacuum afforded 0.392 g (0.882 mmol) of **3** as a white powder in 88% yield. X-ray quality crystals were grown from toluene at -20°C.

NMR:

$^{31}P\{^1H\}$ NMR (C_6D_6, 298K): δ 33.9 (d, $^1J_{PP}$ = 377.9 Hz, PCy_2), 11.4 (d, $^1J_{PP}$ = 377.9 Hz, $Pcat_2$).

^1H NMR (C_6D_6, 298K): δ 6.84 (m, 4H, *m*-C*H*), 6.62 (m, 4H, *p*-C*H*), 2.24 (m, 2H, C*H*CH_2), 2.00 – 1.06 (broad overlapped m, 20H, CH_2).

$^{13}C\{^1H\}$ NMR (C_6D_6, 298K): δ 145.0 (s, *ortho*-C), 121.8 (s, *para*-CH), 110.8 (d, $^3J_{PC}$ = 10.9 Hz, *meta*-CH), 34.6 (d, $^1J_{PP}$ = 21.8 Hz, *CH*CH_2), 34.5 (d, $^1J_{PP}$ = 21.8 Hz, *CH*CH_2), 32.6 (broad m, CH_2), 31.1 (broad m, CH_2), 27.5 (s, CH_2), 27.4 (s, CH_2), 26.0 (s, CH_2).

Elemental analysis: calcd. for $C_{24}H_{30}O_4P_2$: C, 64.86; H, 6.80. Found: C, 64.62; H, 6.791.

IR (solid): $\tilde{\upsilon}$ = 3061, 2921, 2847, 1743, 1619, 1599, 1479, 1451, 1343, 1239, 1202, 1178, 1100, 1007, 968, 844, 788, 761, 748, 734, 695, 615, 584, 556, 521, 502, 456.

Preparation of 3a

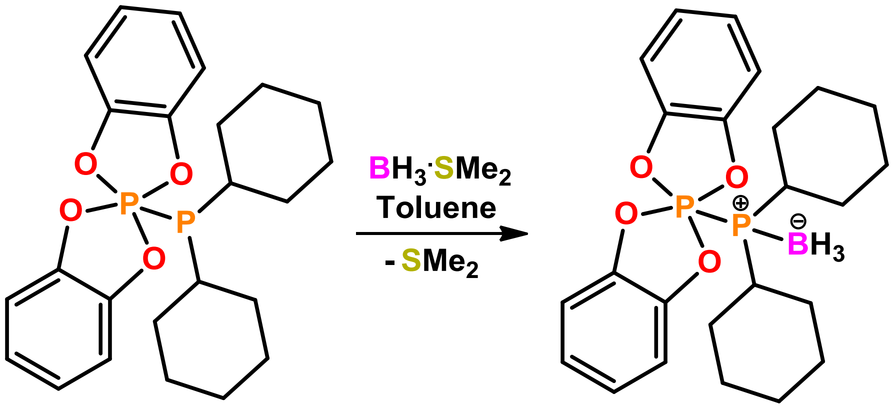

3 3a

To a solution of **3** (111 mg, 0.250 mmol) in 5 cm^3 of toluene $BH_3{\cdot}SMe_2$ (0.025 cm^3, 19 mg, 0.25 mmol) was added at room temperature. The solution was stirred for an hour. $^{31}P\{^1H\}$ NMR of the reaction mixture revealed the complete conversion of **3** into **3a**. The solvent was evaporated, and the residue was dried under a vacuum (0.01 Torr) at 50°C for 2 hours to remove all volatiles and afford **3a** as white solid (107 mg, 0.233 mmol) in 93% yield.

NMR:

$^{31}P\{^1H\}$ NMR (C_6D_6, 298K): δ 30.1 (broad m, PCy_2), -2.7 (d, $^1J_{PP}$ = 21.8 Hz, $Pcat_2$).

^{11}B (C_6D_6, 298K): -41.4 (broad m).

^1H NMR (C_6D_6, 298K): δ 6.82 (m, 4H, *m*-C*H*), 6.64 (m, 4H, *p*-C*H*), 1.85 – 0.84 (broad overlapped m, 22H, CH and CH_2), 1.18 (overlapped broad m, 3H, B*H_3*).

$^{13}C\{^1H\}$ NMR (C_6D_6, 298K): δ 143.9 (d, $^2J_{PC}$ = 3.6 Hz, *ortho*-C), 122.2 (s, *para*-CH), 110.9 (d, $^3J_{PC}$ = 13.6 Hz, *meta*-CH), 33.0 (dd, $^1J_{PC}$ = 30.0 Hz, $^2J_{PC}$ = 4.5 Hz, *CH*CH_2), 26.9 (s, CH_2), 26.7 (d, $^3J_{PC}$ = 3.6 Hz, CH_2), 26.7 (s, CH_2), 26.6 (d, $^3J_{PC}$ = 2.7 Hz, CH_2), 25.7 (s, CH_2).

Elemental analysis: calcd. for $C_{24}H_{33}BO_4P_2$: C, 62.90; H, 7.26. Found: C, 62.81; H, 7.429.

IR (solid): $\tilde{\upsilon}$ = 3067, 2930, 2848, 2398 (**B-H**), 1482, 1444, 1343, 1266, 1246, 1185, 1104, 1069, 1007, 916, 846, 830, 818, 782, 742, 607, 532, 501, 452.

Preparation of 4

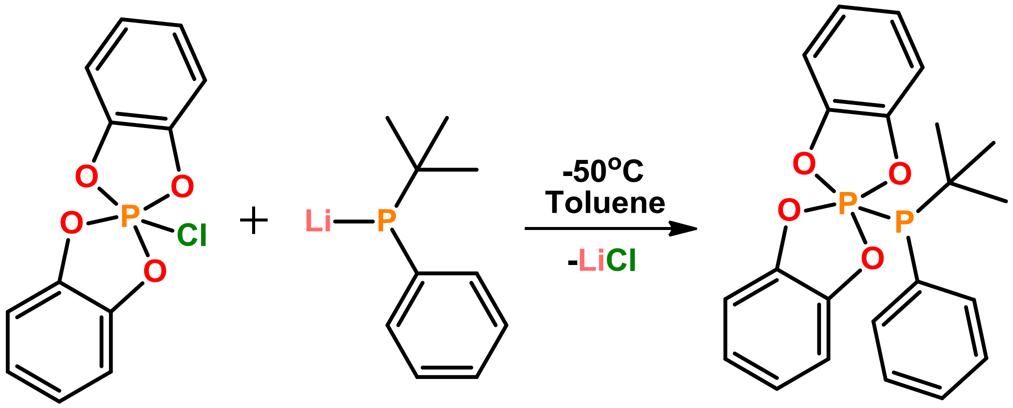

4

To a suspension of *t*BuPhPLi (0.172 g, 1.00 mmol) in 15 cm^3 of toluene cooled to -50°C, a suspension of cat_2PCl (0.283 g, 1.00 mmol) in 5 cm^3 of toluene was added dropwise. The reaction mixture was stirred at -50°C for 2 hours and then allowed to warm to room temperature and kept stirring for an hour. The reaction mixture was filtered and the solution was concentrated and left at -20°C to afford X-ray quality crystals of **4**. The crystalline product was separated and dried under vacuum (0.01 Torr), giving **4** as analytically pure white solid. Yield 87% (0.357 g, 0.866 mmol).

NMR:

$^{31}P\{^1H\}$ NMR (C_6D_6, 298K): δ 29.3 (d, $^1J_{PP}$ = 334.2 Hz, P*t*BuPh), 8.3 (d, $^1J_{PP}$ = 334.2 Hz, $Pcat_2$).

^1H NMR (C_6D_6, 298K): δ 7.60 (m, 2H, *ortho*-CH, P*t*BuPh), 6.94 – 6.85 (overlapped m, 3H, *p,m*-C*H,* P*t*BuPh), 6.75 (m, 2H, *m*-C*H,* $Pcat_2$), 6.63 (m, 2H, *m*-C*H,* $Pcat_2$), 6.54 (m, 4H, *p*-C*H,* $Pcat_2$), 1.23 (dd, 9H, $^3J_{PH}$ = 13.9 Hz, $^4J_{PH}$ = 2.4 Hz, C*H_3*).

$^{13}C\{^1H\}$ NMR (C_6D_6, 298K): δ 146.3 (s, *ortho*-C, $Pcat_2$), 143.8 (s, *ortho*-C, $Pcat_2$), 136.8 (dd, $^2J_{PC}$ = 21.8 Hz, $^3J_{PC}$ = 17.3 Hz, *ortho*-CH, P*t*BuPh), 130.9 (dd, $^1J_{PC}$ = 10.9 Hz, $^2J_{PC}$ = 5.4 Hz, *ipso*-C, P*t*BuPh), 129.5 (s, *para*-CH, P*t*BuPh), 127.7 (d, $^3J_{PC}$ = 8.2 Hz, *meta*-CH, P*t*BuPh), 122.4 (s, *para*-CH, $Pcat_2$), 120.9 (s, *para*-CH, $Pcat_2$), 111.0 (d, $^3J_{PC}$ = 12.7 Hz, *meta*-CH, $Pcat_2$), 110.4 (d, $^3J_{PC}$ = 9.0 Hz, *meta*-CH, $Pcat_2$), 34.6 (dd, $^1J_{PC}$ = 20.9 Hz, $^2J_{PC}$ = 6.3 Hz, *C*$(CH_3)_3$), 29.2 (dd, $^2J_{PC}$ = 14.5 Hz, $^3J_{PC}$ = 10.9 Hz, C(CH_3)$_3$).

Elemental analysis: calcd. for $C_{22}H_{22}O_4P_2$: C, 64.08; H, 5.38. Found: C, 63.83; H, 5.390.

IR (solid): $\tilde{\upsilon}$ = 3055, 2968, 2948, 2896, 2862, 1619, 1597, 1481, 1434, 1363, 1345, 1279, 1256, 1239, 1203, 1176, 1100, 1008, 973, 864, 845, 787, 766, 745, 733, 700, 640, 620, 569, 558, 520, 494, 453.

Preparation of 4a

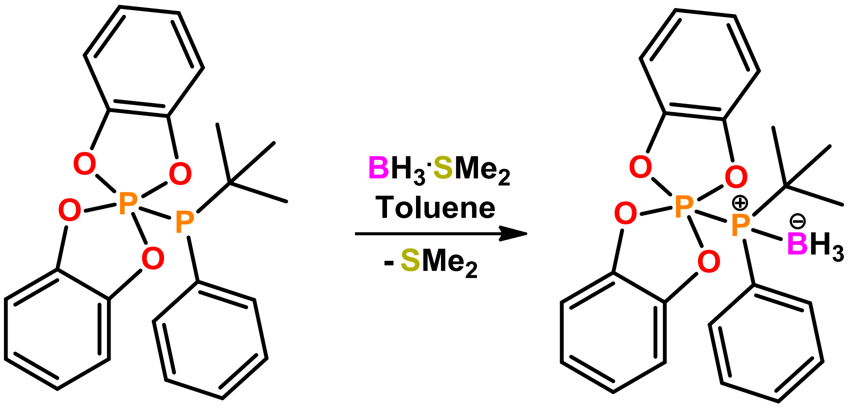

To a solution of **4** (103 mg, 0.250 mmol) in 5 cm^3 of toluene $BH_3 \cdot SMe_2$ (0.025 cm^3, 19 mg, 0.25 mmol) was added at room temperature. The solution was stirred for an hour. $^{31}P\{^1H\}$ NMR of the reaction mixture revealed the complete conversion of **4** into **4a**. The solvent was evaporated, and the residue was dried under a vacuum (0.01 Torr) at 50°C for 2 hours to remove all volatiles and afford **4a** as white solid (101 mg, 0.237 mmol) in 95% yield. X-ray quality crystals were grown from toluene solution layered with pentane at 4°C.

NMR:

$^{31}P\{^1H\}$ NMR (C_6D_6, 298K): δ 56.8 (d, $^1J_{PP}$ = 116.2 Hz, P*t*BuPh), -4.1 (d, $^1J_{PP}$ = 116.2 Hz, $Pcat_2$).

^{11}B (C_6D_6, 298K): -40.1 (broad m).

^1H NMR (C_6D_6, 298K): δ 7.77 (m, 2H, *ortho*-CH, P*t*BuPh), 6.92 (m, 1H, *p*-C*H,* P*t*BuPh), 6.83 (m, 2H, *m*-C*H,* P*t*BuPh), 6.71 (m, 2H, *m*-C*H,* $Pcat_2$), 6.54 (overlapped m, 6H, *m, p*-C*H,* $Pcat_2$), 1.74 (broad m, 3H, BH_3), 1.24 (dd, 9H, $^3J_{PH}$ = 15.3 Hz, $^4J_{PH}$ = 1.7 Hz, CH_3).

$^{13}C\{^1H\}$ NMR (C_6D_6, 298K): δ 145.7 (s, *ortho*-C, $Pcat_2$), 143.7 (s, *ortho*-C, $Pcat_2$), 133.9 (dd, $^2J_{PC}$ = 8.2 Hz, $^3J_{PC}$ = 7.3 Hz, *ortho*-CH, P*t*BuPh), 131.0 (s, *para*-CH, P*t*BuPh), 127.9 (d, $^3J_{PC}$ = 10.0 Hz, *meta*-CH, P*t*BuPh), 126.2 (dd, $^1J_{PC}$ = 44.5 Hz, $^2J_{PC}$ = 4.5 Hz, *ipso*-C, P*t*BuPh), 122.8 (s, *para*-CH, $Pcat_2$), 121.6 (s, *para*-CH, $Pcat_2$), 111.1 (d, $^3J_{PC}$ = 13.6 Hz, *meta*-CH, $Pcat_2$), 110.5 (d, $^3J_{PC}$ = 10.9 Hz, *meta*-CH, $Pcat_2$), 35.0 (dd, $^1J_{PC}$ = 20.0 Hz, $^2J_{PC}$ = 3.6 Hz, $C(CH_3)_3$), 27.0 (dd, $^2J_{PC}$ = 5.4 Hz, $^3J_{PC}$ = 2.7 Hz, $C(CH_3)_3$).

Elemental analysis: calcd. for $C_{22}H_{25}BO_4P_2$: C, 62.00; H, 5.91. Found: C, 61.85; H, 5.905.

IR (solid): $\tilde{\nu}$ = 3070, 3033, 2969, 2903, 2870, 2424 (**B-H**), 2393 (**B-H**), 2356 (**B-H**), 1625, 1599, 1479, 1438, 1366, 1341, 1256, 1237, 1195, 1130, 1105, 1054, 1011, 902, 869, 794, 737, 700, 625, 607, 569, 553, 519, 491, 449.

Preparation of 5

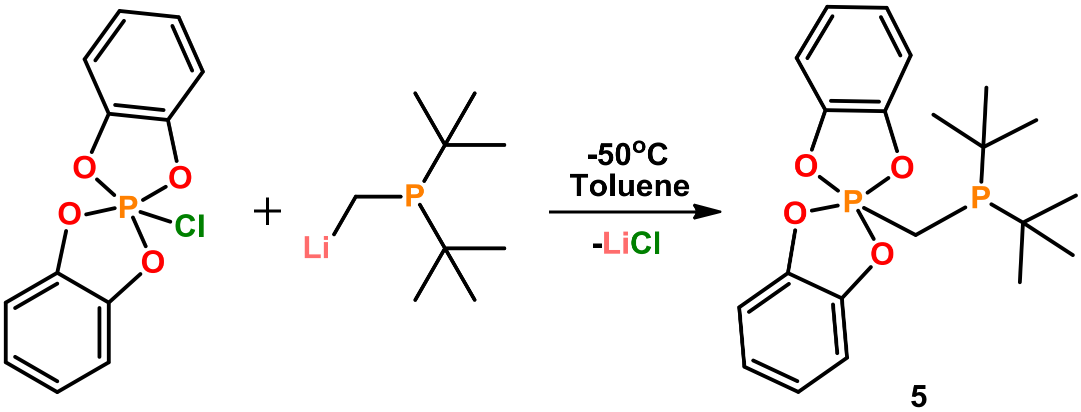

To a suspension of tBu$_2$PCH$_2$Li (0.166 g, 1.00 mmol) in 15 cm^3 of toluene cooled to -50°C, a suspension of cat$_2$PCl (0.283 g, 1.00 mmol) in 5 cm^3 of toluene was added dropwise. The reaction mixture was stirred at -50°C for 4 hours and then allowed to warm to room temperature and kept stirring for an hour. The reaction mixture was filtered and the solution was concentrated and left at -20°C to afford X-ray quality crystals of **5**. The crystalline product was separated and dried under vacuum (0.01 Torr), giving **5** as analytically pure white solid. Yield 76% (0.310 g, 0.763 mmol).

NMR:

^{31}P{^{1}H} NMR (C$_6$D$_6$, 298K): δ 17.4 (d, $^2J_{PP}$ = 87.2 Hz, PtBu$_2$), 2.7 (d, $^2J_{PP}$ = 87.2 Hz, Pcat$_2$).

^{1}H NMR (C$_6$D$_6$, 298K): δ 6.84 (m, 4H, *m*-C*H*), 6.64 (m, 4H, *p*-C*H*), 2.26 (dd, 2H, $^2J_{P(III)H}$ = 18.3 Hz, $^2J_{P(V)H}$ = 1.6 Hz, C*H*$_2$), 0.97 (d, 18H, $^3J_{PH}$ = 11.5 Hz, C*H*$_3$).

^{13}C{^{1}H} NMR (C$_6$D$_6$, 298K): δ 144.9 (d, $^2J_{PC}$ = 3.6 Hz, *ortho*-C), 121.6 (s, *para*-CH), 110.3 (d, $^3J_{PC}$ = 12.7 Hz, *meta*-CH), 31.9 (dd, $^1J_{PC}$ = 25.4 Hz, $^3J_{PC}$ = 10.0 Hz, *C*(CH$_3$)$_3$), 28.9 (d, $^2J_{PC}$ = 15.4 Hz, C(*CH*$_3$)$_3$), 28.0 (dd, $^1J_{P(V)C}$ = 176.2 Hz, $^1J_{P(III)C}$ = 45.4 Hz, CH$_2$).

Elemental analysis: calcd. for C$_{21}$H$_{28}$O$_4$P$_2$: C, 62.06; H, 6.94. Found: C, 61.96; H, 6.904.

IR (solid): $\tilde{\upsilon}$ = 2977, 2945, 2896, 2863, 1484, 1354, 1281, 1262, 1245, 1211, 1134, 1103, 1009, 909, 862, 815, 791, 737, 702, 682, 532, 503, 446.

Preparation of 5a

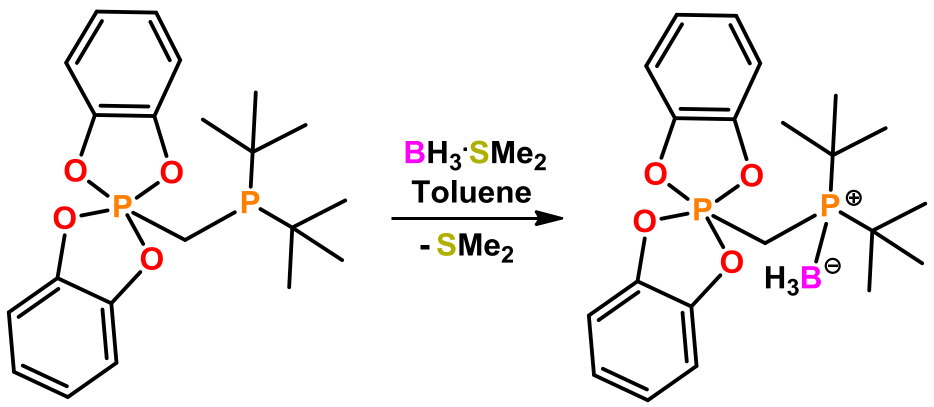

5 **5a**

To a solution of **5** (102 mg, 0.250 mmol) in 5 cm^3 of toluene $BH_3 \cdot SMe_2$ (0.025 cm^3, 19 mg, 0.25 mmol) was added at room temperature. The solution was stirred for an hour. $^{31}P\{^1H\}$ NMR of the reaction mixture revealed the complete conversion of **5** into **5a**. The solvent was evaporated, and the residue was dried under a vacuum (0.01 Torr) at 50°C for 2 hours to remove all volatiles and afford **5a** as white solid (99 mg, 0.236 mmol) in 94% yield. X-ray quality crystals were grown from toluene solution layered with pentane at 4°C.

NMR:

$^{31}P\{^1H\}$ NMR (C_6D_6, 298K): δ 49.9 (broad m, P*t*Bu_2), -1.9 (s, $Pcat_2$).

^{11}B (C_6D_6, 298K): -41.6 (broad m).

^1H NMR (C_6D_6, 298K): δ 6.86 (m, 4H, *m*-C*H*), 6.65 (m, 4H, *p*-C*H*), 2.52 (dd, 2H, $^2J_{PH}$ = 21.2 Hz, $^2J_{PH}$ = 12.0 Hz, CH_2), 1.11 (overlapped broad m, 3H, BH_3), 1.02 (d, 18H, $^3J_{PH}$ = 13.0 Hz, CH_3).

$^{13}C\{^1H\}$ NMR (C_6D_6, 298K): δ 144.4 (d, $^2J_{PC}$ = 3.0 Hz, *ortho*-C), 122.0 (s, *para*-CH), 110.9 (d, $^3J_{PC}$ = 14.0 Hz, *meta*-CH), 33.3 (dd, $^1J_{PC}$ = 23.5 Hz, $^3J_{PC}$ = 6.6 Hz, *C*(CH_3)$_3$), 27.4 (d, $^2J_{PC}$ = 2.2 Hz, C(CH_3)$_3$), 26.0 (dd, $^1J_{P(V)C}$ = 181.9 Hz, $^1J_{P(III)C}$ = 12.5 Hz, CH_2).

Elemental analysis: calcd. for $C_{21}H_{31}BO_4P_2$: C, 60.02; H, 7.44. Found: C, 59.86; H, 7.34.

IR (solid): $\tilde{\nu}$ = 2958, 2382 (**B-H**), 1483, 1356, 1343, 1307, 1243, 1204, 1184, 1151, 1100, 1065, 1008, 983, 900, 871, 827, 785, 759, 748, 733, 711, 692, 639, 535, 494, 455.

Preparation of 5b

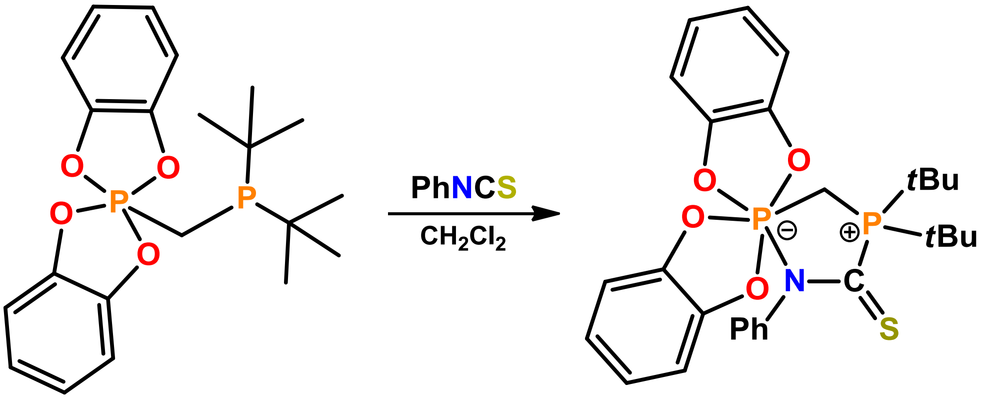

5 **5b**

To a solution of **5** (203 mg, 0.5 mmol) in 5 cm^3 of CH_2Cl_2, PhNCS (0.054 cm^3, 60 mg, 0.5 mmol) was added at room temperature. The solution was stirred for 3 days. $^{31}P\{^1H\}$ NMR of the reaction mixture revealed the complete conversion of **5** into **5b**. The solvent was evaporated, and the residue was dried under a vacuum (0.01 Torr) at 50°C for an hour to remove all volatiles and afford **5b** as yellow solid (260 mg, 0.480 mmol) in 96% yield. X-ray quality crystals were grown from dichloromethane solution layered with pentane at 4°C.

NMR:

$^{31}P\{^1H\}$ NMR (CDCl$_3$, 298K): δ 29.6 (s, P*t*Bu$_2$), -91.2 (s, Pcat$_2$).

^1H NMR (CDCl$_3$, 298K): δ 7.35 (m, 1H, *o*-C*H,* NPh), 7.28 (m, 1H, *o*-C*H,* NPh), 7.07 (m, 1H, *m*-C*H,* NPh), 6.87 (m, 1H, *m*-C*H,* NPh), 6.75 (m, 1H, *p*-C*H*, Pcat$_2$), 6.70 – 6.64 (overlapped m, 5H, *m,p*-C*H*, Pcat$_2$), 6.58 (m, 1H, *p*-C*H*, Pcat$_2$), 6.45 (m, 1H, *p*-C*H*, Pcat$_2$), 6.18 (m, 1H, *p*-C*H*, NPh), 5.95 (m, 1H, *m*-C*H*, Pcat$_2$), 2.64 (ddd, 1H, $^2J_{HH}$ = 10.0 Hz, $^2J_{PH}$ = 19.2 Hz, $^2J_{PH}$ = 15.6 Hz, C*H$_2$*), 2.22 (ddd, 1H, $^2J_{HH}$ = 10.0 Hz, $^2J_{PH}$ = 16.0 Hz, $^2J_{PH}$ = 16.0 Hz, C*H$_2$*), 1.67 (d, 9H, $^3J_{PH}$ = 16.3 Hz, C*H$_3$*), 1.53 (d, 9H, $^3J_{PH}$ = 15.9 Hz, C*H$_3$*).

$^{13}C\{^1H\}$ NMR (CDCl$_3$, 298K): δ 179.6 (dd, $^1J_{PC}$ = 66.3 Hz, $^2J_{PC}$ = 18.2 Hz, C=S), 146.3 (d, $^2J_{PC}$ = 10.9 Hz, *ipso*-C, NPh), 145.7 (d, $^2J_{PC}$ = 3.6 Hz, *o*-C, Pcat$_2$), 144.7 (d, $^2J_{PC}$ = 3.6 Hz, *o*-C, Pcat$_2$), 144.5 (d, $^2J_{PC}$ = 2.7 Hz, *o*-C, Pcat$_2$), 129.1 (s, *o*-CH, NPh), 127.8 (s, *o*-CH, NPh), 126.7 (s, *m*-CH, NPh), 126.1 (s, *m*-CH, NPh), 125.3 (s, *p*-CH, NPh), 120.8 (s, *p*-CH, Pcat$_2$), 120.6 (s, *p*-CH, Pcat$_2$), 119.9 (s, *p*-CH, Pcat$_2$), 119.5 (s, *p*-CH, Pcat$_2$), 111.2 (d, $^3J_{PC}$ = 13.6 Hz, *m*-CH, Pcat$_2$), 111.0 (d, $^3J_{PC}$ = 14.5 Hz, *m*-CH, Pcat$_2$), 110.0 (d, $^3J_{PC}$ = 20.0 Hz, *m*-CH, Pcat$_2$), 108.8 (d, $^3J_{PC}$ = 14.5 Hz, *m*-CH, Pcat$_2$), 37.7 (dd, $^1J_{PC}$ = 30.9 Hz, $^3J_{PC}$ = 2.7 Hz, *C*(CH$_3$)$_3$), 37.3 (dd, $^1J_{PC}$ = 30.0 Hz, $^3J_{PC}$ = 2.7 Hz, *C*(CH$_3$)$_3$), 28.1 (s, C(*CH$_3$*)$_3$), 27.9 (s, C(*CH$_3$*)$_3$), 20.1 (dd, $^1J_{P(V)C}$ = 164.4 Hz, $^1J_{P(III)C}$ = 29.1 Hz, CH$_2$).

Elemental analysis: calcd. for $C_{28}H_{33}NO_4P_2S$: C, 62.10; H, 6.14; N, 2.59; S, 5.92. Found: , 62.01; H, 6.203; N, 2.61; S, 5.887.

IR (solid): $\tilde{\nu}$ = 3059, 3005, 2990, 2951, 1485 (**C=N**), 1386, 1371, 1355, 1261 (**C=S**), 1243, 1201, 1150, 1102, 1065, 1012, 886, 873, 817, 775, 722, 688, 674, 655, 629, 603, 590, 534, 513, 496, 470, 448.

Preparation of 6

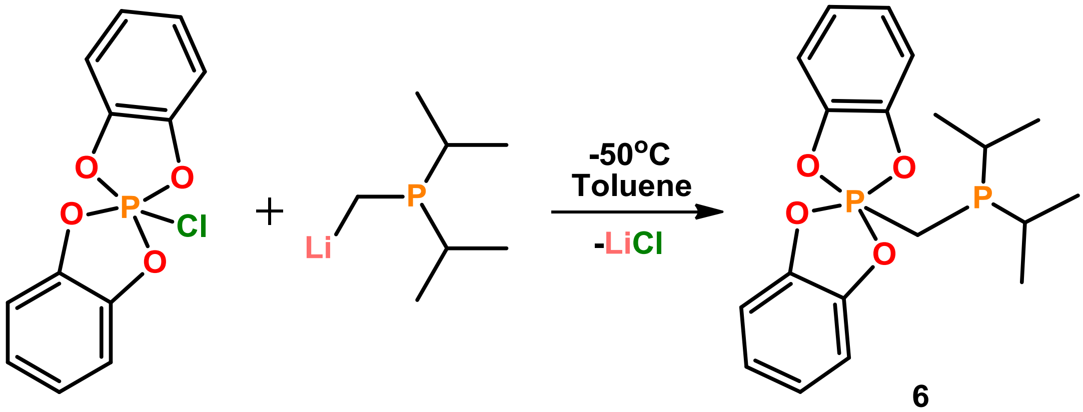

6

To a suspension of iPr$_2$CH$_2$PLi (0.138 g, 1.00 mmol) in 15 cm^3 of toluene cooled to -50°C, a suspension of cat$_2$PCl (0.283 g, 1.00 mmol) in 5 cm^3 of toluene was added dropwise. The reaction mixture was stirred at -50°C for 2 hours and then allowed to warm to room temperature and kept stirring for an hour. The solvent was evaporated, and the residue was dried under a vacuum (0.01 mmHg) for 30 minutes at 50°C to remove all volatiles. The crude product was dissolved in 10 cm^3 of petroleum ether and filtered. Removal of the solvent under vacuum afforded 0.360 g (0.952 mmol) of **6** as a yellowish powder in 95% yield. X-ray quality crystals were grown from concentrated toluene solution layered with pentane at -20°C.

NMR:

^{31}P{^{1}H} NMR (C$_6$D$_6$, 298K): δ 2.6 (d, $^2J_{PP}$ = 72.7 Hz, Pcat$_2$), -4.4 (d, $^2J_{PP}$ = 72.7 Hz, PiPr$_2$).

^{1}H NMR (C$_6$D$_6$, 298K): δ 6.85 (m, 4H, *m*-C*H*), 6.64 (m, 4H, *p*-C*H*), 2.04 (dd, 2H, $^2J_{P(III)H}$ = 17.9 Hz, $^2J_{P(V)H}$ = 1.6 Hz, C*H$_2$*), 1.43 (sept, 2H, $^3J_{HH}$ = 7.0 Hz, C*H*CH$_3$), 0.87 (dd, 6H, $^3J_{HH}$ = 7.0 Hz, $^3J_{PH}$ = 14.7 Hz, C*H$_3$*), 0.85 (dd, 6H, $^3J_{HH}$ = 7.0 Hz, $^3J_{PH}$ = 11.3 Hz, C*H$_3$*).

^{13}C{^{1}H} NMR (C$_6$D$_6$, 298K): δ 144.8 (d, $^2J_{PC}$ = 3.6 Hz, *ortho*-C), 121.6 (s, *para*-CH), 110.4 (d, $^3J_{PC}$ = 13.6 Hz, *meta*-CH), 27.2 (dd, $^1J_{P(V)C}$ = 173.5 Hz, $^1J_{P(III)C}$ = 40.9 Hz, CH$_2$), 24.1 (dd, $^1J_{PC}$ = 16.3 Hz, $^3J_{PC}$ = 10.9 Hz, *CH*CH$_3$), 18.9 (d, $^2J_{PC}$ = 17.3 Hz, CH$_3$), 17.8 (d, $^2J_{PC}$ = 10.0 Hz, CH$_3$).

Elemental analysis: calcd. for C$_{19}$H$_{24}$O$_4$P$_2$: C, 60.32; H, 6.39. Found: C, 60.32; H, 6.398.

IR (solid): $\tilde{\upsilon}$ = 3051, 2952, 2925, 2866, 1744, 1604, 1484, 1461, 1385, 1353, 1279, 1244, 1207, 1152, 1099, 1010, 951, 874, 823, 730, 637, 545.

Preparation of 6a

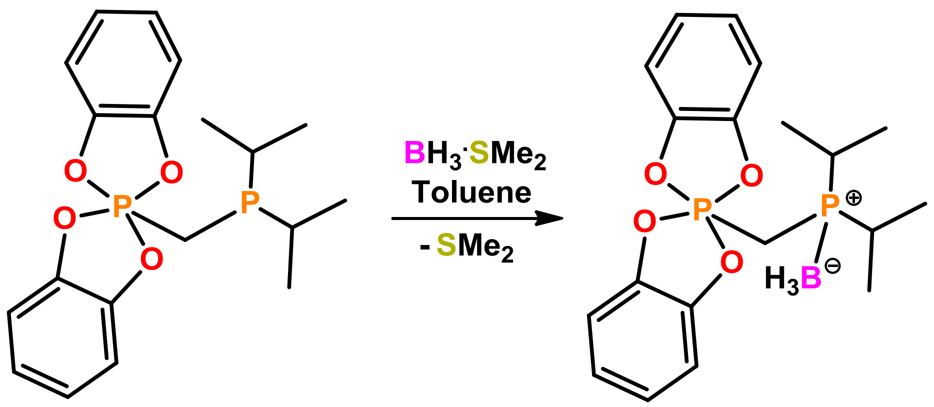

To a solution of **6** (95 mg, 0.250 mmol) in 5 cm^3 of toluene $BH_3{\cdot}SMe_2$ (0.025 cm^3, 19 mg, 0.25 mmol) was added at room temperature. The solution was stirred for an hour. $^{31}P\{^1H\}$ NMR of the reaction mixture revealed the complete conversion of **6** into **6a**. The solvent was evaporated, and the residue was dried under a vacuum (0.01 Torr) at 50°C for 2 hours to remove all volatiles and afford **6a** as colourless oil (89 mg, 0.227 mmol) in 91% yield. The oily product slowly solidifies at -20°C to afford X-ray quality crystals of **6a**.

NMR:

$^{31}P\{^1H\}$ NMR (C_6D_6, 298K): δ 37.6 (broad m, , P*i*Pr$_2$), -3.0 (d, $^2J_{PP}$ = 21.8 Hz, Pcat$_2$).

^{11}B (C_6D_6, 298K): -42.4 (broad m).

^1H NMR (C_6D_6, 298K): δ 6.80 (m, 4H, *m*-C*H*), 6.64 (m, 4H, *p*-C*H*), 2.36 (dd, 2H, $^2J_{PH}$ = 19.9 Hz, $^2J_{PH}$ = 11.5 Hz, C*H$_2$*), 1.83 (sept, 1H, $^3J_{HH}$ = 7.1 Hz, C*H*CH$_3$), 1.80 (sept, 1H, $^3J_{HH}$ = 7.1 Hz, C*H*CH$_3$), 1.03 (overlapped m, 3H B*H$_3$*), 0.95 (dd, 6H, $^3J_{HH}$ = 7.1 Hz, $^3J_{PH}$ = 15.9 Hz, C*H$_3$*), 0.91 (dd, 6H, $^3J_{HH}$ = 7.1 Hz, $^3J_{PH}$ = 15.8 Hz, C*H$_3$*).

$^{13}C\{^1H\}$ NMR (C_6D_6, 298K): δ 143.9 (d, $^2J_{PC}$ = 3.6 Hz, *ortho*-C), 122.1 (s, *para*-CH), 110.9 (d, $^3J_{PC}$ = 13.6 Hz, *meta*-CH), 27.1 (dd, $^1J_{P(V)C}$ = 172.6 Hz, $^1J_{P(III)C}$ = 14.5 Hz, CH$_2$), 23.1 (d, $^1J_{PC}$ = 30.9 Hz, *CH*CH$_3$), 23.0 (d, $^1J_{PC}$ = 30.0 Hz, *CH*CH$_3$), 16.8 (s, CH$_3$), 16.6 (s, CH$_3$).

Elemental analysis: calcd. for $C_{19}H_{27}BO_4P_2$: C, 58.19; H, 6.94. Found: C, 57.98; H, 6.888.

IR (solid): $\tilde{\nu}$ = 3061, 2964, 2935, 2875, 2388 (**B-H**), 1625, 1481, 1392, 1380, 1354, 1343, 1278, 1244, 1201, 1163, 1116, 1101, 1063, 1009, 907, 875, 820, 792, 741, 700, 632, 575, 531, 502, 466, 448.

Preparation of 7

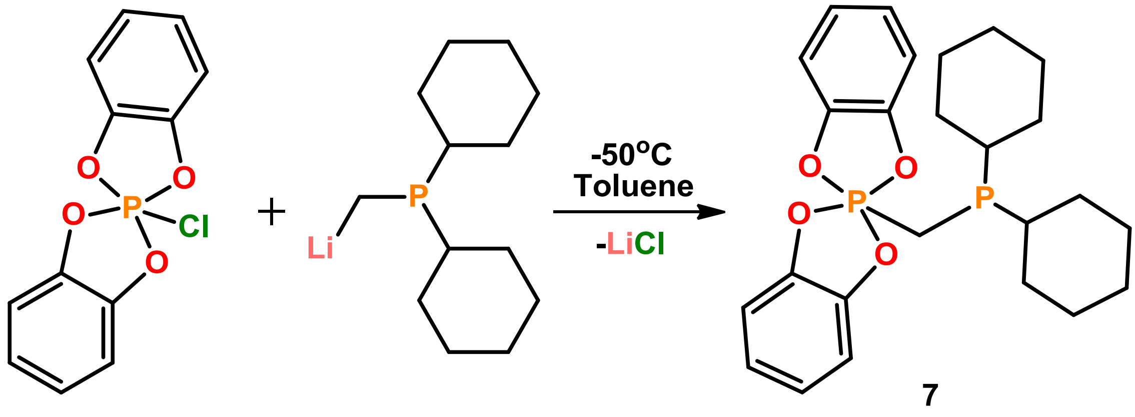

To a suspension of Cy_2CH_2PLi (0.218 g, 1.00 mmol) in 15 cm^3 of toluene cooled to -50°C, a suspension of cat_2PCl (0.283 g, 1.00 mmol) in 5 cm^3 of toluene was added dropwise. The reaction mixture was stirred at -50°C for 3 hours and then allowed to warm to room temperature and kept stirring for an hour. The solvent was evaporated, and the residue was dried under a vacuum (0.01 mmHg) for 30 minutes at 50°C to remove all volatiles. The crude product was dissolved in 10 cm^3 of petroleum ether and filtered. Removal of the solvent under vacuum afforded 0.390 g (0.851 mmol) of **7** as a white powder in 85% yield. X-ray quality crystals were grown from concentrated toluene solution at -20°C.

NMR:

$^{31}P\{^1H\}$ NMR (C_6D_6, 298K): δ 3.0 (d, $^2J_{PP}$ = 72.7 Hz, $Pcat_2$), -12.8 (d, $^2J_{PP}$ = 72.7 Hz, PCy_2).

^1H NMR (C_6D_6, 298K): δ 6.87 (m, 4H, *m*-C*H*), 6.65 (m, 4H, *p*-C*H*), 2.16 (dd, 1H, $^2J_{P(III)H}$ = 17.8 Hz, $^2J_{P(V)H}$ = 1.0 Hz, PCH_2P), 1.63 – 1.05 (broad overlapped m, 22H, CH and CH_2 of Cy groups).

$^{13}C\{^1H\}$ NMR (C_6D_6, 298K): δ 144.8 (d, $^2J_{PC}$ = 2.7 Hz, *ortho*-C), 121.6 (s, *para*-CH), 110.4 (d, $^3J_{PC}$ = 13.6 Hz, *meta*-CH), 33.9 (dd, $^1J_{PC}$ = 17.3 Hz, $^3J_{PC}$ = 10.0 Hz, *C*HCH_2), 29.2 (d, $^2J_{PC}$ = 14.5 Hz, CH_2), 28.3 (d, $^2J_{PC}$ = 10.0 Hz, CH_2), 27.2 (dd, $^1J_{P(V)C}$ = 172.6 Hz, $^1J_{P(III)C}$ = 41.8 Hz, P*C*H_2P), 27.0 (d, $^3J_{PC}$ = 10.0 Hz, CH_2), 26.9 (d, $^3J_{PC}$ = 7.3 Hz, CH_2), 26.3 (s, CH_2).

Elemental analysis: calcd. for $C_{25}H_{32}O_4P_2$: C, 65.49; H, 7.04. Found: C, 65.50; H, 7.112.

IR (solid): $\tilde{\upsilon}$ = 3059, 2920, 2852, 1484, 1450, 1355, 1247, 1220, 1180, 1128, 1100, 1068, 1006, 908, 863, 807, 792, 762, 731, 719, 674, 631, 525, 504, 447.

Preparation of 7a

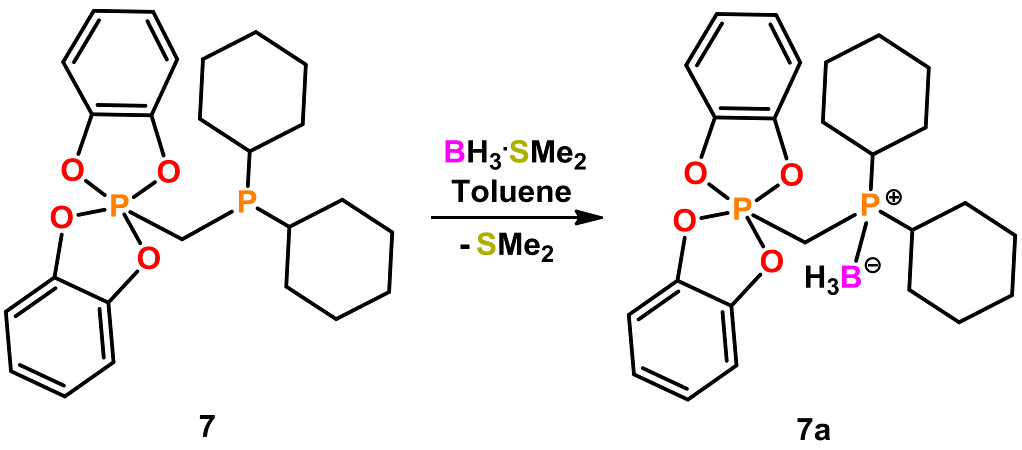

To a solution of **7** (115 mg, 0.250 mmol) in 5 cm^3 of toluene $BH_3{\cdot}SMe_2$ (0.025 cm^3, 19 mg, 0.25 mmol) was added at room temperature. The solution was stirred for an hour. $^{31}P\{^1H\}$ NMR of the reaction mixture revealed the complete conversion of **7** into **7a**. The solvent was evaporated, and the residue was dried under a vacuum (0.01 Torr) at 50°C for 2 hours to remove all volatiles and afford **7a** as a white solid (105 mg, 0.222 mmol) in 89% yield.

NMR:

$^{31}P\{^1H\}$ NMR (C_6D_6, 298K): δ 30.1 (broad m, PCy_2), -2.7 (d, $^2J_{PP}$ = 21.8 Hz, $Pcat_2$).

^{11}B (C_6D_6, 298K): -41.4 (broad m).

^1H NMR (C_6D_6, 298K): δ 6.82 (m, 4H, *m*-C*H*), 6.64 (m, 4H, *p*-C*H*), 2.48 (dd, 1H, $^2J_{PH}$ = 19.7 Hz, $^2J_{PH}$ = 11.0 Hz, PC*H*$_2$P), 1.85 – 0.84 (broad overlapped m, 22H, CH and CH_2 of Cy groups), 1.20 (broad overlapped m, 3H, B*H*$_3$).

$^{13}C\{^1H\}$ NMR (C_6D_6, 298K): δ 143.9 (d, $^2J_{PC}$ = 3.6 Hz, *ortho*-C), 122.2 (s, *para*-CH), 110.9 (d, $^3J_{PC}$ = 13.6 Hz, *meta*-CH), 33.0 (dd, $^1J_{PC}$ = 29.1 Hz, $^3J_{PC}$ = 4.5 Hz, *C*HCH$_2$), 26.9 (s, CH_2), 26.7 (dd, $^1J_{P(V)C}$ = 170.7 Hz, $^1J_{P(III)C}$ = 15.4 Hz, P*C*H$_2$P), 26.7 (s, CH_2), 26.7 (d, $^2J_{PC}$ = 11.8 Hz, CH_2), 26.6 (d, $^2J_{PC}$ = 11.8 Hz, CH_2), 25.7 (s, CH_2).

Elemental analysis: calcd. for $C_{25}H_{35}O_4BP_2$: C, 63.58; H, 7.47. Found: C, 63.35; H, 7.412.

IR (solid): $\tilde{\nu}$ = 3067, 2930, 2848, 2398 (**B-H**), 1482, 1444, 1379, 1355, 1344, 1268, 1245, 1185, 1104, 1070, 1068, 1007, 928, 916, 845, 830, 818, 782, 737, 608, 531, 501, 452.

X-ray structures analysis

General methods

The X-ray diffraction data were collected on an IPDS 2T dual-beam diffractometer (STOE&Cie GmbH, Darmstadt, Germany) at 120.0(2) K with Mo-$K_α$ radiation of a microfocus X-ray source (GeniX 3D Mo HighFlux, Xenocs, Sassenage, France, 50 kV, 1.0 mA, λ = 0.71069 Å) for **1a**, **1b**, **2a**, **3**, **4**, **4a**, **5a**, **5b**, **6**, **6a**, **7**. For structures **1** and **5**, we used Cu-$K_α$ radiation of a microfocus X-ray source (GeniX 3D Cu HighFlux, Xenocs, 50 kV, 0.6 mA, λ = 1.54186 Å). The choice was dictated by the Mo lamp's temporary unavailability (failure). During the experiment, every crystal was thermostated in a nitrogen stream at 120 K using CryoStream-800 devices (Oxford CryoSystem, UK). Data collection and data reduction were controlled by X-Area 1.75 program.[3] The structure was solved by the SHELXT method[4,5] and refined using the program packages Olex2[6,7] and SHELX-2015.[4,5] All non-hydrogen atoms were modelled as anisotropic, and all H-atoms were refined as isotropic. Hydrogen atoms were placed in idealized positions and refined with the usual restraints of the riding model. All B-H atoms were found in the Fourier map and were refined without constraints.

Crystallographic data for all structures reported in this paper have been deposited with the Cambridge Crystallographic Data Centre as supplementary publication No. CCDC 2174618-2174629, and 2177995. The data can be obtained free of charge from The Cambridge Crystallographic Data Centre via www.ccdc.cam.ac.uk/structures.

Specific details for individual structures

Most structures contain one molecule in the asymmetric unit (Z' = 1) except **5, 6** and **6a,** where two symmetry-independent molecules are present Z'=2. For structure **1,** the use of copper radiation caused difficulties with reaching completeness in our 2-circle goniometer of IPDS system. Structure **1a** was refined with a large fragment (BH_3-P-P-Bu) of the molecule disordered over two positions with site occupation factors of 0.432(10)/0.568(10). Structures **1b**, **2a**, **3**, **4** and **4a** were determined without special treatment. The structure of **5** crystallizing in a polar group $Pca2_1$ contained two symmetry-independent moieties, and each one was disordered. At the first site, with P1-P2, two *tert*-butyl groups were refined as disordered over two positions with sof 0.676(18)/0.324(18). The second moiety, with P3-P4, was refined with one *tert*-Bu group disordered over two positions with sof 0.710(22)/0.290(22) and one $C_6H_4O_2$ residue disordered with sof 0.731(14)/0.269(14). Checkcif procedure generated an alert about thermal ellipsoids, but the attribution of elements is correct, and the alert may be an artefact related to unaccounted structural modulations seen in the diffraction pattern. Additionally, it proved helpful to refine the extinction coefficient and apply twinning refinement with a 4% contribution of the part related by inversion. Structures **5a** and **5b** were determined without any special treatment. Structures **6**, **6a** and **7** have relatively high refinement indices, which stem from the weak diffraction power of the available specimens (I/σ ca 10). Not the best structure quality indicators may also result from some unresolved minor disorder, modulations or twinning, naturally occurring in real crystals and hard to model. Therefore, those structures were also refined without any special treatment.

TABLE S1. CRYSTAL DATA AND STRUCTURE REFINEMENT FOR **1**, **1A** AND **1B**

	**1**	**1a**	**1b**
CCDC	2174618	2174619	2174620
Empirical formula	$C_{20}H_{26}O_4P_2$	$C_{20}H_{29}BO_4P_2$	$C_{27}H_{31}NO_4P_2S$
M_r [g mol^{-1}]	392.35	406.18	527.53
Crystal system	Monoclinic	Orthorhombic	Monoclinic
Space group	*P2₁/n*	*Pna2₁*	*P2₁/n*
a [Å]	8.6878(15)	22.9450(13)	10.0787(9)
b [Å]	23.131(3)	10.9571(8)	18.257(2)
c [Å]	10.583(2)	8.4739(4)	15.2379(14)
α [°]	90	90	90
β [°]	111.331(13)	90	108.063(7)
γ [°]	90	90	90
V [Å^3]	1981.0(6)	2130.4(2)	2665.6(5)
Z	4	4	4
Calculated density [Mg m^{-3}]	1.316	1.266	1.314
T [K]	120	120	120
μ [mm^{-1}]	2.18	0.23	0.28
Crystal size/mm^3	0.32 × 0.07 × 0.06	0.32 × 0.17 × 0.15	0.28 × 0.11 × 0.10
λ [Å]	1.54186 (CuKα)	0.71073 (MoKα)	0.71073 (MoKα)
F(000)	832	864	1112
S	1.1	1.06	1.01
R_{int}	0.037	0.024	0.058
No. of measured, independent, observed [I > 2σ(I)] reflections	10666, 3349, 2536	31132, 5486, 4940	37183, 7201, 5171
$R[F^2 > 2\sigma(F^2)]$	0.041	0.049	0.061
$wR(F^2)$	0.107,	0.125	0.18
Largest diff. peak/hole / e Å^{-3}	0.31/-0.36	0.44/-0.34	0.70/-0.64

TABLE S2. CRYSTAL DATA AND STRUCTURE REFINEMENT FOR **2A**, **3** AND **4**

	**2a**	**3**	**4**
CCDC	2174621	2174622	2174623
Empirical formula	$C_{18}H_{25}BO_4P_2$	$C_{24}H_{30}O_4P_2$	$C_{22}H_{22}O_4P_2$
M_r [g mol^{-1}]	378.13	444.42	412.33
Crystal system	Monoclinic	Monoclinic	Orthorhombic
Space group	$P2_1/n$	$P2_1/n$	$P2_12_12_1$
a [Å]	7.3688(5)	10.129(3)	20.565 (3)
b [Å]	13.9941(8)	18.112(5)	16.1130 (19)
c [Å]	19.0799(13)	12.615(3)	5.9863 (7)
α [°]	90	90	90
β [°]	100.015(5)	105.93(2)	90
γ [°]	90	90	90
V [Å^3]	1937.5(2)	2225.4(11)	1983.7 (5)
Z	4	4	4
Calculated density [Mg m^{-3}]	1.296	1.327	1.381
T [K]	120	120	120
μ [mm^{-1}]	0.24	0.22	0.25
Crystal size/mm^3	0.27 × 0.12 × 0.04	0.51 × 0.12 × 0.07	0.27 × 0.07 × 0.03
λ [Å]	0.71073 (MoKα)	0.71073 (MoKα)	0.71073 (MoKα)
F(000)	800	944	864
S	1.07	1.1	1.03
R_{int}	0.03	0.034	0.062
No. of measured, independent, observed [I > 2σ(I)] reflections	27736, 5217, 4525	32474, 6014, 5134	14641, 5356, 3822
$R[F^2 > 2\sigma(F^2)]$	0.032	0.033	0.05
$wR(F^2)$	0.091	0.092	0.114
Largest diff. peak/hole / e Å^{-3}	0.38/-0.32	0.35/-0.31	0.30/-0.41

TABLE S3. CRYSTAL DATA AND STRUCTURE REFINEMENT FOR **4A**, **5** AND **5A**

	**4a**	**5**	**5a**
CCDC	2177995	2174624	2174625
Empirical formula	$C_{22}H_{25}BO_4P_2$	$C_{21}H_{28}O_4P_2$	$C_{21}H_{31}BO_4P_2$
M_r [g mol^{-1}]	426.17	406.37	420.21
Crystal system	Orthorhombic	Orthorhombic	Orthorhombic
Space group	*Pbca*	$Pca2_1$	*Pbca*
a [Å]	12.288(5)	18.331(6)	14.5349(17)
b [Å]	14.368(4)	12.857(5)	14.9552(17)
c [Å]	24.368(7)	17.949(12)	20.279(2)
α [°]	90	90	90
β [°]	90	90	90
γ [°]	90	90	90
V [Å^3]	4302(2)	4230(4)	4408.1(9)
Z	8	8	8
Calculated density [Mg m^{-3}]	1.316	1.276	1.266
T [K]	120	120	120
μ [mm^{-1}]	0.23	2.06	0.22
Crystal size/mm^3	0.25 × 0.04 × 0.03	0.13 × 0.06 × 0.03	0.24 × 0.13 × 0.11
λ [Å]	0.71073 (MoKα)	1.54186 (CuKα)	0.71073 (MoKα)
F(000)	1792	1728	1792
S	1.03	1.19	1.03
R_{int}	0.082	0.019	0.056
No. of measured, independent, observed [I > 2σ(I)] reflections	24290, 3830, 3100	24181, 7175, 6951	25912, 5943, 4167
$R[F^2 > 2\sigma(F^2)]$	0.052	0.055	0.044
$wR(F^2)$	0.143	0.114	0.116
Largest diff. peak/hole / e Å^{-3}	0.43/-0.46	0.41/-0.52	0.63/-0.34

TABLE S4. CRYSTAL DATA AND STRUCTURE REFINEMENT FOR **5B**, **6** AND **6A**

	**5b**	**6**	**6a**
CCDC	2174626	2174627	2174628
Empirical formula	$C_{28}H_{33}NO_4P_2S{\cdot}CH_2Cl_2$	$C_{19}H_{24}O_4P_2$	$C_{19}H_{27}BO_4P_2$
M_r [g mol^{-1}]	626.48	378.32	392.15
Crystal system	Monoclinic	Triclinic	Monoclinic
Space group	$P2_1/n$	P-1	$P2_1/c$
a [Å]	8.7580(5)	7.4393(9)	14.6174(9)
b [Å]	15.7466(6)	13.645(2)	13.6302(7)
c [Å]	22.1509(10)	19.525(3)	20.7436(14)
α [°]	90	87.268(12)	90
β [°]	95.136(4)	82.127(11)	101.745(5)
γ [°]	90	75.559(11)	90
V [Å^3]	3042.5(3)	1901.1(5)	4046.4(4)
Z	4	4	8
Calculated density [Mg m^{-3}]	1.368	1.322	1.288
T [K]	120	120	120
μ [mm^{-1}]	0.42	0.25	0.24
Crystal size/mm^3	0.31 × 0.27 × 0.09	0.32 × 0.12 × 0.03	0.21 × 0.18 × 0.04
λ [Å]	0.71073 (MoKα)	0.71073 (MoKα)	0.71073 (MoKα)
F(000)	1312	800	1664
S	1.06	1.1	1.04
R_{int}	0.026	0.090	0.094
No. of measured, independent, observed [I > 2σ(I)] reflections	17373, 8124, 6352	17327, 7460, 5777	40804, 10857, 6102
$R[F^2 > 2\sigma(F^2)]$	0.04	0.1	0.07
$wR(F^2)$	0.107	0.298	0.217
Largest diff. peak/hole / e Å^{-3}	0.66/-0.61	0.86/-0.79	0.57/-0.61

TABLE S5. CRYSTAL DATA AND STRUCTURE REFINEMENT FOR **7**

	**7**
CCDC	2174629
Empirical formula	$C_{25}H_{32}O_4P_2$
M_r [g mol^{-1}]	458.44
Crystal system	Monoclinic
Space group	$P2_1/c$
a [Å]	22.672(2)
b [Å]	9.1033(17)
c [Å]	11.308(4)
α [°]	90
β [°]	100.355(16)
γ [°]	90
V [Å^3]	2295.8 (10)
Z	4
Calculated density [Mg m^{-3}]	1.326
T [K]	120
μ [mm^{-1}]	0.22
Crystal size/mm^3	0.27 × 0.19 × 0.08
λ [Å]	0.71073 (MoKα)
F(000)	976
S	1.1
R_{int}	0.053
No. of measured, independent, observed [I > 2σ(I)] reflections	9157, 4121, 3434
$R[F^2 > 2\sigma(F^2)]$	0.102
$wR(F^2)$	0.286
Largest diff. peak/hole / e Å^{-3}	1.04/-0.86

Single crystal X-ray structure analysis of 1

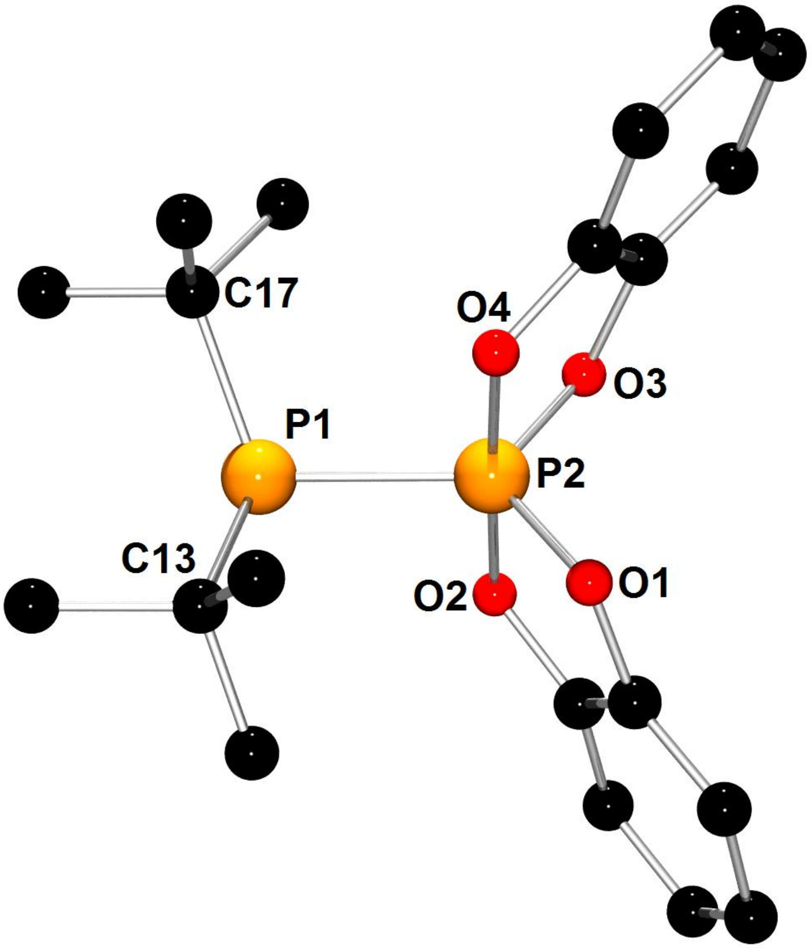

FIG. S1. MOLECULAR STRUCTURE OF **1**

TABLE S6. SELECTED STRUCTURAL PARAMETERS OF **1**

**Bond lengths [Å]**		**Bond angles [°]**	
P1-P2	2.236(1)	P1-P2-O2	86.88(7)
P1-C13	1.904(2)	P1-P2-O4	95.42(7)
P1-C17	1.894(4)	O1-P2-O2	90.35(9)
P2-O1	1.639(2)	O1-P2-O4	87.28(9)
P2-O2	1.735(2)	O3-P2-O4	91.51(9)
P2-O3	1.638(2)	O2-P2-O3	88.41(9)
P2-O4	1.716(2)	P1-P2-O1	126.71(7)
		P1-P2-O3	119.88(7)
		O1-P2-O3	113.21(9)

Single crystal X-ray structure analysis of 1a

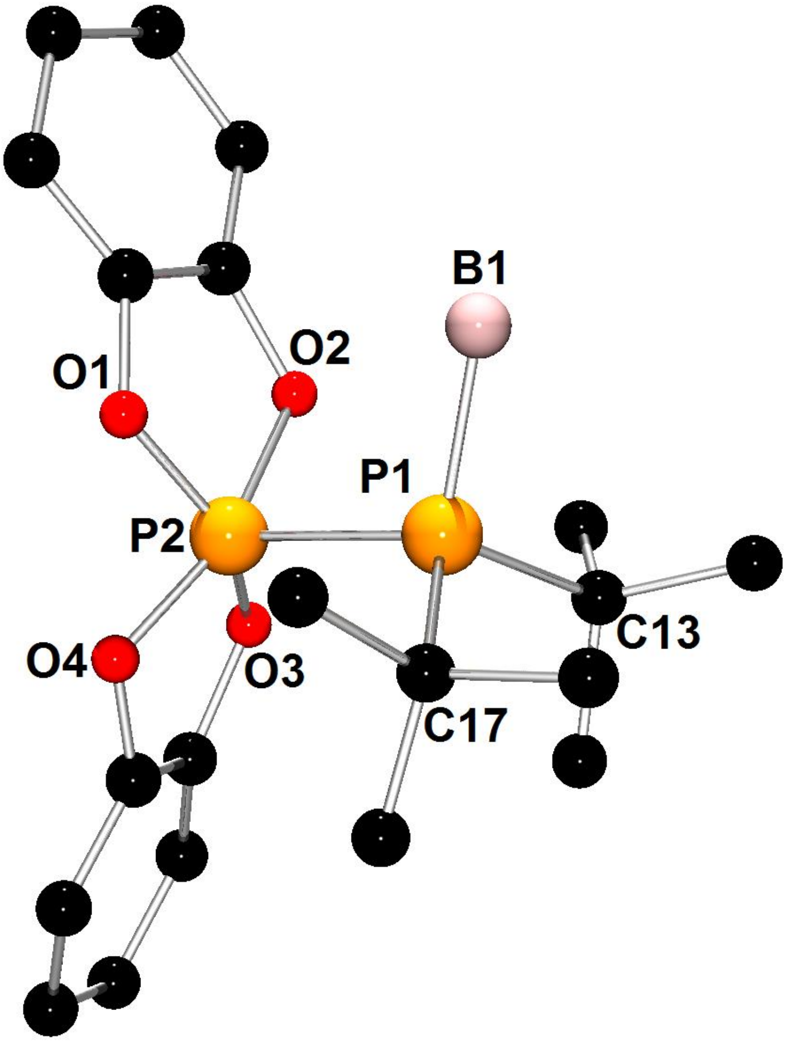

FIG. S2. MOLECULAR STRUCTURE OF **1A**

TABLE S7. SELECTED STRUCTURAL PARAMETERS OF **1A**

**Bond lengths [Å]**		**Bond angles [°]**	
P1-P2	2.266(9)	P1-P2-O2	91.6(3)
P1-B1	1.95(1)	P1-P2-O4	107.0(5)
P1-C13	1.990(6)	O1-P2-O2	87.1(4)
P1-C17	1.89(1)	O1-P2-O4	85.0(5)
P2-O1	1.610(7)	O2-P2-O3	83.3(4)
P2-O2	1.801(8)	O3-P2-O4	93.0(5)
P2-O3	1.556(8)	P1-P2-O1	104.3(4)
P2-O4	1.66(1)	P1-P2-O3	111.0(4)
		O1-P2-O3	143.5(5)

Single crystal X-ray structure analysis of 1b

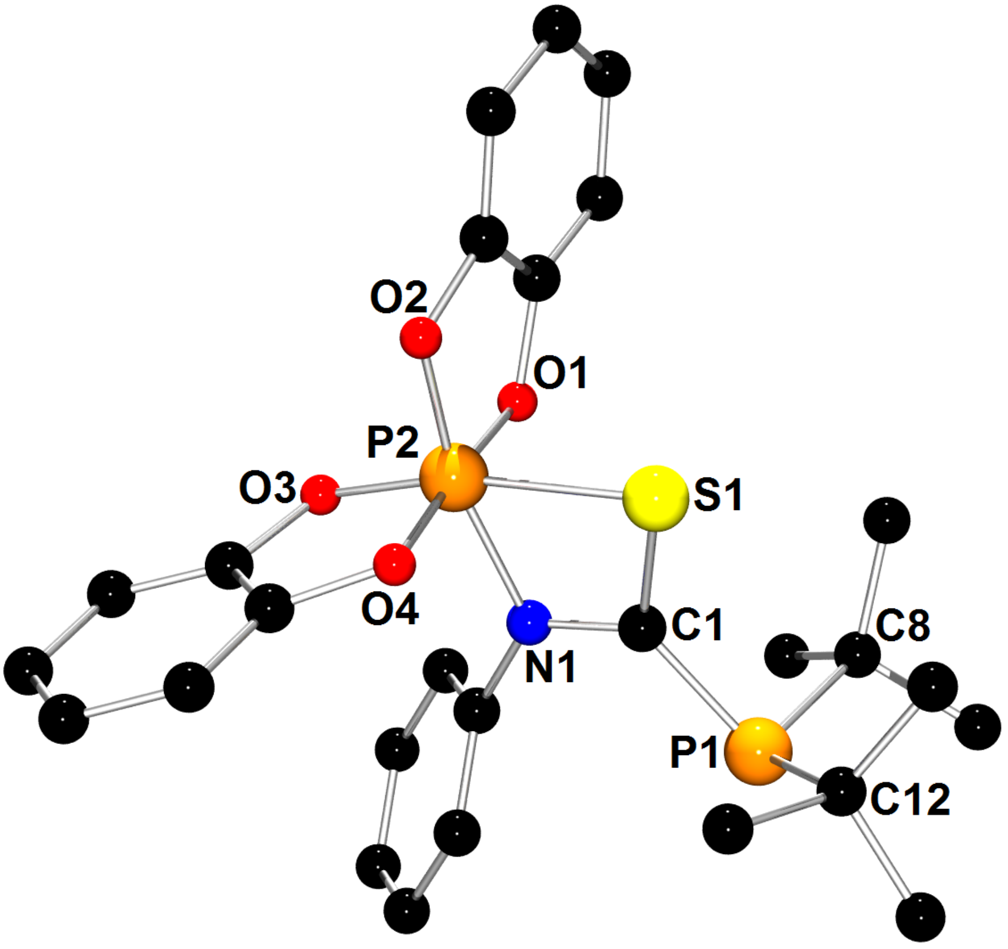

FIG. S3. MOLECULAR STRUCTURE OF **1B**

TABLE S8. SELECTED STRUCTURAL PARAMETERS OF **1B**

**Bond lengths [Å]**		**Bond angles [°]**		**Dihedrals [°]**	
P1-C1	1.847(2)	N1-P2-O1	86.53(8)	S1-C1-N1-P2	-0.0(2)
P1-C8	1.886(2)	S1-P2-O1	90.16(6)	P1-C1-N1-C2	5.5(3)
P1-C12	1.881(2)	O1-P2-O2	92.10(8)		
P2-S1	2.2808(8)	O1-P2-O3	89.76(8)		
P2-O1	1.701(1)	N1-P2-O4	90.37(8)		
P2-O2	1.673(2)	S1-P2-O4	87.18(6)		
P2-O3	1.669(2)	O2-P2-O4	90.45(8)		
P2-O4	1.699(1)	O3-P2-O4	92.27(8)		
P2-N1	1.875(2)	N1-P2-S1	71.73(6)		
N1-C1	1.318(3)	N1-P2-O3	94.68(8)		
S1-C1	1.717(3)	O2-P2-O3	99.65(8)		
N1-C2	1.437(3)	S1-P2-O2	93.95(6)		
		C1-S1-P2	77.24(7)		
		S1-C1-N1	107.4(2)		
		C1-N1-P2	103.7(1)		

Single crystal X-ray structure analysis of 2a

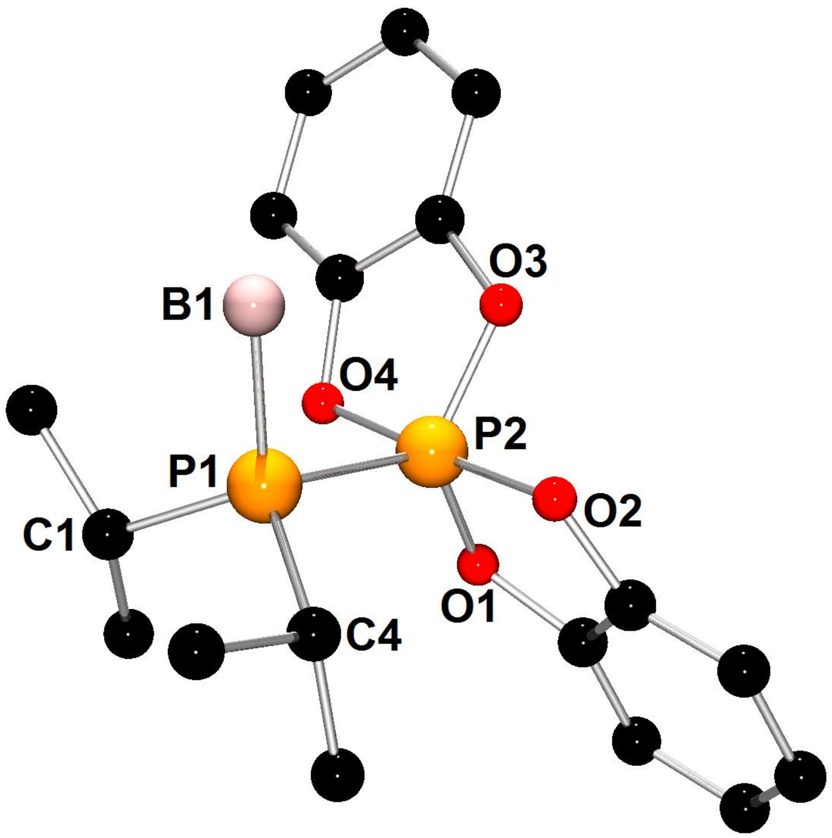

FIG. S4. MOLECULAR STRUCTURE OF **2A**

TABLE S9. SELECTED STRUCTURAL PARAMETERS OF **2A**

**Bond lengths [Å]**		**Bond angles [°]**	
P1-P2	2.2051(5)	P1-P2-O4	91.35(3)
P1-B1	1.937(1)	P1-P2-O2	91.13(3)
P1-C1	1.842(1)	O1-P2-O2	91.63(4)
P1-C4	1.839(1)	O1-P2-O4	88.08(4)
P2-O1	1.6283(8)	O2-P2-O3	86.62(4)
P2-O2	1.7094(9)	O3-P2-O4	91.20(4)
P2-O3	1.6270(9)	P1-P2-O1	122.07(3)
P2-O4	1.7072(9)	P1-P2-O3	115.92(3)
		O1-P2-O3	122.00(4)

Single crystal X-ray structure analysis of 3

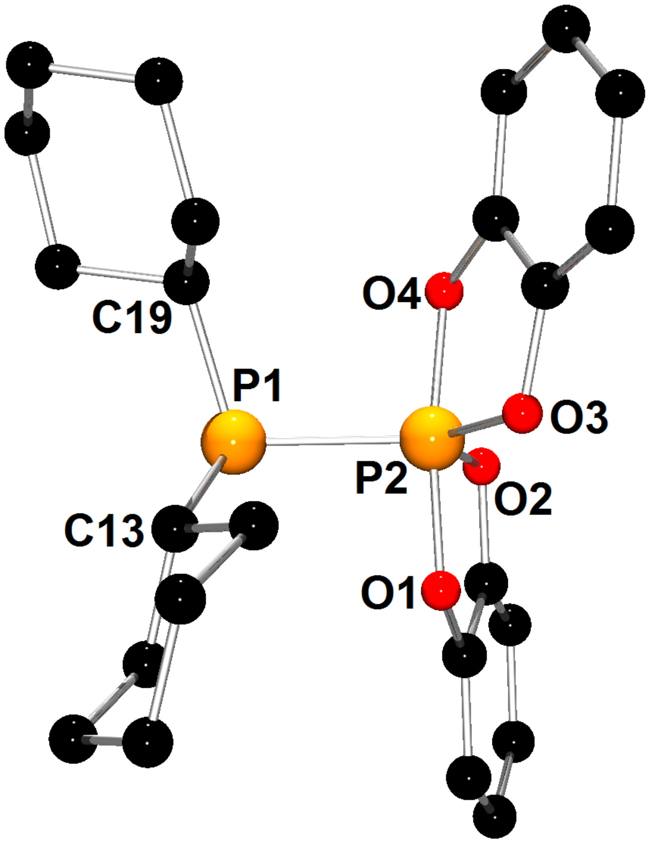

FIG. S5. MOLECULAR STRUCTURE OF **3**

TABLE S10. SELECTED STRUCTURAL PARAMETERS OF **3**

**Bond lengths [Å]**		**Bond angles [°]**	
P1-P2	2.1902(6)	P1-P2-O1	92.03(3)
P1-C13	1.864(1)	P1-P2-O4	95.67(3)
P1-C19	1.853(1)	O1-P2-O2	90.91(4)
P2-O1	1.717(1)	O2-P2-O4	84.61(4)
P2-O2	1.637(1)	O1-P2-O3	86.25(5)
P2-O3	1.639(1)	O3-P2-O4	90.82(5)
P2-O4	1.723(1)	P1-P2-O2	110.95(3)
		P1-P2-O3	125.18(4)
		O2-P2-O3	123.86(5)

Single crystal X-ray structure analysis of 4

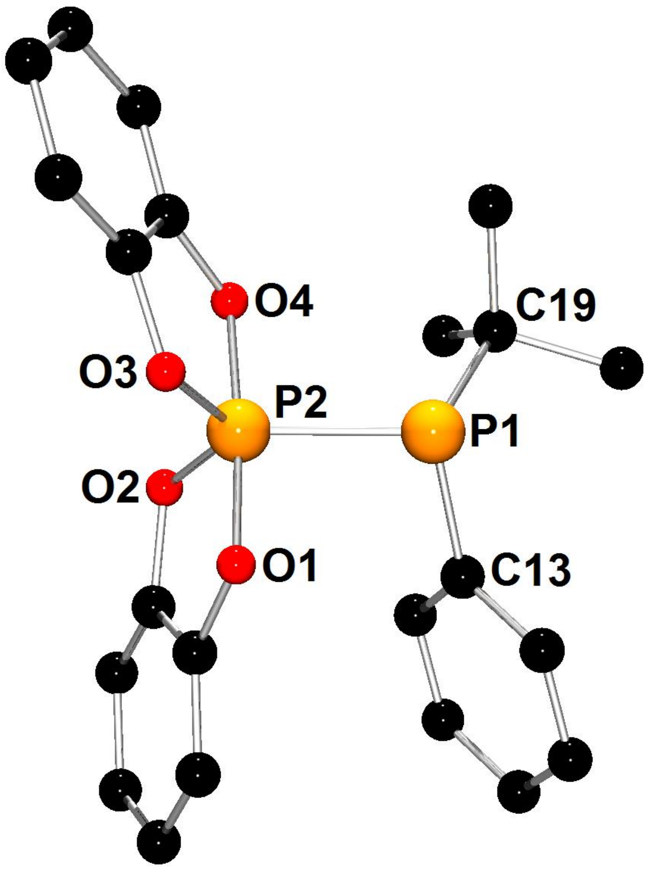

FIG. S6. MOLECULAR STRUCTURE OF **4**

TABLE S11. SELECTED STRUCTURAL PARAMETERS OF **4**

**Bond lengths [Å]**		**Bond angles [°]**	
P1-P2	2.191(1)	P1-P2-O1	88.87(9)
P1-C13	1.830(4)	P1-P2-O4	95.5(1)
P1-C19	1.882(4)	O1-P2-O2	91.1(1)
P2-O1	1.718(3)	O2-P2-O4	87.7(1)
P2-O2	1.640(3)	O1-P2-O3	86.2(1)
P2-O3	1.642(3)	O3-P2-O4	90.6(1)
P2-O4	1.711(3)	P1-P2-O2	124.5(1)
		P1-P2-O3	115.9(1)
		O2-P2-O3	119.4(1)

Single crystal X-ray structure analysis of 4a

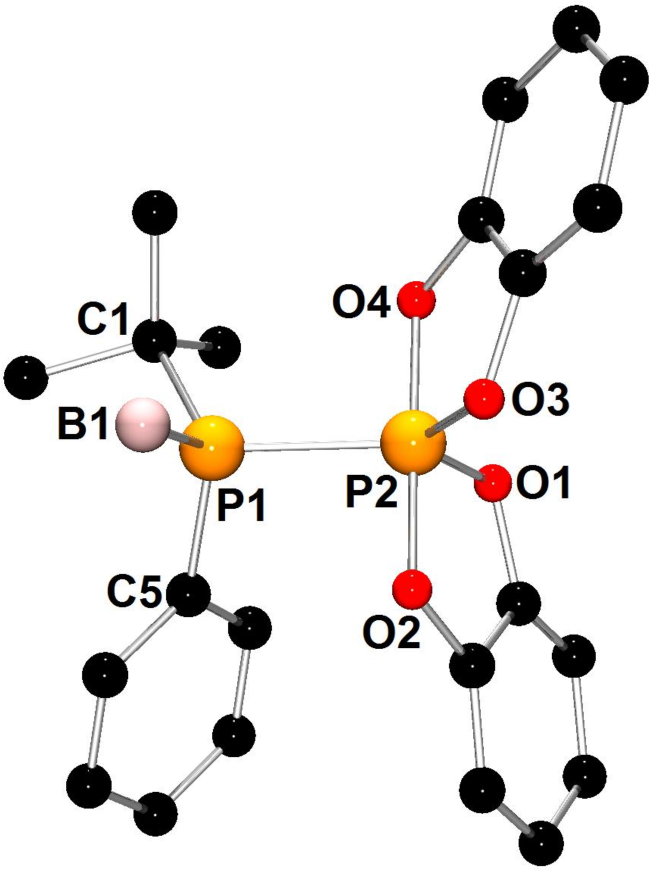

FIG. S7. MOLECULAR STRUCTURE OF **4A**

TABLE S12. SELECTED STRUCTURAL PARAMETERS OF **4A**

**Bond lengths [Å]**		**Bond angles [°]**	
P1-P2	2.219(1)	P1-P2-O4	91.53(7)
P1-B1	1.933(4)	P1-P2-O2	89.22(6)
P1-C1	1.868(3)	O1-P2-O4	88.73(9)
P1-C5	1.812(3)	O1-P2-O2	91.64(9)
P2-O1	1.621(2)	O2-P2-O3	87.59(9)
P2-O2	1.708(2)	O3-P2-O4	91.28(9)
P2-O3	1.628(2)	P1-P2-O1	120.64(7)
P2-O4	1.701(2)	P1-P2-O3	120.52(7)
		O1-P2-O3	118.82(9)

Single crystal X-ray structure analysis of 5

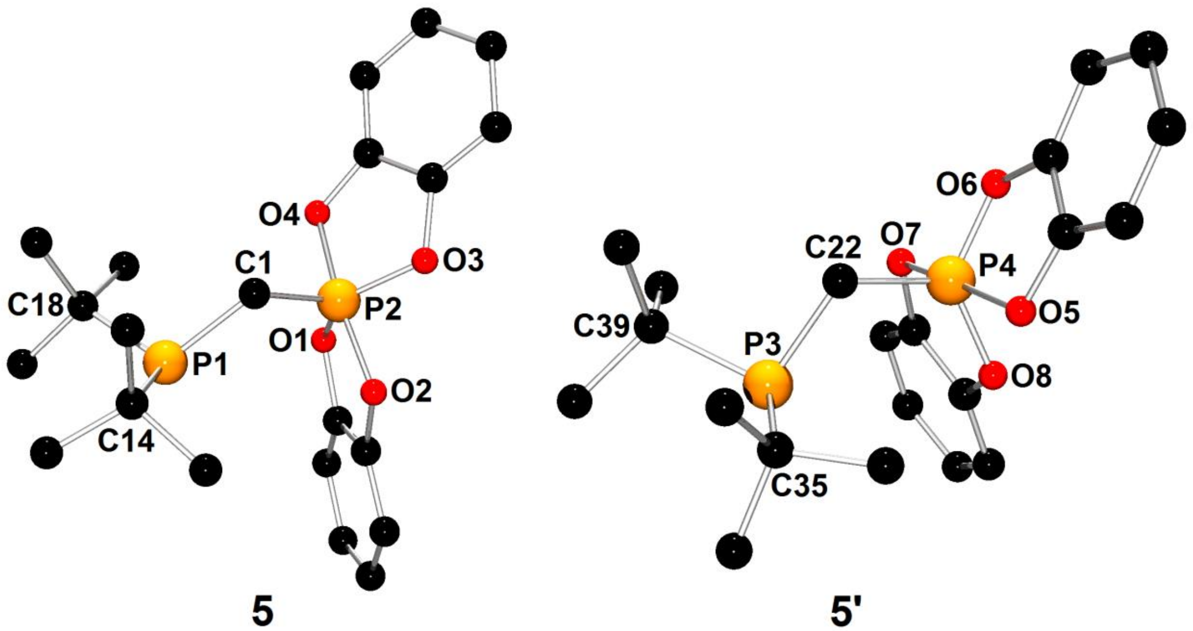

5 5'

FIG. S8. MOLECULAR STRUCTURE OF **5** AND **5'**

TABLE S13. SELECTED STRUCTURAL PARAMETERS OF **5**

**Bond lengths [Å]**		**Bond angles [°]**	
P1-C1	1.862(6)	P1-C1-P2	115.6(3)
P2-C1	1.795(6)	C1-P2-O4	95.7(2)
P1-C18	1.808(9)	C1-P2-O2	93.7(2)
P1-C14	1.98(1)	O1-P2-O4	84.6(2)
P2-O1	1.626(4)	O1-P2-O2	90.7(2)
P2-O2	1.714(5)	O2-P2-O3	86.2(2)
P2-O3	1.641(4)	O3-P2-O4	90.3(2)
P2-O4	1.713(4)	C1-P2-O1	119.5(2)
		C1-P2-O3	112.9(2)
		O1-P2-O3	127.6(2)

TABLE S14. SELECTED STRUCTURAL PARAMETERS OF **5'**

**Bond lengths [Å]**		**Bond angles [°]**	
P3-C22	1.867(7)	P3-C22-P4	113.7(3)
P4-C22	1.803(6)	C22-P4-O5	96.0(3)
P3-C35	1.94(1)	C22-P4-O7	92.5(3)
P3-C39	1.897(6)	O5-P4-O6	91.4(4)
P4-O5	1.750(8)	O6-P4-O7	89.8(3)
P4-O6	1.558(9)	O5-P4-O8	80.3(3)
P4-O7	1.710(4)	O7-P4-O8	91.1(2)
P4-O8	1.631(5)	C22-P4-O8	119.5(3)
		C22-P4-O6	113.4(4)
		O6-P4-O8	127.0(4)

Single crystal X-ray structure analysis of 5a

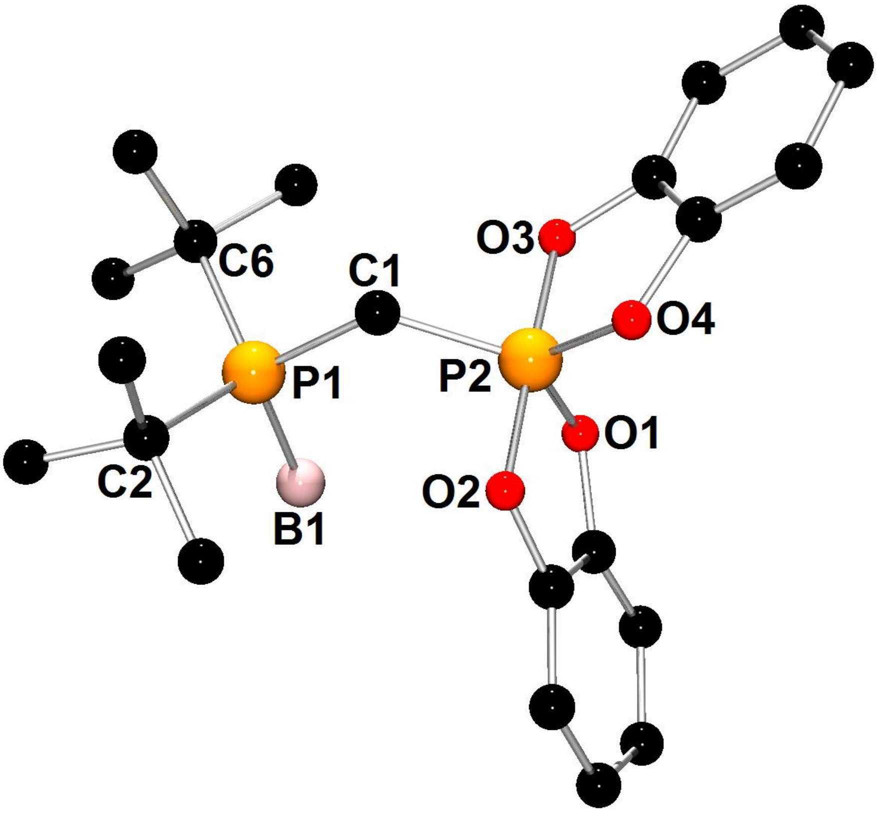

FIG. S9. MOLECULAR STRUCTURE OF **5A**

TABLE S15. SELECTED STRUCTURAL PARAMETERS OF **5A**

**Bond lengths [Å]**		**Bond angles [°]**	
P1-C1	1.851(2)	C1-P2-O2	88.91(7)
P2-C1	1.821(2)	C1-P2-O3	92.75(7)
P1-B1	1.935(2)	O1-P2-O2	90.77(6)
P1-C2	1.871(2)	O1-P2-O3	87.20(6)
P1-C6	1.873(2)	O2-P2-O4	88.73(6)
P2-O1	1.627(1)	O3-P2-O4	91.73(6)
P2-O2	1.713(1)	C1-P2-O1	130.65(8)
P2-O3	1.708(1)	C1-P2-O4	113.89(8)
P2-O4	1.629(1)	O1-P2-O4	115.44(6)
		P1-C1-P2	121.3(1)

Single crystal X-ray structure analysis of 5b

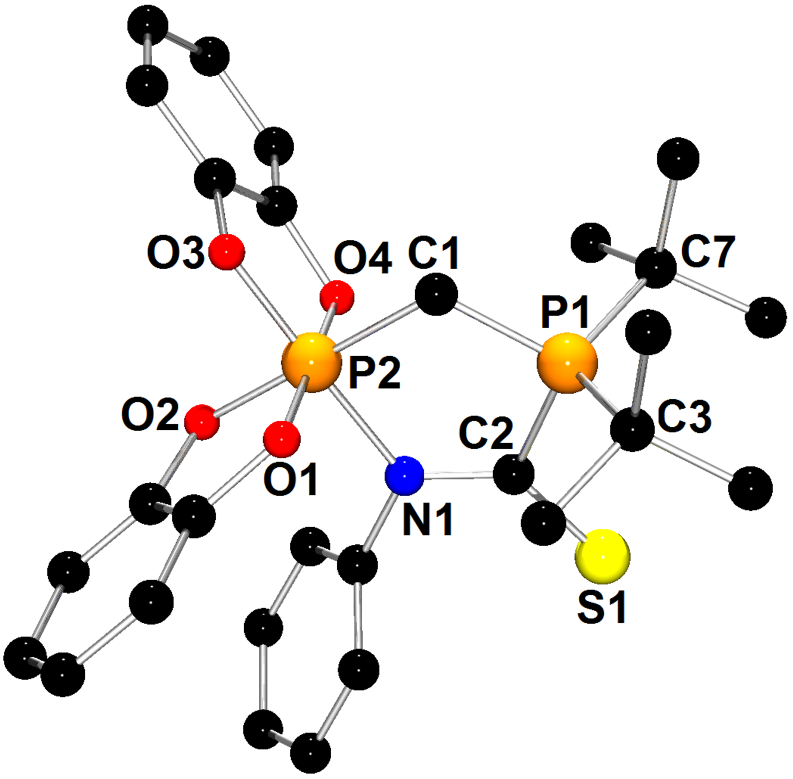

FIG. S10. MOLECULAR STRUCTURE OF **5B**

TABLE S16. SELECTED STRUCTURAL PARAMETERS OF **5B**

**Bond lengths [Å]**		**Bond angles [°]**		**Dihedrals [°]**	
P1-C1	1.788(2)	P1-C1-P2	113.64(8)	P2-C1-P1-C2	-0.8(1)
P2-C1	1.877(2)	C1-P1-C2	99.83(7)	P2-N1-C2-S1	-174.56(9)
P1-C2	1.823(2)	P1-C2-N1	112.7(1)	S1-C2-P1-C1	178.21(9)
P1-C3	1.863(2)	C1-P2-N1	90.80(6)		
P1-C7	1.859(2)	P2-N1-C2	122.5(1)		
P2-O1	1.708(1)	C1-P2-O3	88.96(6)		
P2-O2	1.709(1)	C1-P2-N1	90.80(6)		
P2-O3	1.733(1)	O1-P2-O3	90.65(6)		
P2-O4	1.711(1)	O1-P2-N1	92.11(6)		
P2-N1	1.910(1)	O2-P2-O3	90.85(5)		
N1-C2	1.329(2)	O2-P2-O3	90.85(5)		
S1-C2	1.662(2)	O2-P2-N1	89.41(5)		
		O3-P2-O4	91.54(5)		
		O4-P2-N1	85.71(5)		
		C1-P2-O1	89.69(6)		
		C1-P2-O4	93.33(6)		
		O1-P2-O2	89.94(6)		
		O2-P2-O4	87.05(6)		

Single crystal X-ray structure analysis of 6

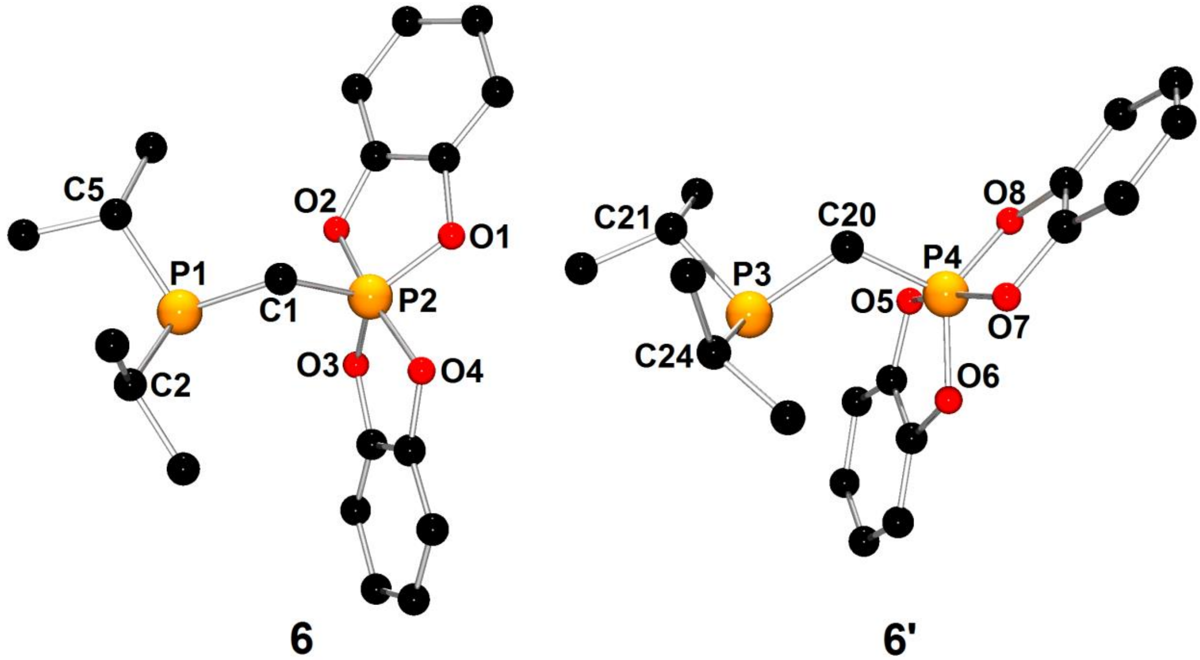

6 6'

FIG. S11. MOLECULAR STRUCTURE OF **6** AND **6'**

TABLE S17. SELECTED STRUCTURAL PARAMETERS OF **6**

**Bond lengths [Å]**		**Bond angles [°]**	
P1-C1	1.878(5)	P1-C1-P2	112.1(3)
P2-C1	1.800(6)	C1-P2-O2	94.2(2)
P1-C2	1.867(6)	C1-P2-O4	96.7(2)
P1-C5	1.853(6)	O2-P2-O3	84.5(2)
P2-O1	1.644(4)	O3-P2-O4	90.7(2)
P2-O2	1.712(4)	O1-P2-O2	90.9(2)
P2-O3	1.639(4)	O1-P2-O4	85.0(2)
P2-O4	1.710(4)	C1-P2-O1	112.8(2)
		C1-P2-O3	115.8(2)
		O1-P2-O3	131.4(2)

TABLE S18. SELECTED STRUCTURAL PARAMETERS OF **6'**

**Bond lengths [Å]**		**Bond angles [°]**	
P3-C20	1.863(6)	P3-C20-P4	113.7(3)
P4-C20	1.798(5)	C20-P4-O5	95.4(2)
P3-C21	1.851(5)	C20-P4-O7	97.9(2)
P3-C24	1.856(6)	O5-P4-O6	90.6(2)
P4-O6	1.647(4)	O6-P4-O7	84.3(2)
P4-O7	1.706(4)	O5-P4-O8	84.5(2)
P4-O8	1.654(4)	O7-P4-O8	90.5(2)
P4-O5	1.698(4)	C20-P4-O6	113.2(2)
		C20-P4-O8	111.8(2)
		O6-P4-O8	135.0(2)

Single crystal X-ray structure analysis of 6a

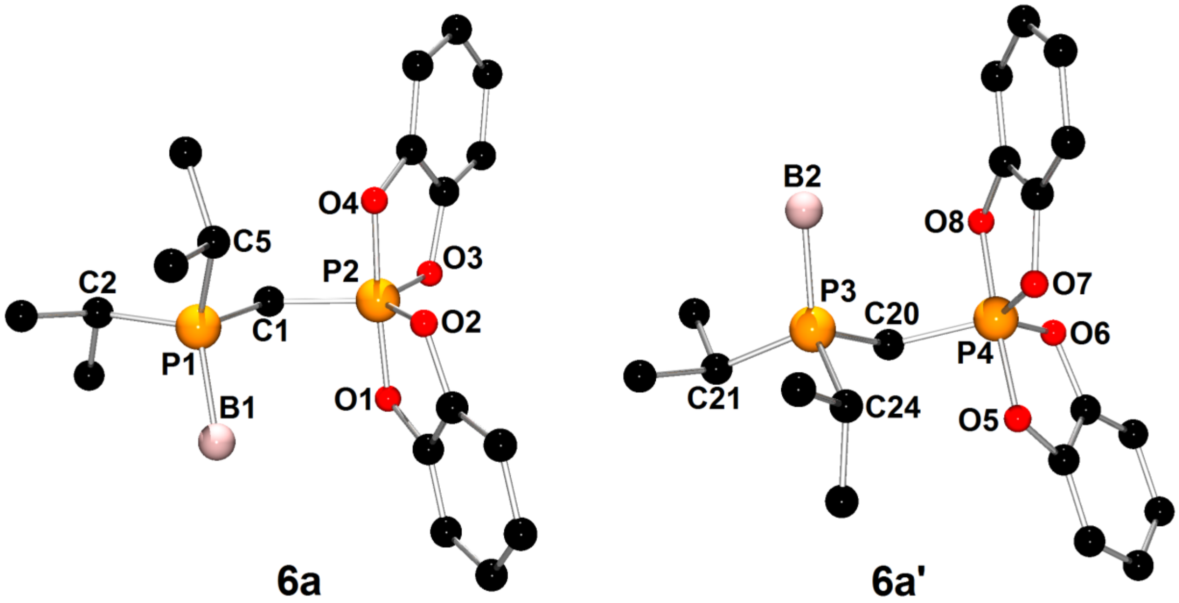

FIG. S12. MOLECULAR STRUCTURE OF **6A** AND **6A'**

TABLE S19. SELECTED STRUCTURAL PARAMETERS OF **6A**

Bond lengths [Å]		Bond angles [°]	
P1-C1	1.841(3)	P1-C1-P2	116.8(2)
P2-C1	1.809(3)	C1-P2-O1	92.4(1)
P1-B1	1.918(5)	C1-P2-O4	91.5(1)
P1-C2	1.837(3)	O1-P2-O2	90.5(1)
P1-C5	1.836(3)	O1-P2-O3	88.3(1)
P2-O1	1.714(2)	O2-P2-O4	85.9(1)
P2-O2	1.630(2)	O3-P2-O4	91.6(1)
P2-O3	1.619(2)	C1-P2-O2	124.1(1)
P2-O4	1.707(2)	C1-P2-O3	114.8(1)
		O2-P2-O3	121.1(1)

TABLE S20. SELECTED STRUCTURAL PARAMETERS OF **6A'**

Bond lengths [Å]		Bond angles [°]	
P3-C20	1.834(3)	P3-C20-P4	117.0(2)
P4-C20	1.806(3)	C20-P4-O8	92.0(1)
P3-B2	1.919(4)	C20-P4-O5	91.5(1)
P3-C21	1.829(3)	O5-P4-O7	86.3(1)
P3-C24	1.837(3)	O7-P4-O8	91.3(1)
P4-O5	1.723(2)	O6-P4-O8	87.7(1)
P4-O6	1.619(2)	O6-P4-O5	91.4(1)
P4-O7	1.624(2)	C20-P4-O6	115.2(1)
P4-O8	1.703(2)	C20-P4-O7	123.2(1)
		O6-P4-O7	121.6(1)

Single crystal X-ray structure analysis of 7

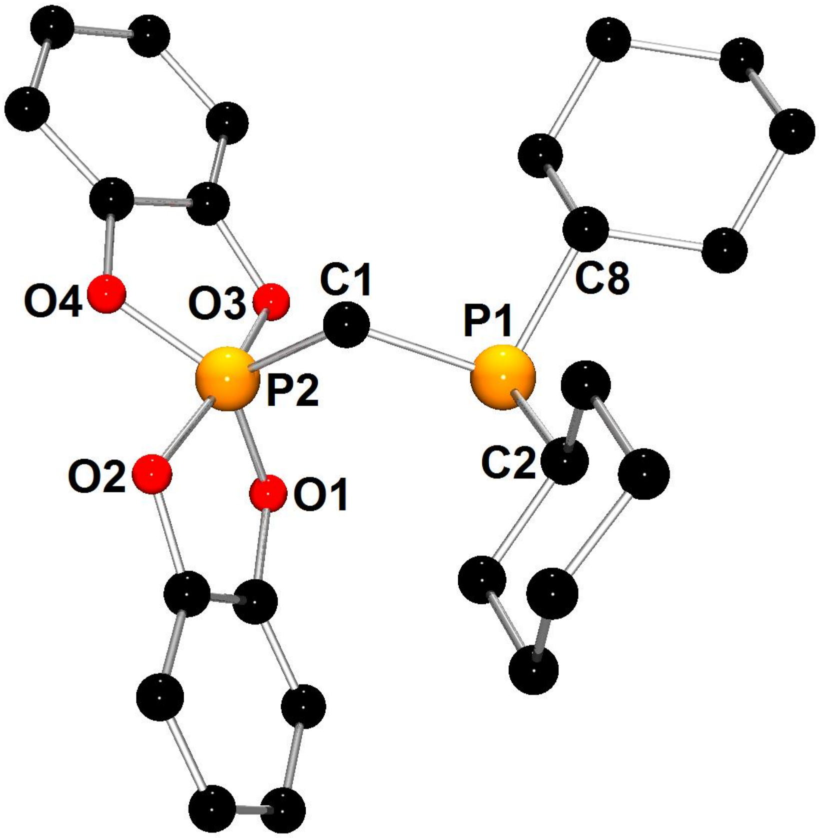

FIG. S13. MOLECULAR STRUCTURE OF **7**

TABLE S21. SELECTED STRUCTURAL PARAMETERS OF **7**

**Bond lengths [Å]**		**Bond angles [°]**	
P1-C1	1.885(5)	P1-C1-P2	114.1(3)
P2-C1	1.794(5)	C1-P2-O3	96.2(2)
P1-C2	1.875(5)	C1-P2-O2	98.3(2)
P1-C8	1.861(5)	O3-P2-O4	90.4(2)
P2-O1	1.650(4)	O2-P2-O4	84.5(2)
P2-O2	1.709(4)	O1-P2-O2	90.0(2)
P2-O3	1.695(4)	O1-P2-O3	84.4(2)
P2-O4	1.642(4)	C1-P2-O1	113.2(2)
		C1-P2-O4	110.1(2)
		O1-P2-O4	136.7(2)

Spectroscopic data

NMR spectra of isolated compounds

NMR spectra of 1

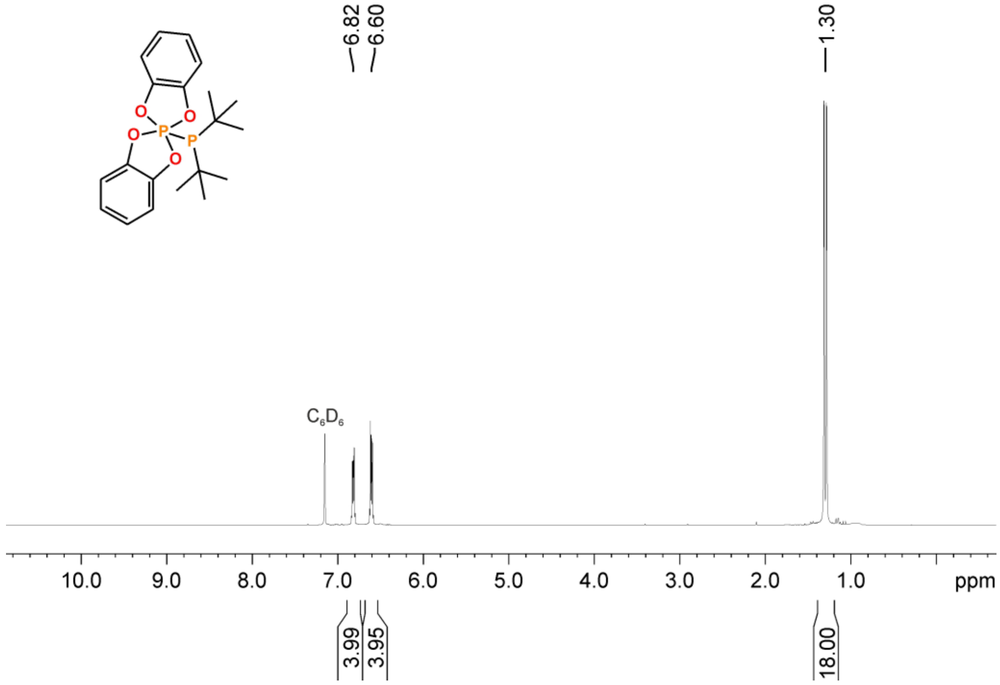

FIG. S14. ^{1}H NMR (C_6D_6) SPECTRUM OF **1**

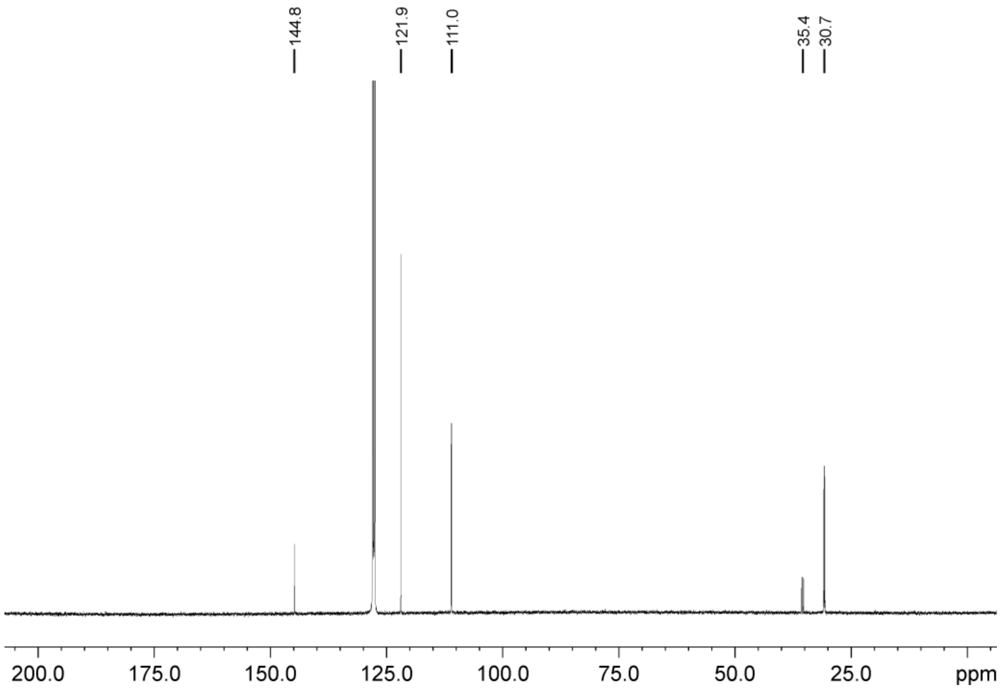

FIG. S15. $^{13}C\{^{1}H\}$ NMR (C_6D_6) SPECTRUM OF **1**

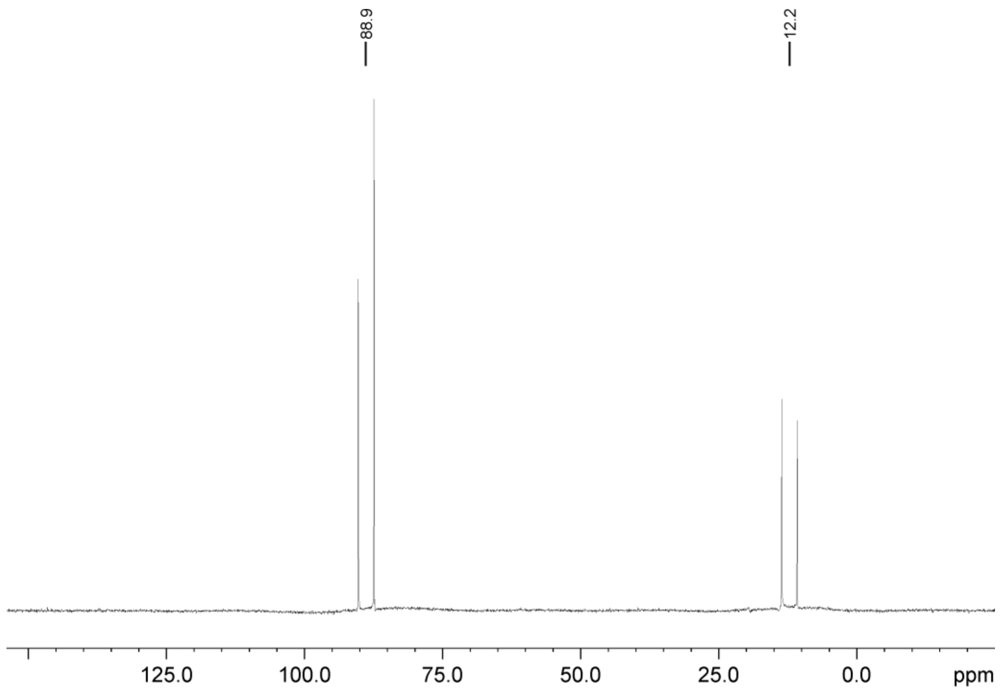

FIG. S16. $^{31}P\{^{1}H\}$ NMR (C_6D_6) SPECTRUM OF **1**

NMR spectra of 1a

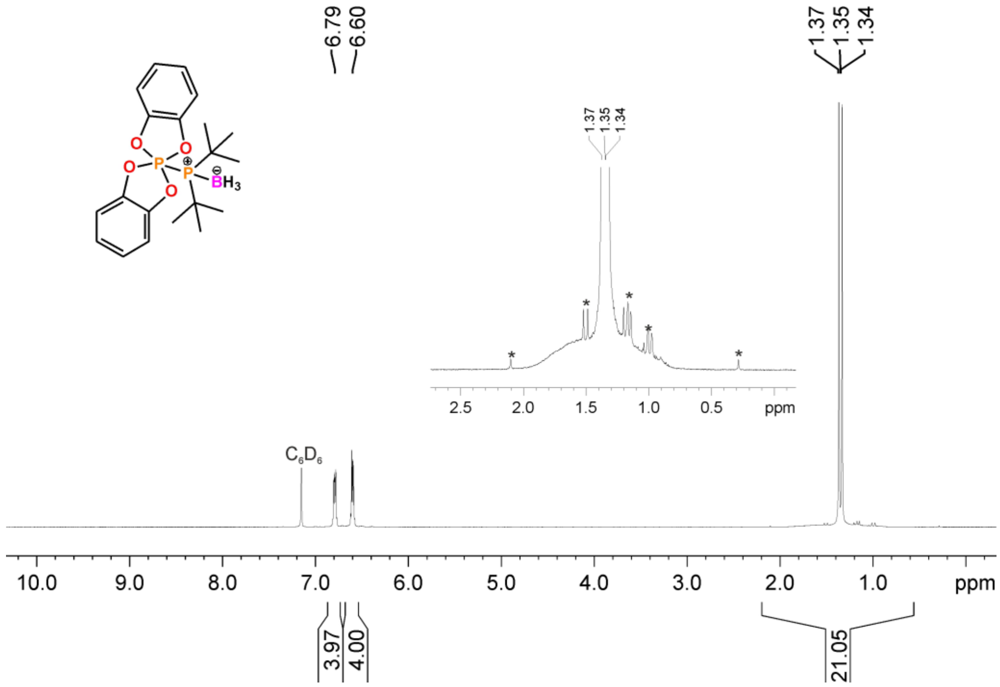

FIG. S17. ^{1}H NMR (C_6D_6) SPECTRUM OF **1A**

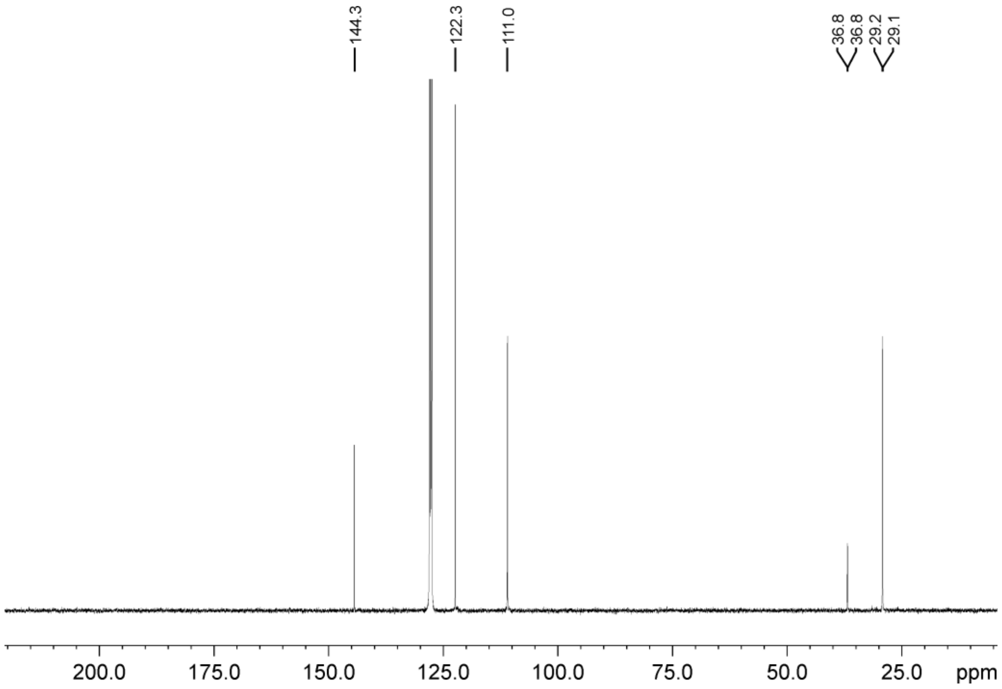

FIG. S18. $^{13}C\{^{1}H\}$ NMR (C_6D_6) SPECTRUM OF **1A**

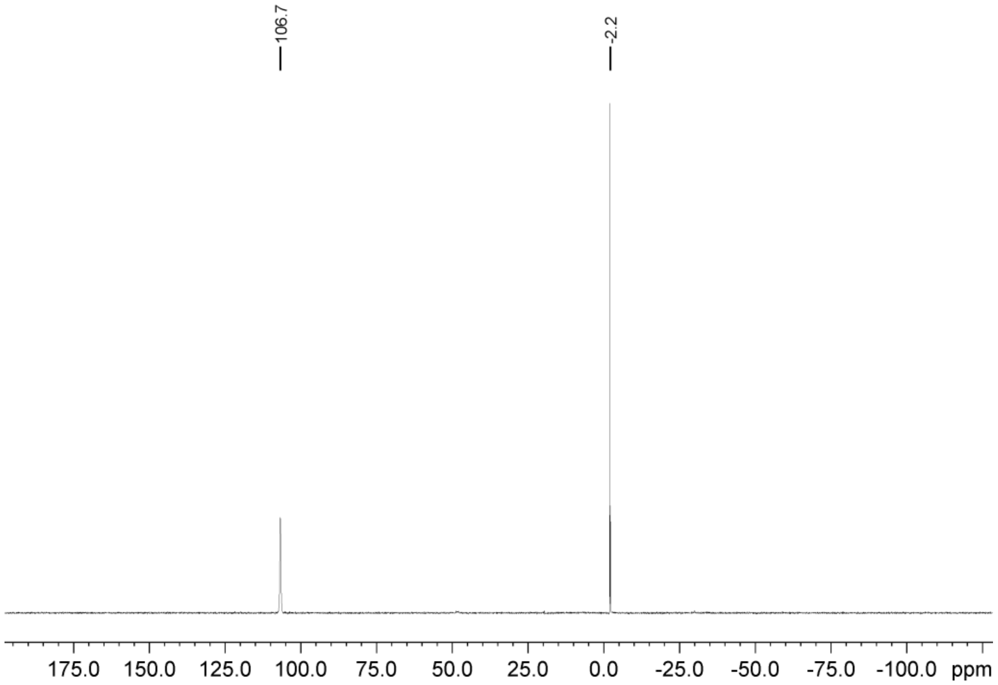

FIG. S19. $^{31}P\{^{1}H\}$ NMR (C_6D_6) SPECTRUM OF **1A**

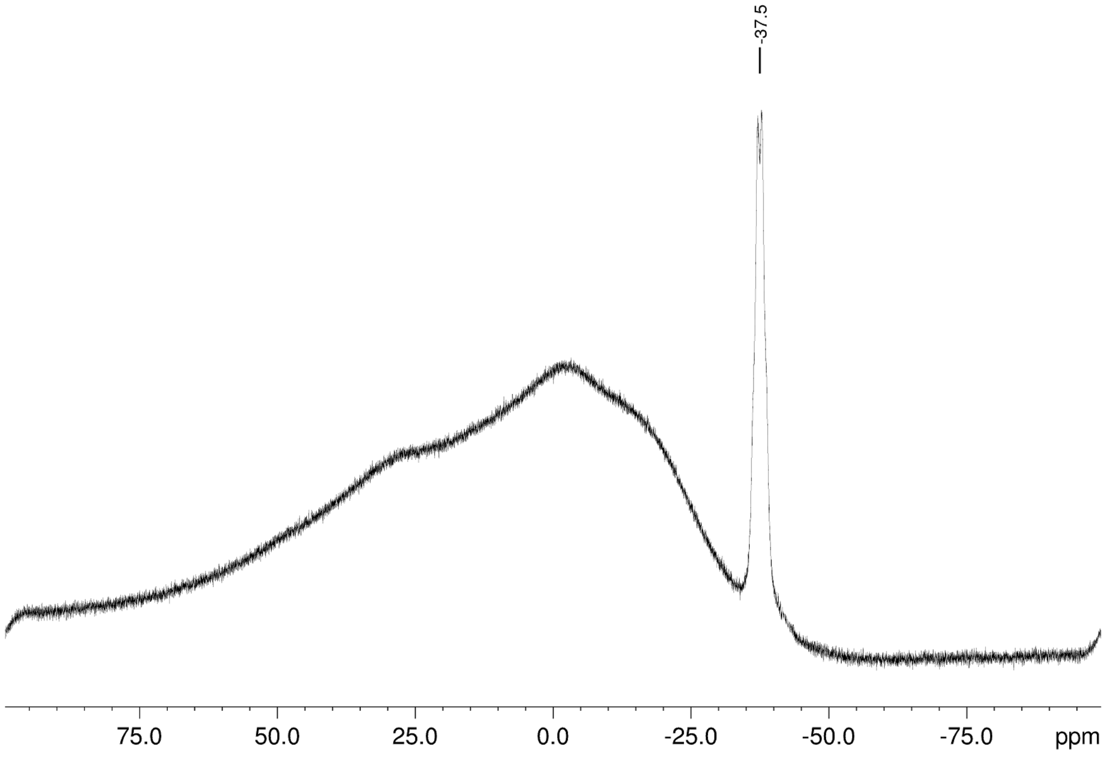

FIG. S20. ^{11}B NMR (C_6D_6) SPECTRUM OF **1A**

NMR spectra of 1b

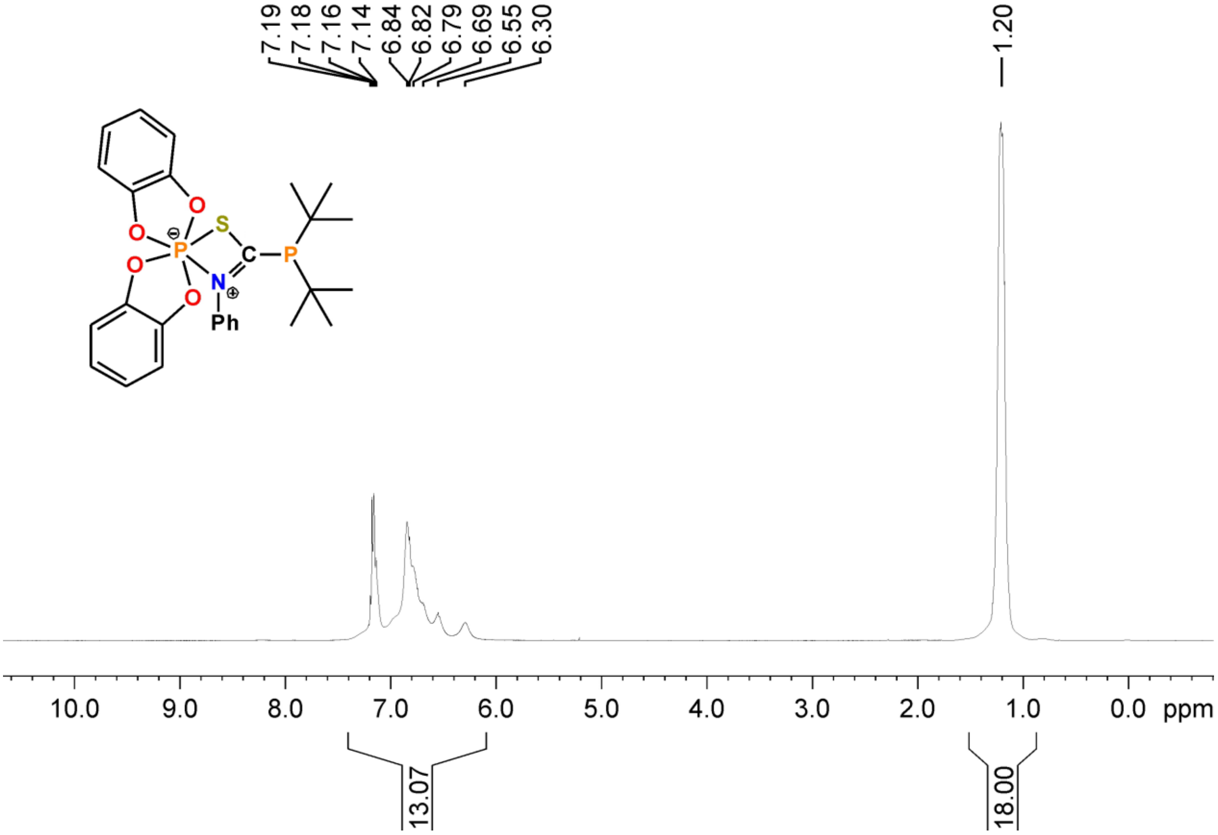

FIG. S21. ^{1}H NMR ($CDCl_3$) SPECTRUM OF **1B**

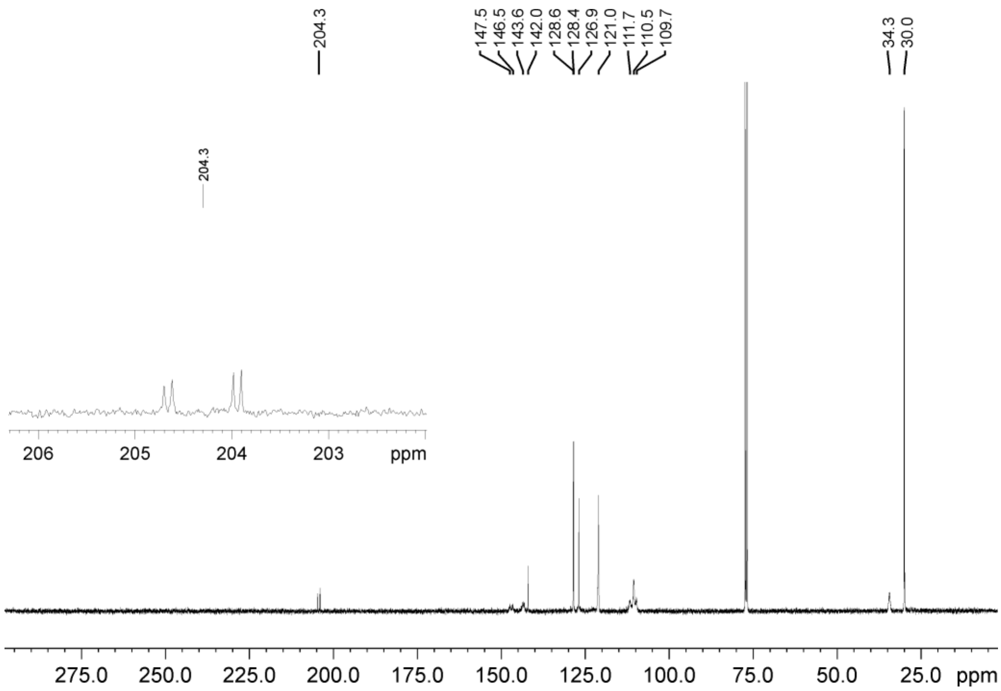

FIG. S22. $^{13}C\{^1H\}$ NMR ($CDCl_3$) SPECTRUM OF **1B**

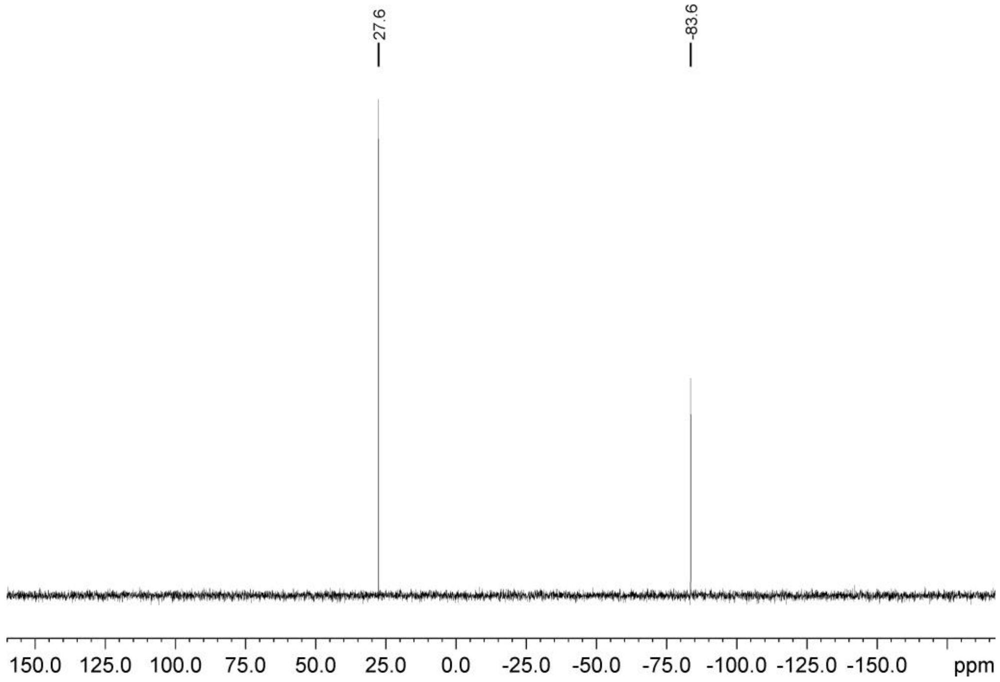

FIG. S23. $^{31}P\{^1H\}$ NMR ($CDCl_3$) SPECTRUM OF **1B**

NMR spectra of 2

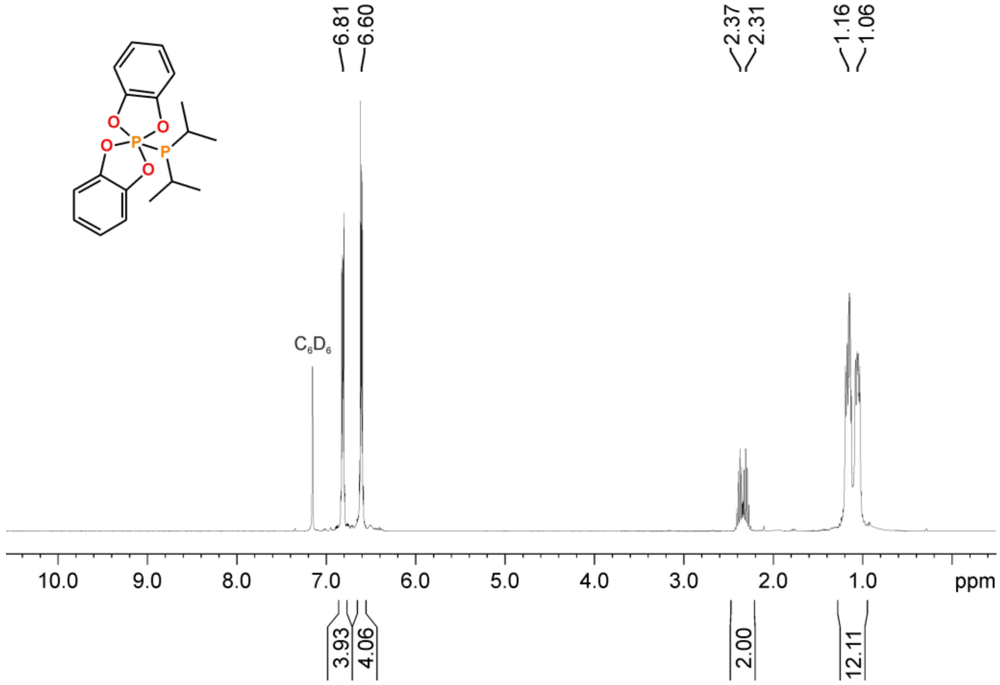

FIG. S24. ^{1}H NMR (C_6D_6) SPECTRUM OF **2**

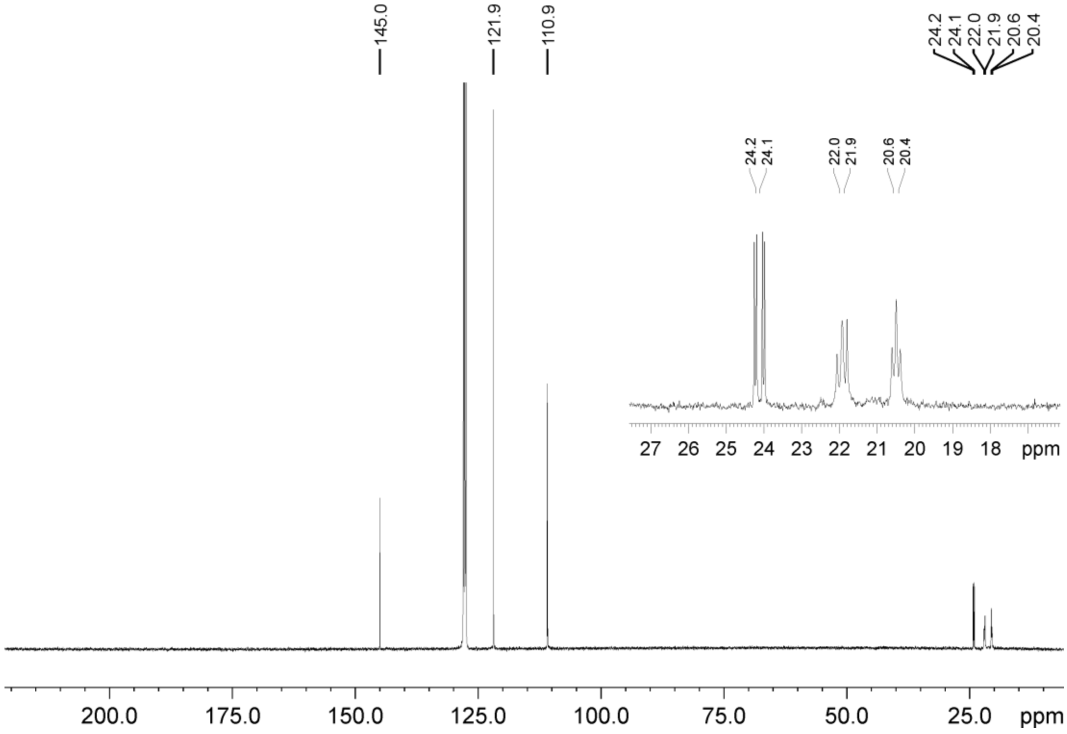

FIG. S25. $^{13}C\{^{1}H\}$ NMR (C_6D_6) SPECTRUM OF **2**

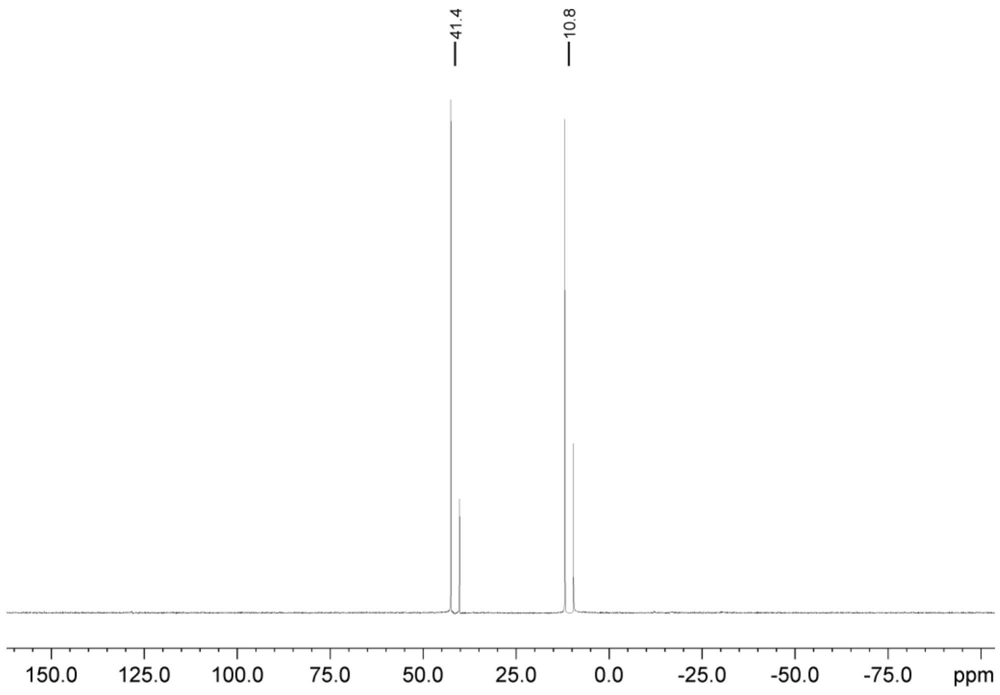

FIG. S26. $^{31}P\{^{1}H\}$ NMR (C_6D_6) SPECTRUM OF **2**

NMR spectra of 2a

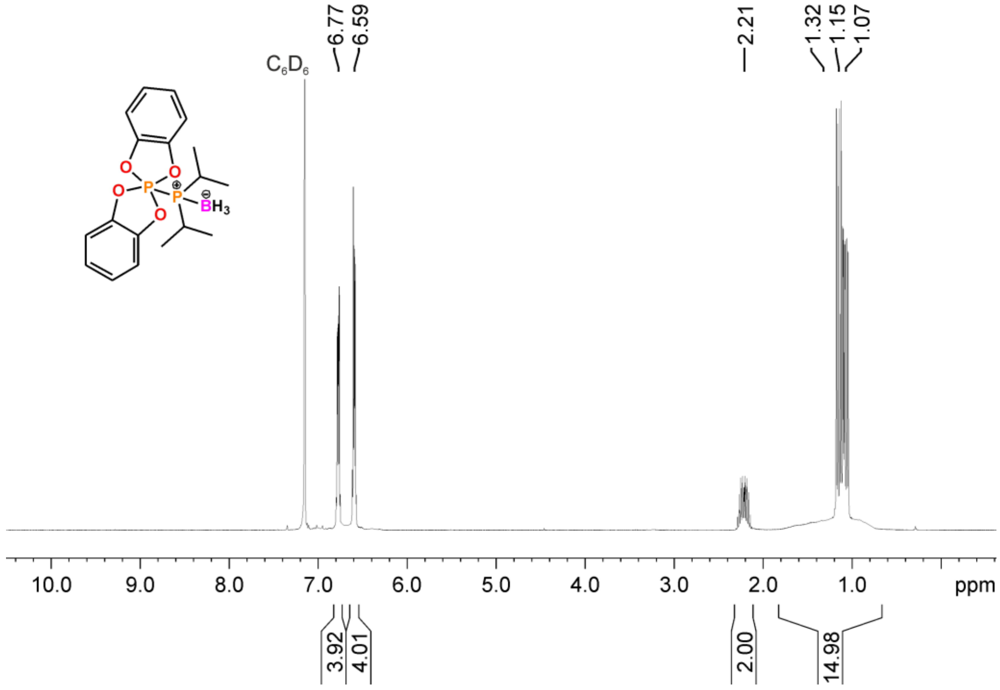

FIG. S27. ^{1}H NMR (C_6D_6) SPECTRUM OF **2A**

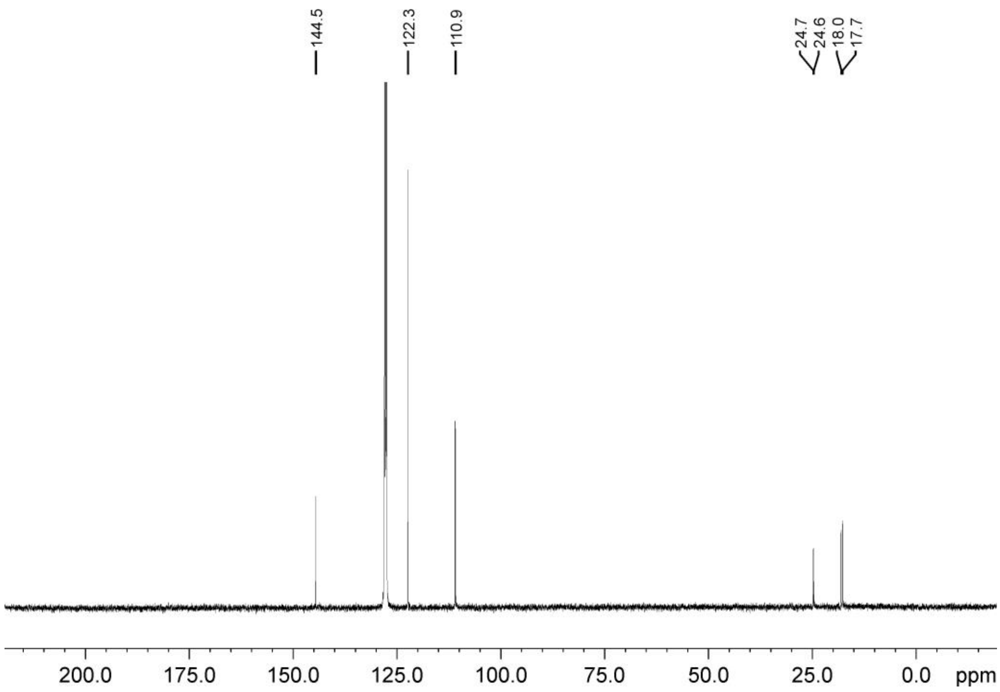

FIG. S28. $^{13}C\{^{1}H\}$ NMR (C_6D_6) SPECTRUM OF **2A**

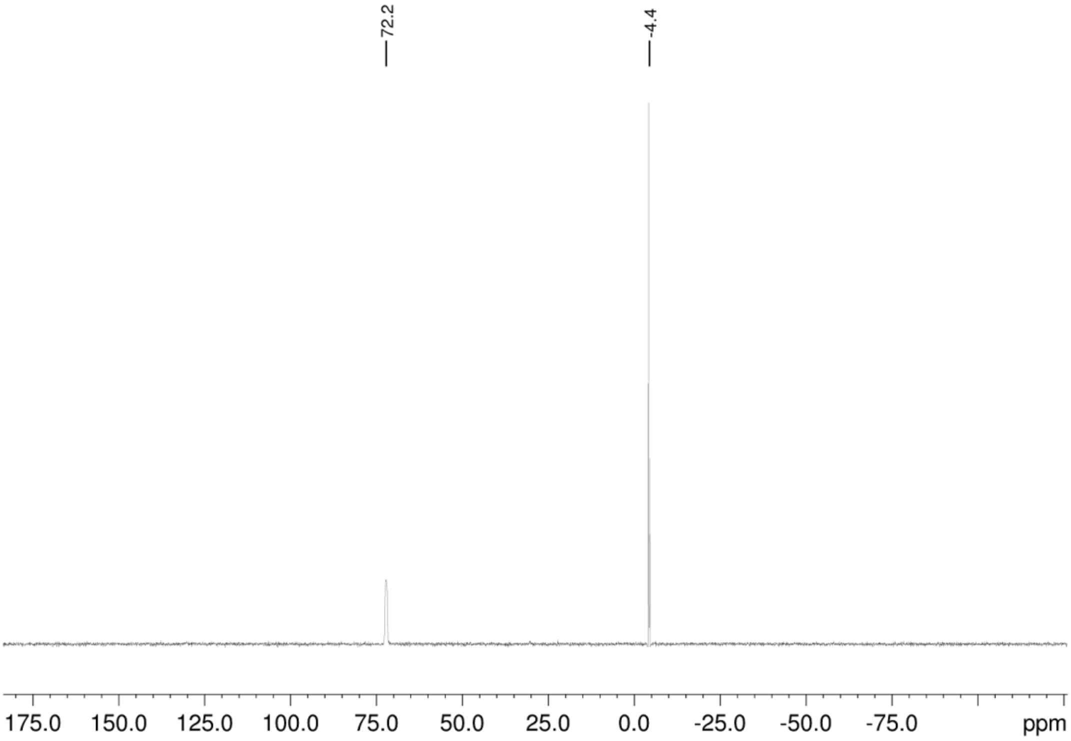

FIG. S29. $^{31}P\{^{1}H\}$ NMR (C_6D_6) SPECTRUM OF **2A**

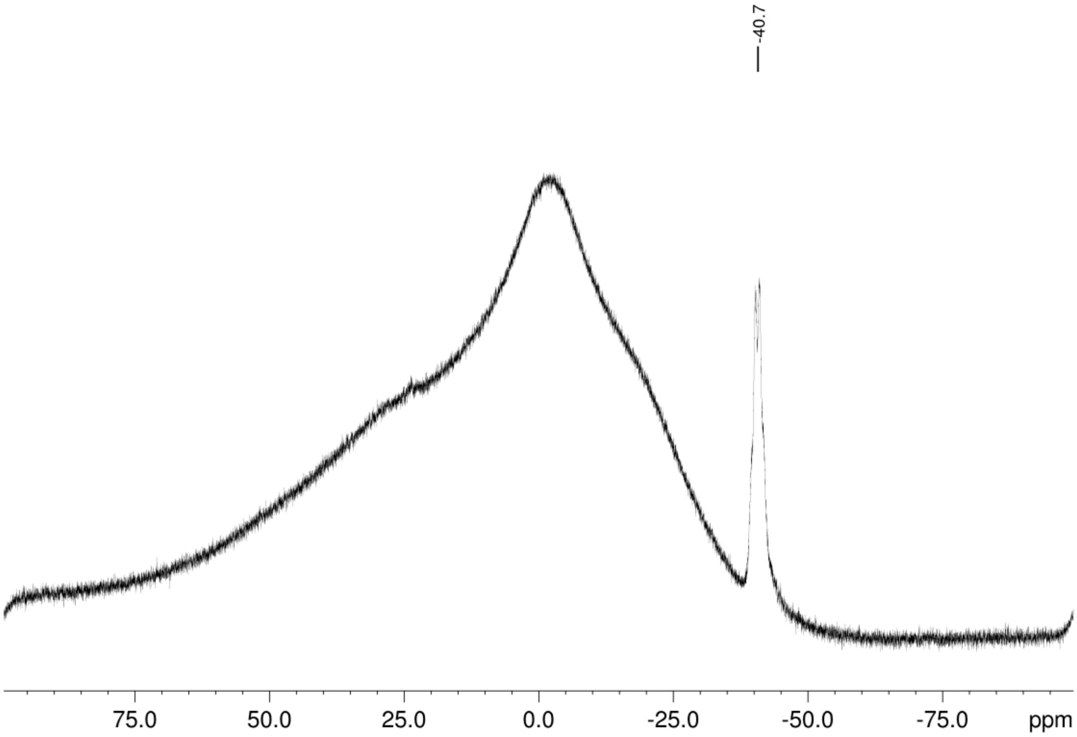

FIG. S30. ^{11}B NMR (C_6D_6) SPECTRUM OF **2A**

NMR spectra of 3

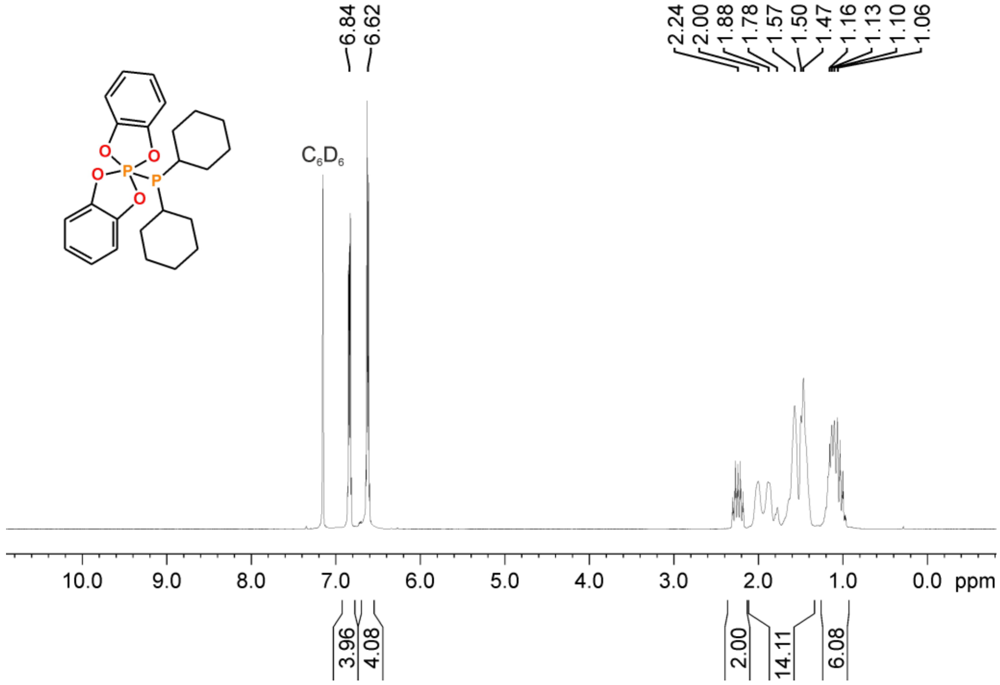

FIG. S31. ^{1}H NMR (C_6D_6) SPECTRUM OF **3**

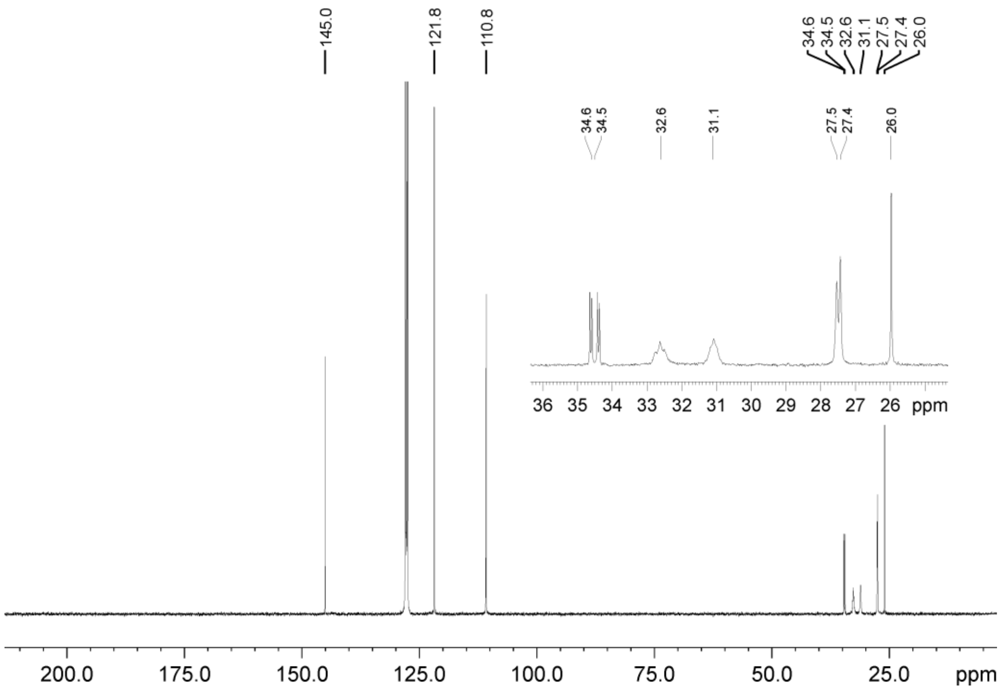

FIG. S32. $^{13}C\{^{1}H\}$ NMR (C_6D_6) SPECTRUM OF **3**

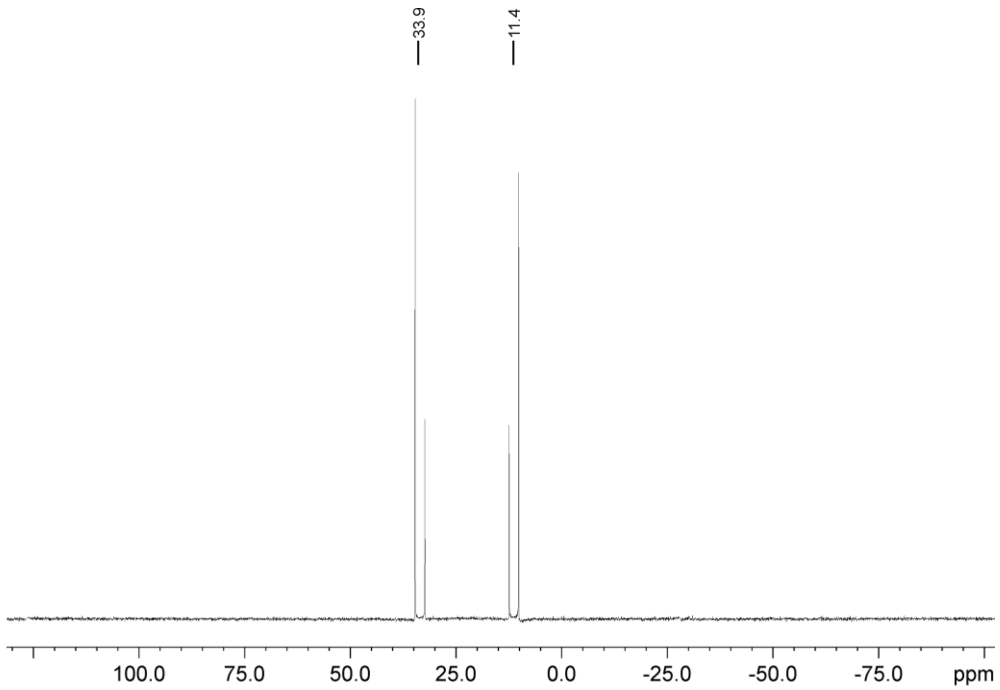

FIG. S33. $^{31}P\{^{1}H\}$ NMR (C_6D_6) SPECTRUM OF **3**

NMR spectra of 3a

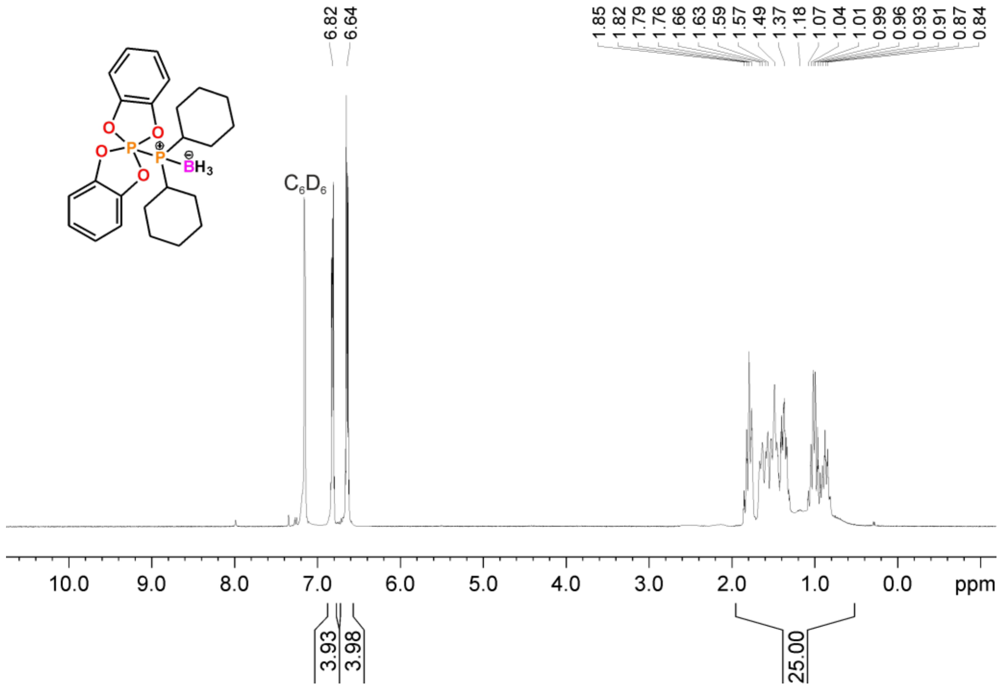

FIG. S34. ^{1}H NMR (C_6D_6) SPECTRUM OF **3A**

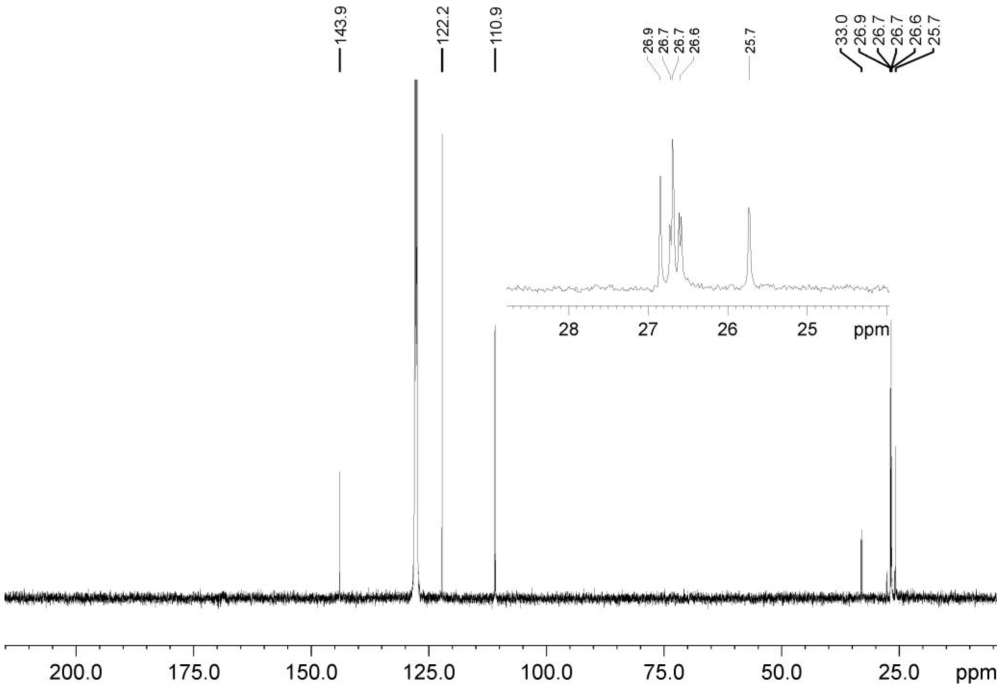

FIG. S35. ^{13}C{^{1}H} NMR (C_6D_6) SPECTRUM OF **3A**

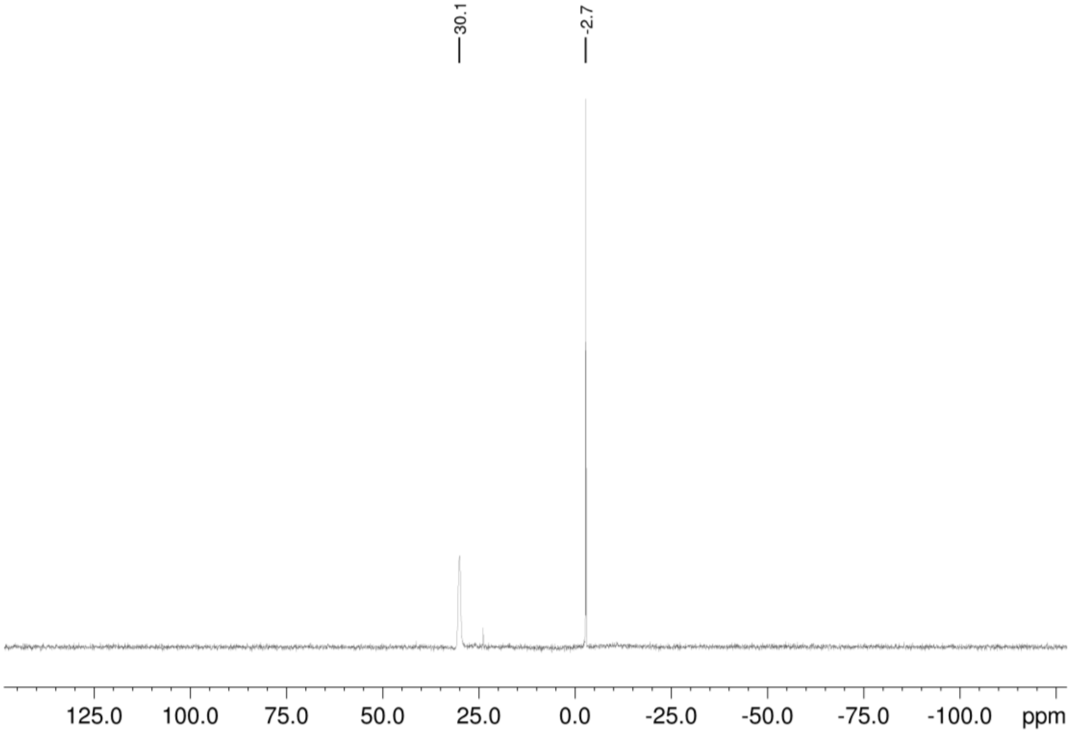

FIG. S36. $^{31}P\{^{1}H\}$ NMR (C_6D_6) SPECTRUM OF **3A**

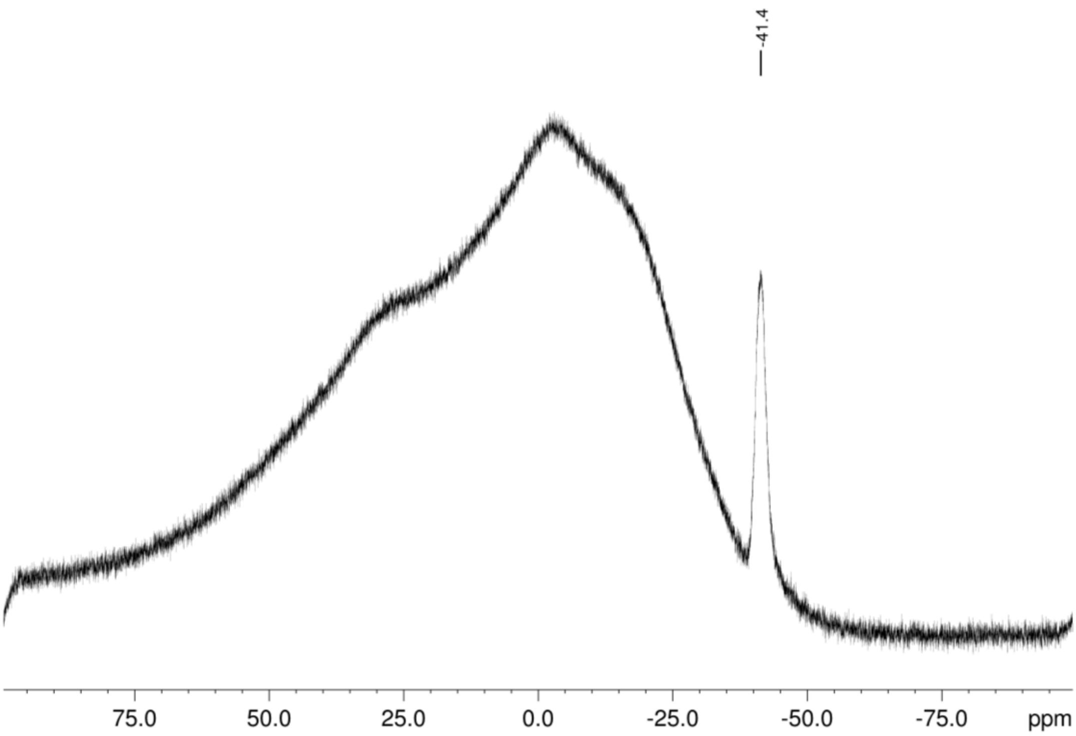

FIG. S37. ^{11}B NMR (C_6D_6) SPECTRUM OF **3A**

NMR spectra of 4

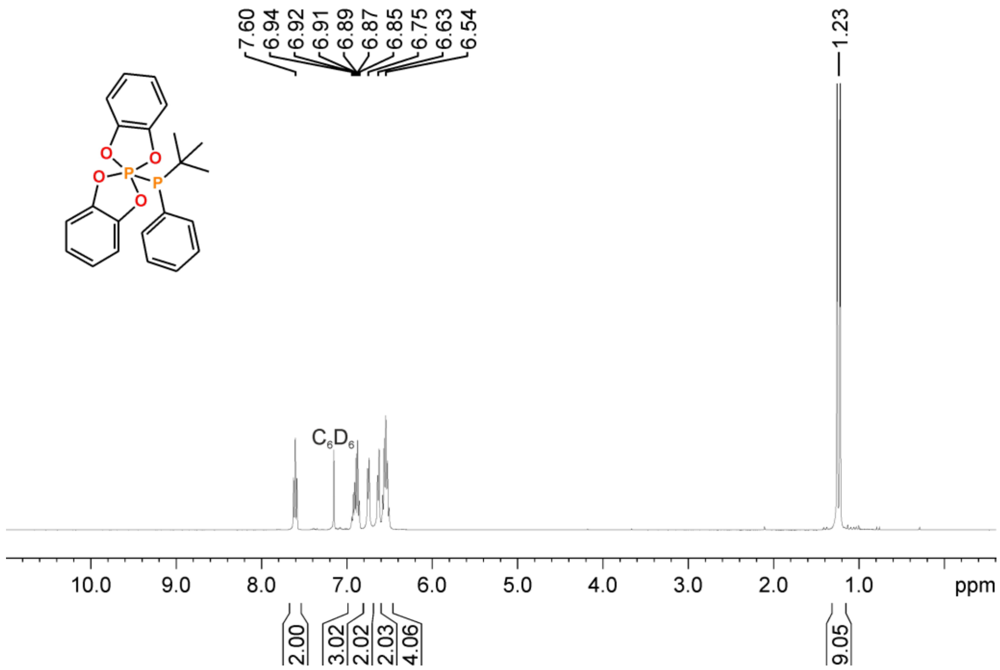

FIG. S38. ^{1}H NMR (C_6D_6) SPECTRUM OF **4**

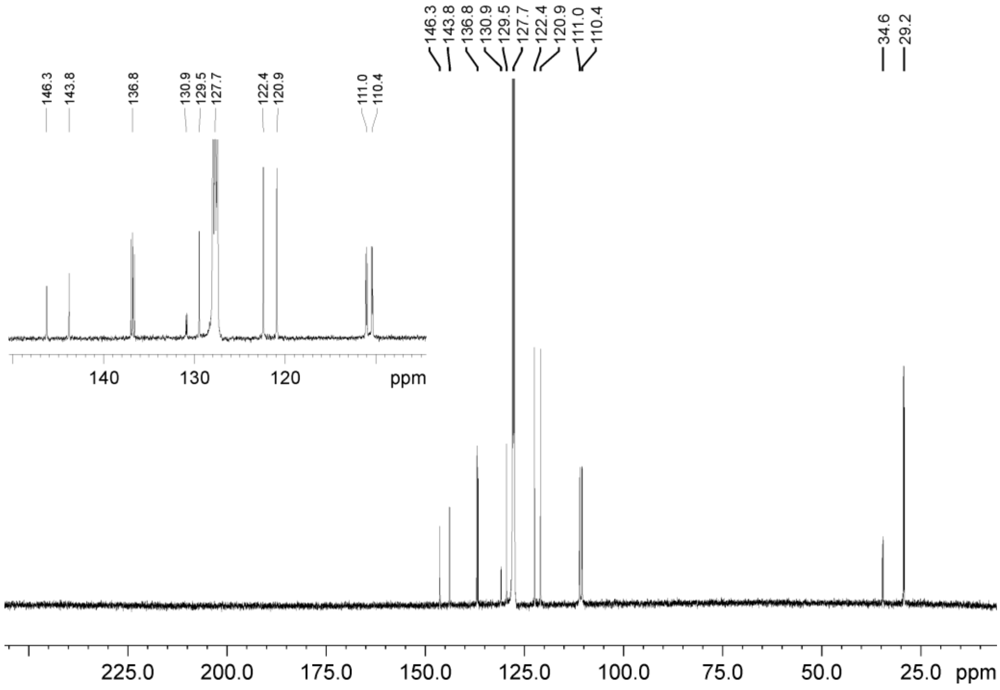

FIG. S39. $^{13}C\{^{1}H\}$ NMR (C_6D_6) SPECTRUM OF **4**

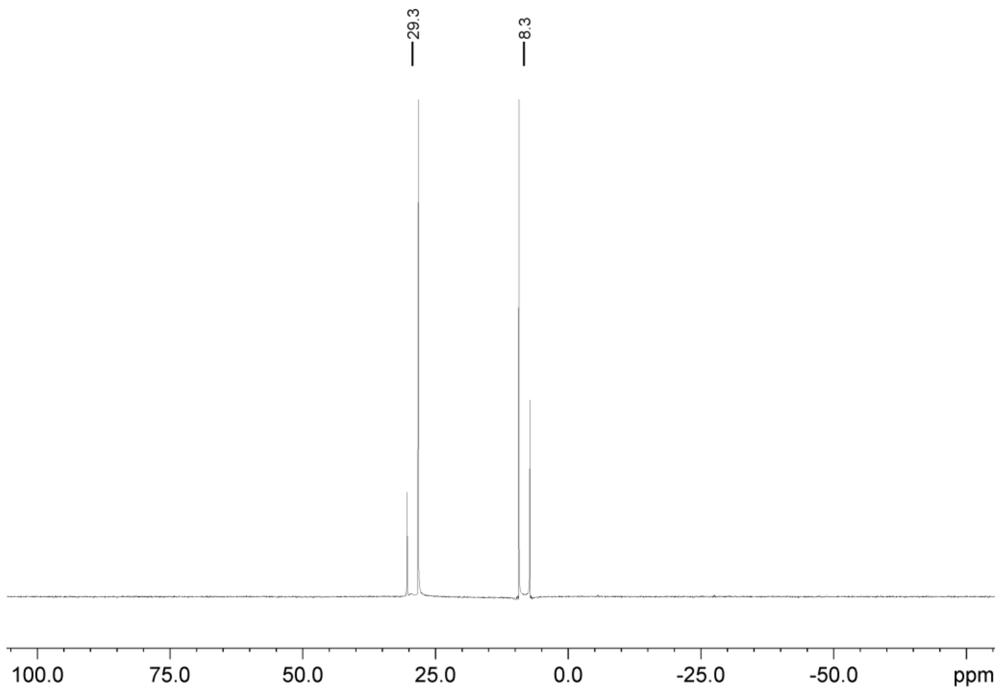

FIG. S40. $^{31}P\{^{1}H\}$ NMR (C_6D_6) SPECTRUM OF **4**

NMR spectra of 4a

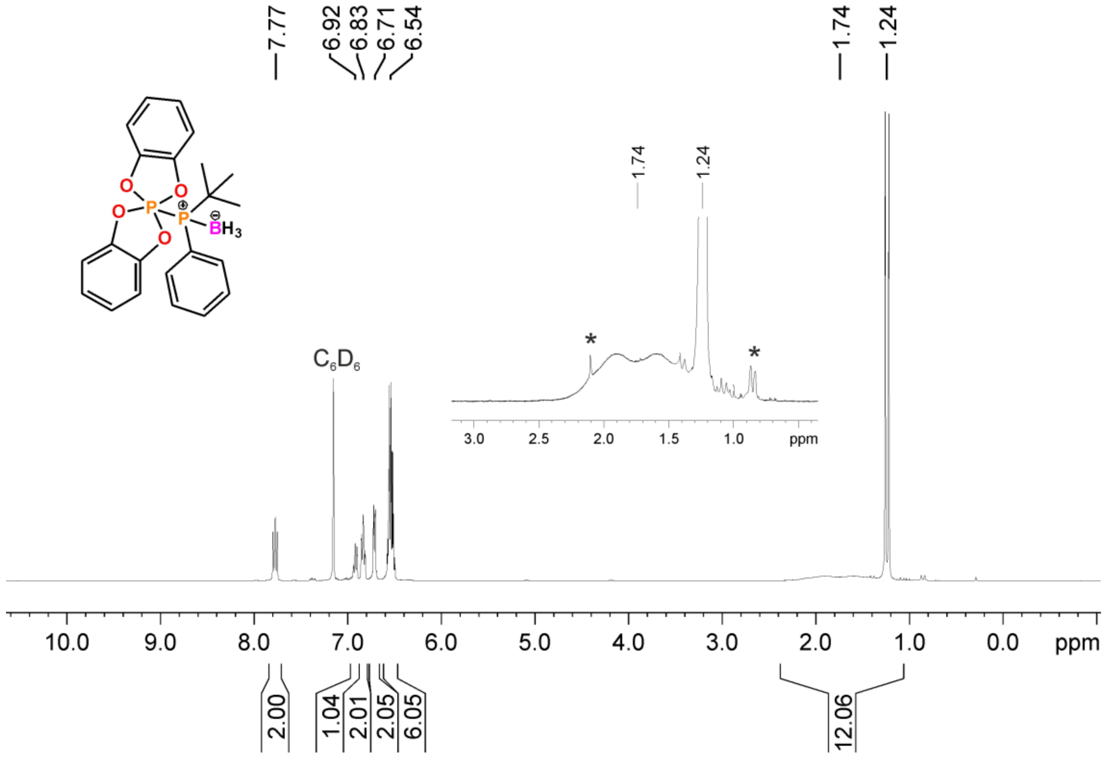

FIG. S41. ^{1}H NMR (C_6D_6) SPECTRUM OF **4A**

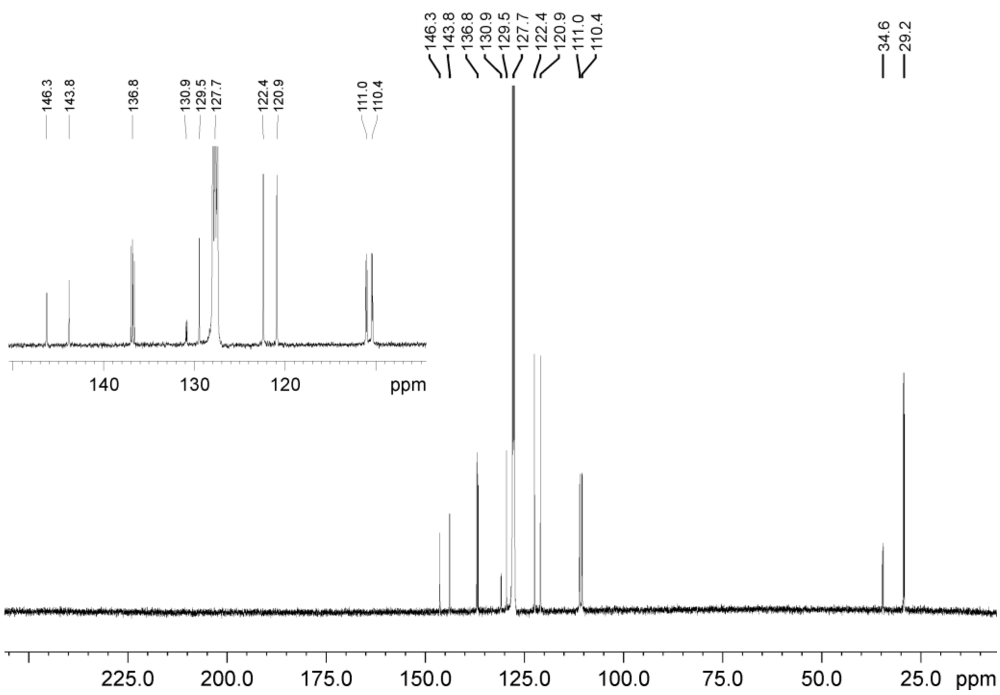

FIG. S42. $^{13}C\{^{1}H\}$ NMR (C_6D_6) SPECTRUM OF **4A**

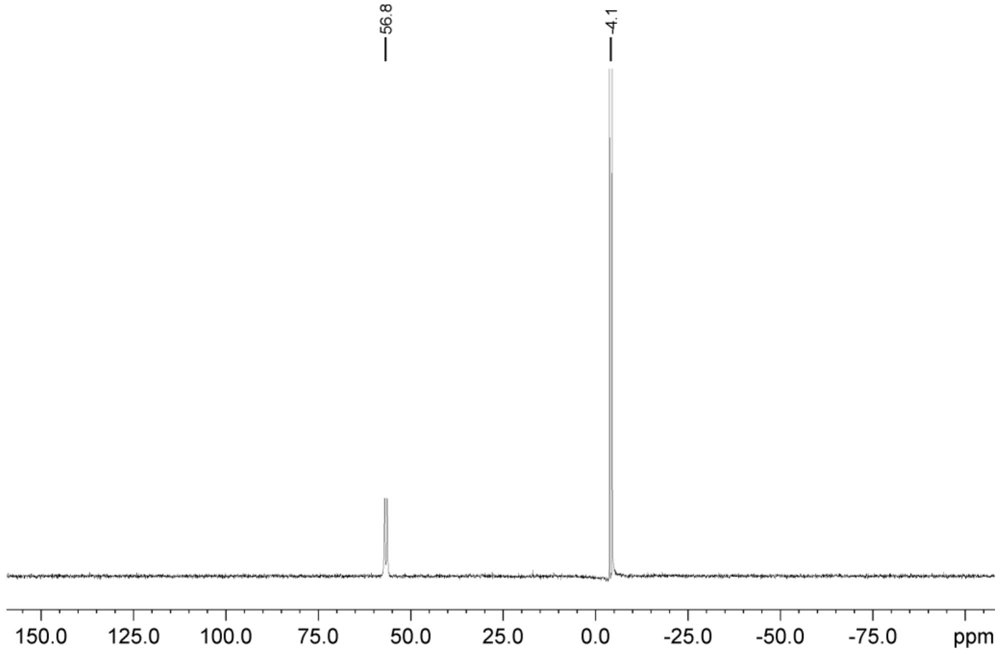

FIG. S43. $^{31}P\{^{1}H\}$ NMR (C_6D_6) SPECTRUM OF **4A**

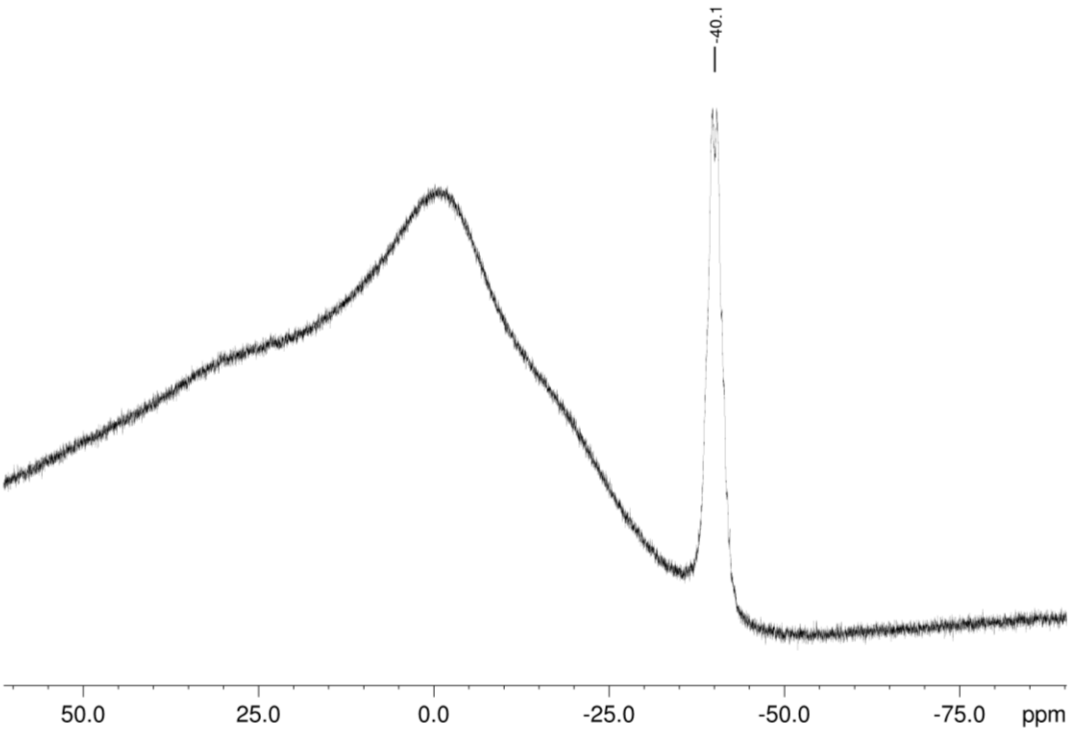

FIG. S44. ^{11}B NMR (C_6D_6) SPECTRUM OF **4A**

NMR spectra of 5

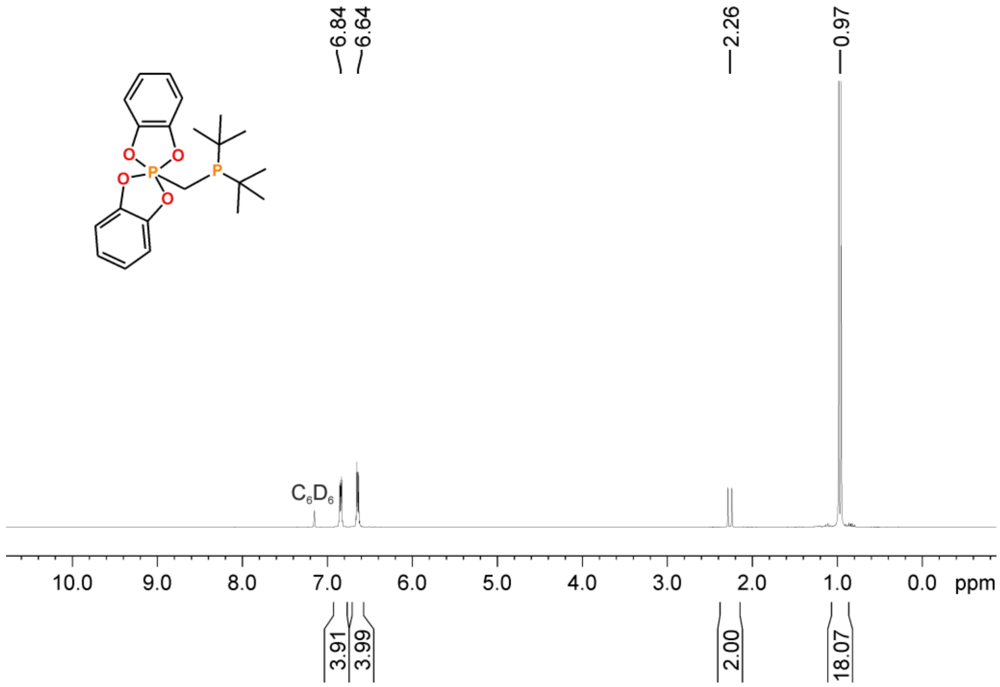

FIG. S45. ^{1}H NMR (C_6D_6) SPECTRUM OF **5**

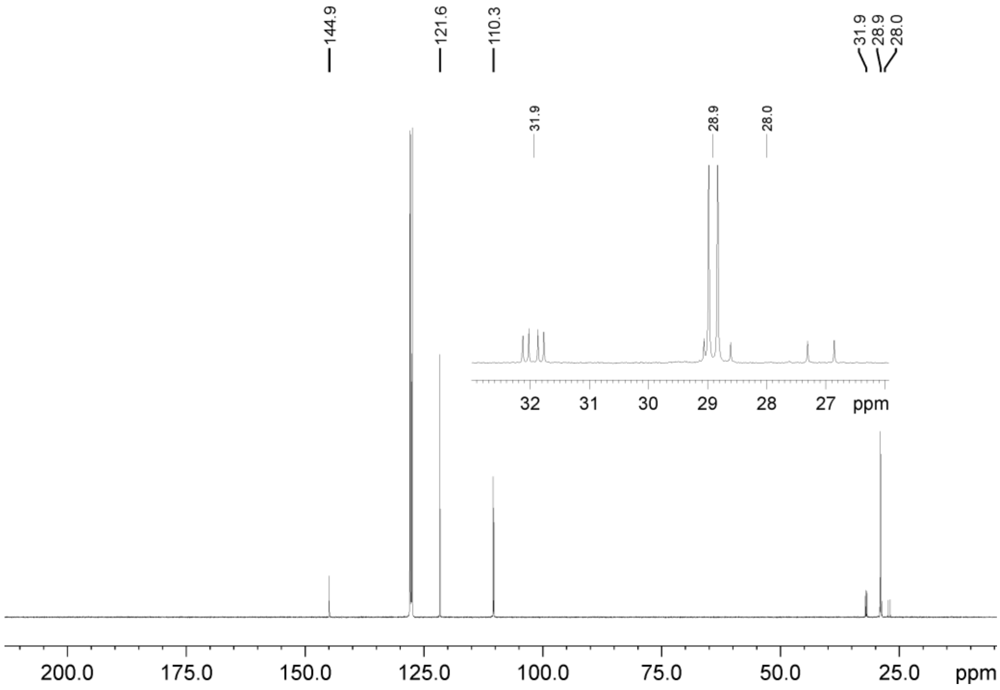

FIG. S46. $^{13}C\{^{1}H\}$ NMR (C_6D_6) SPECTRUM OF **5**

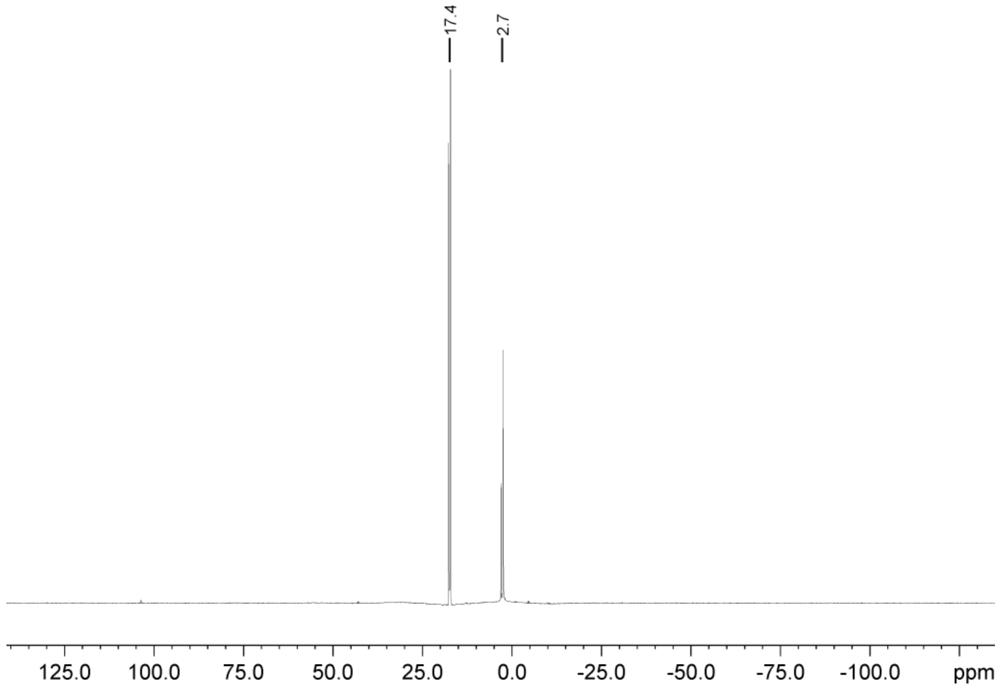

FIG. S47. $^{31}P\{^{1}H\}$ NMR (C_6D_6) SPECTRUM OF **5**

NMR spectra of 5a

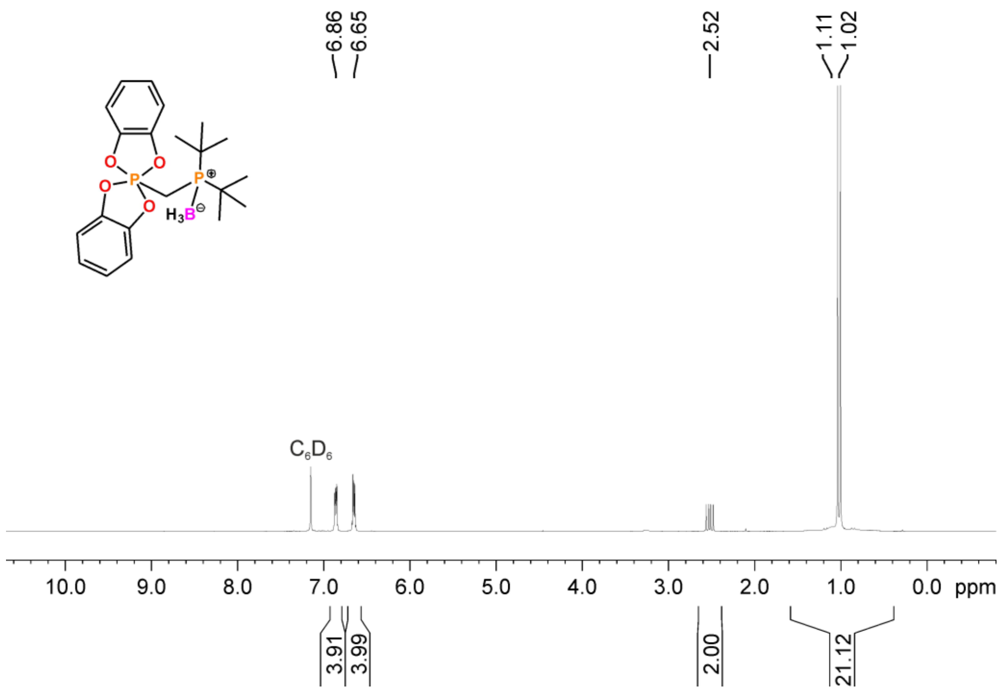

FIG. S48. ^{1}H NMR (C_6D_6) SPECTRUM OF **5A**

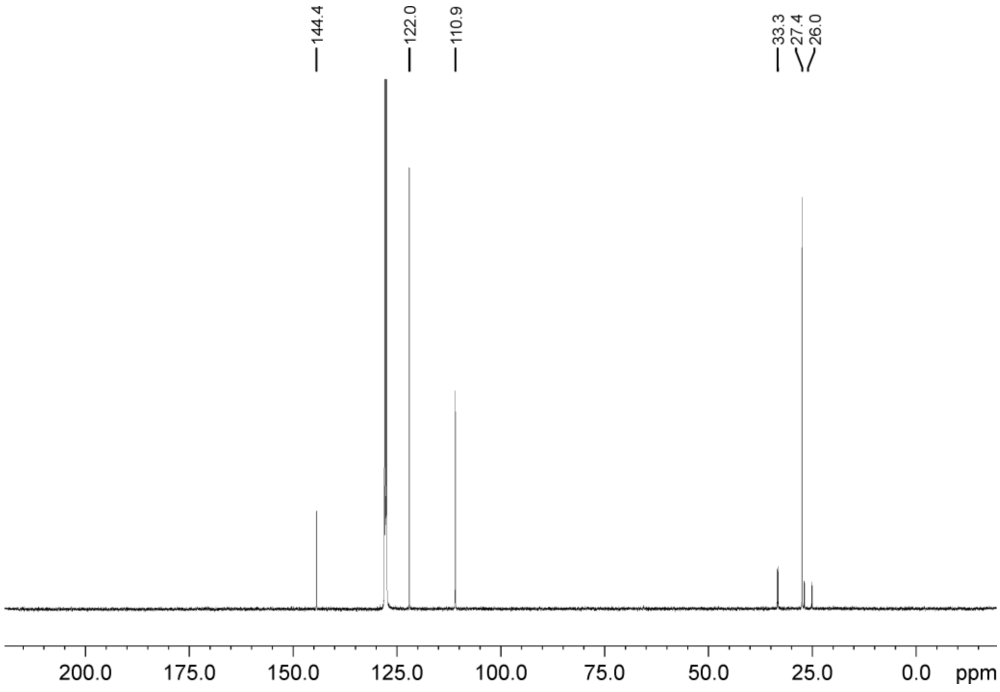

FIG. S49. $^{13}C\{^{1}H\}$ NMR (C_6D_6) SPECTRUM OF **5A**

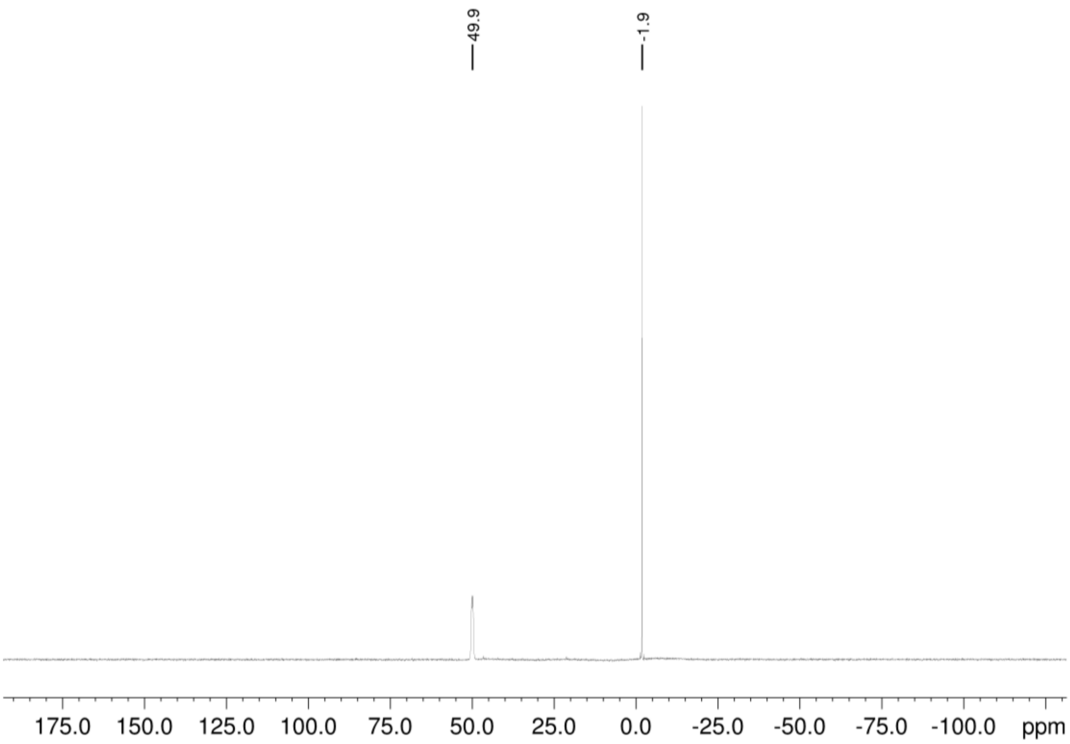

FIG. S50. $^{31}P\{^{1}H\}$ NMR (C_6D_6) SPECTRUM OF **5A**

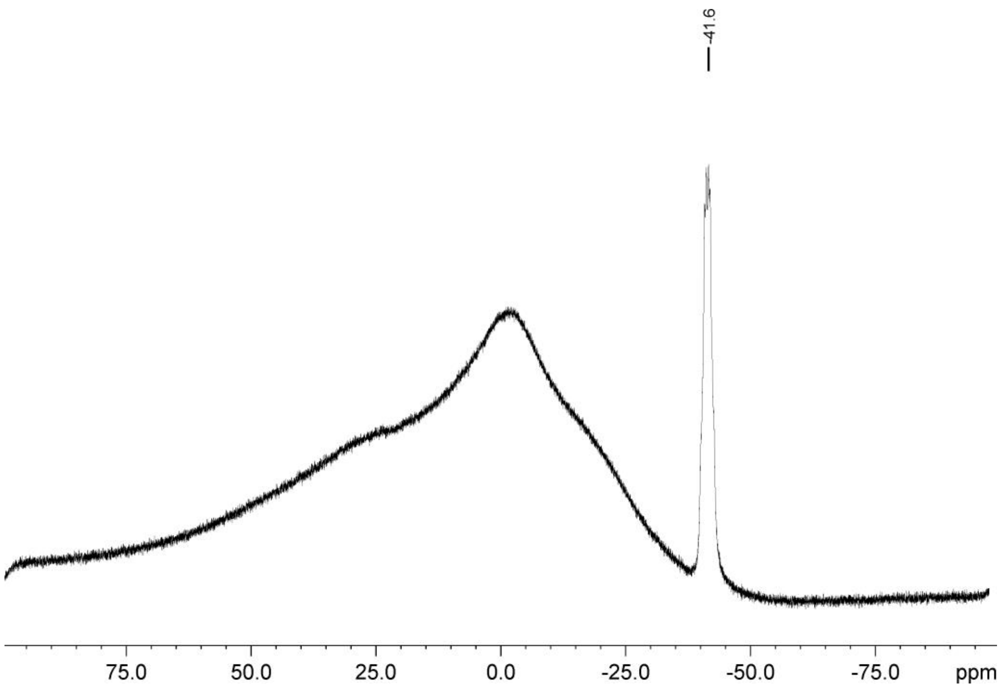

FIG. S51. ^{11}B NMR (C_6D_6) SPECTRUM OF **5A**

NMR spectra of 5b

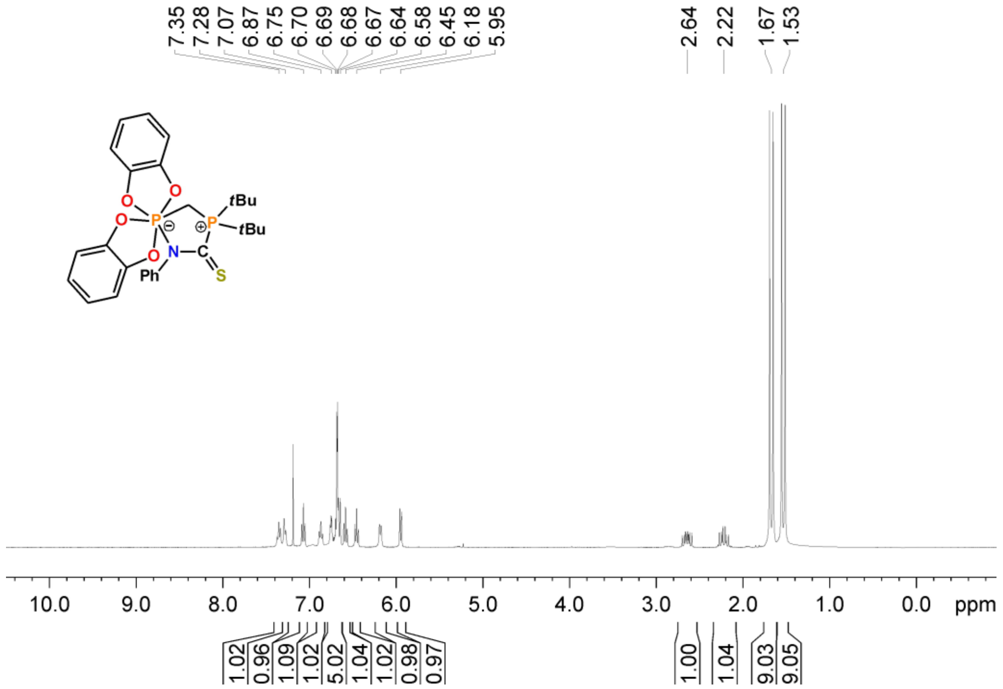

FIG. S52. ^{1}H NMR (C_6D_6) SPECTRUM OF **5B**

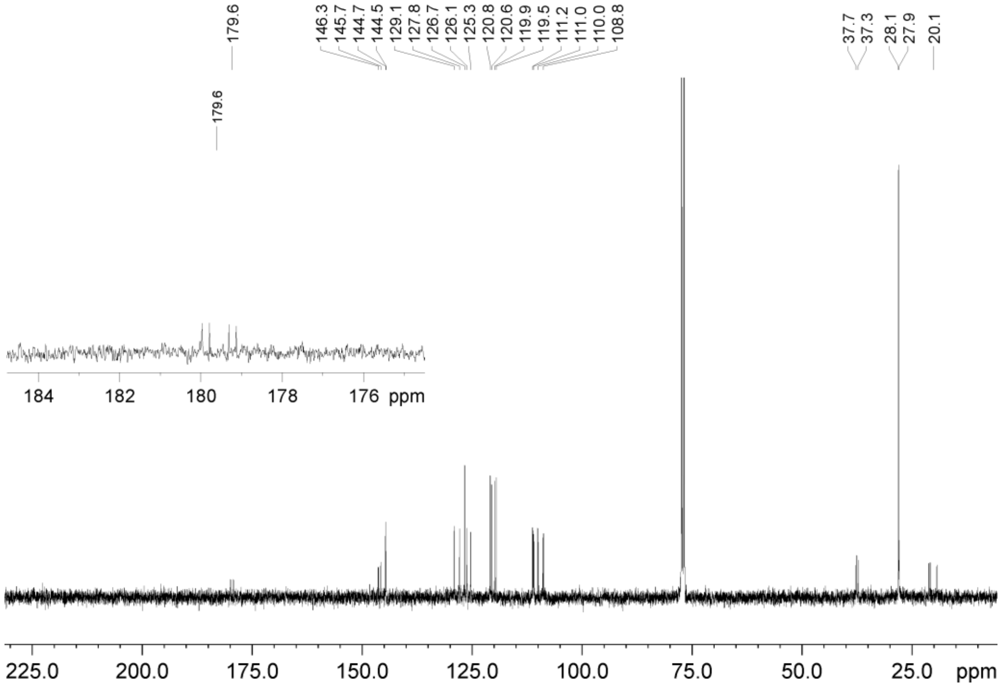

FIG. S53. $^{13}C\{^{1}H\}$ NMR (C_6D_6) SPECTRUM OF **5B**

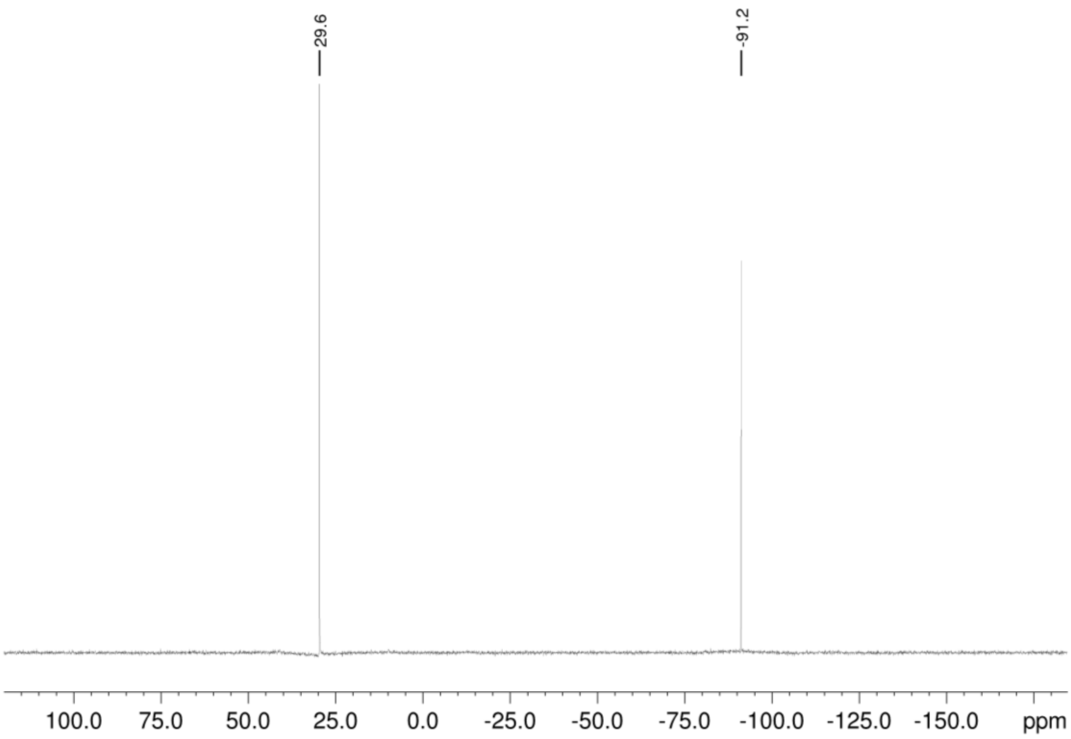

FIG. S54. $^{31}P\{^{1}H\}$ NMR (C_6D_6) SPECTRUM OF **5B**

NMR spectra of 6

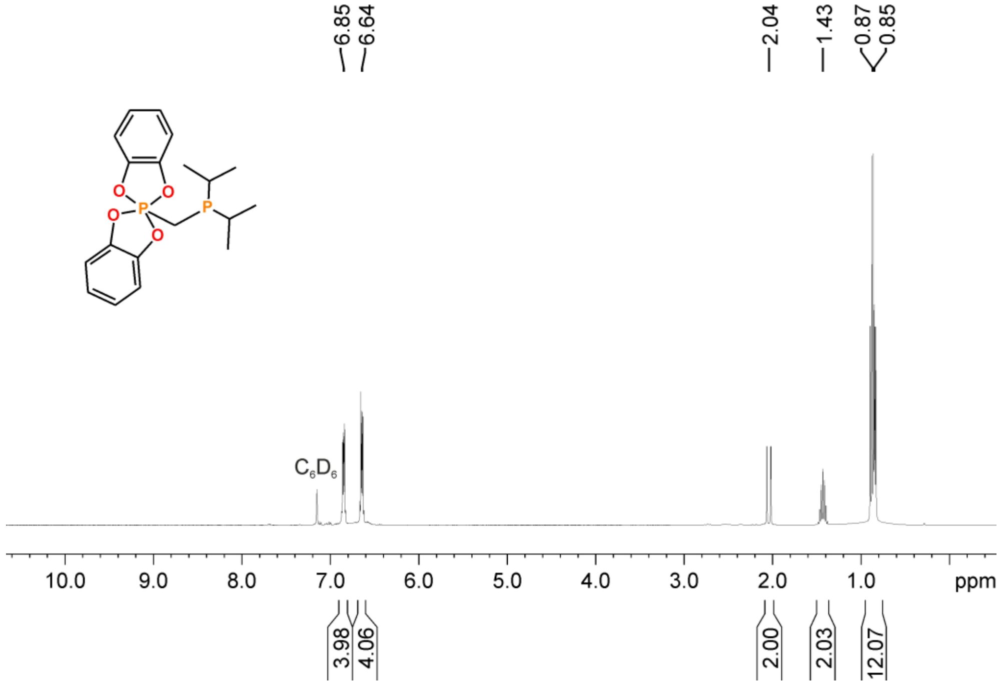

FIG. S55. ^{1}H NMR (C_6D_6) SPECTRUM OF **6**

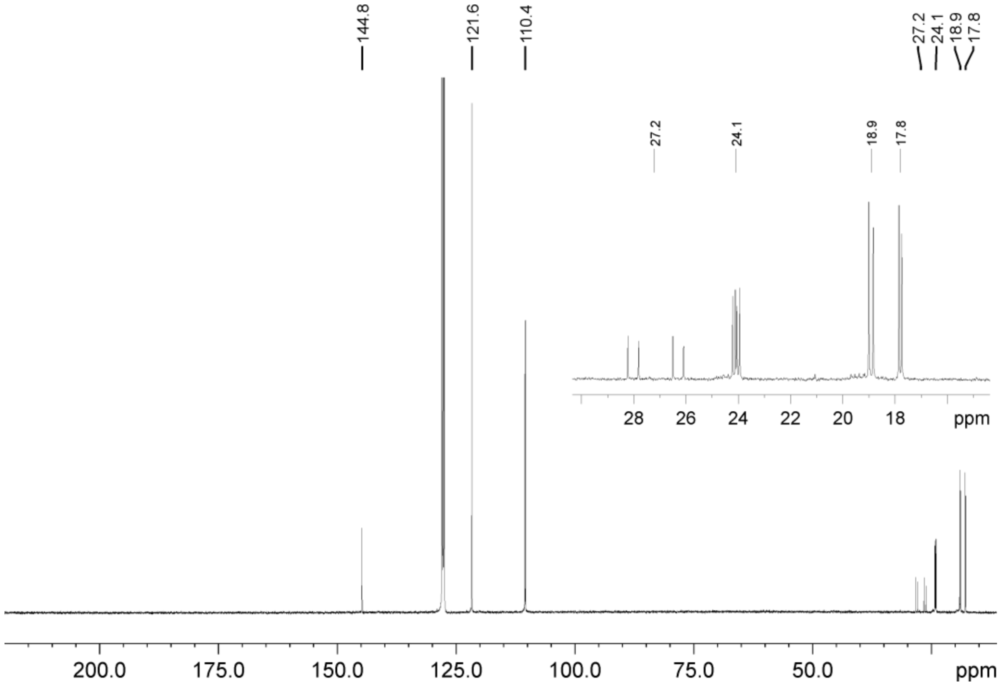

FIG. S56. $^{13}C\{^{1}H\}$ NMR (C_6D_6) SPECTRUM OF **6**

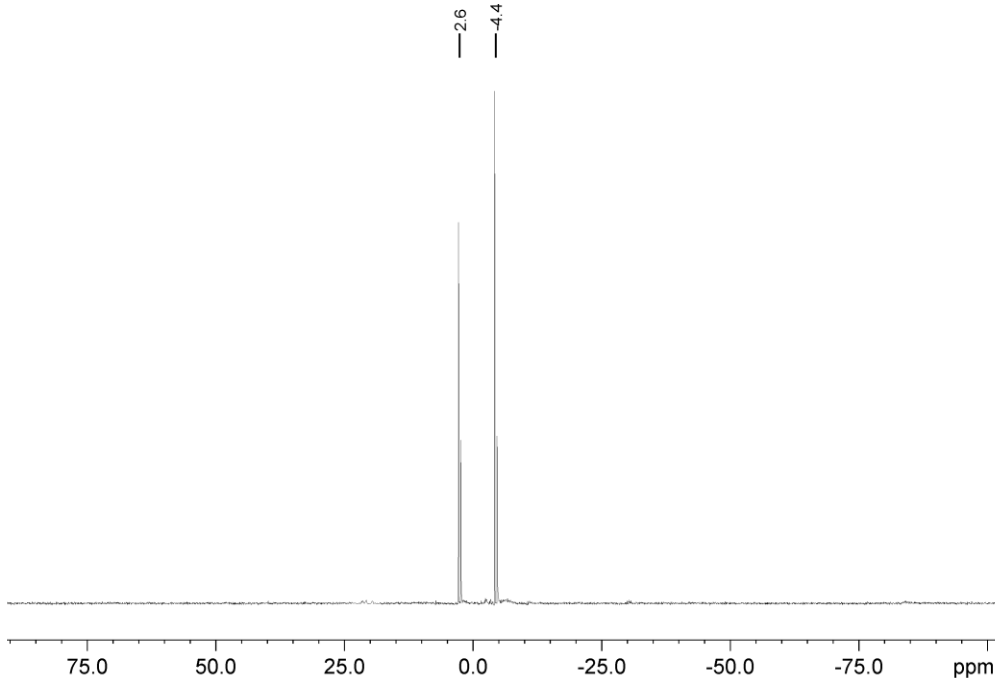

FIG. S57. $^{31}P\{^{1}H\}$ NMR (C_6D_6) SPECTRUM OF **6**

NMR spectra of 6a

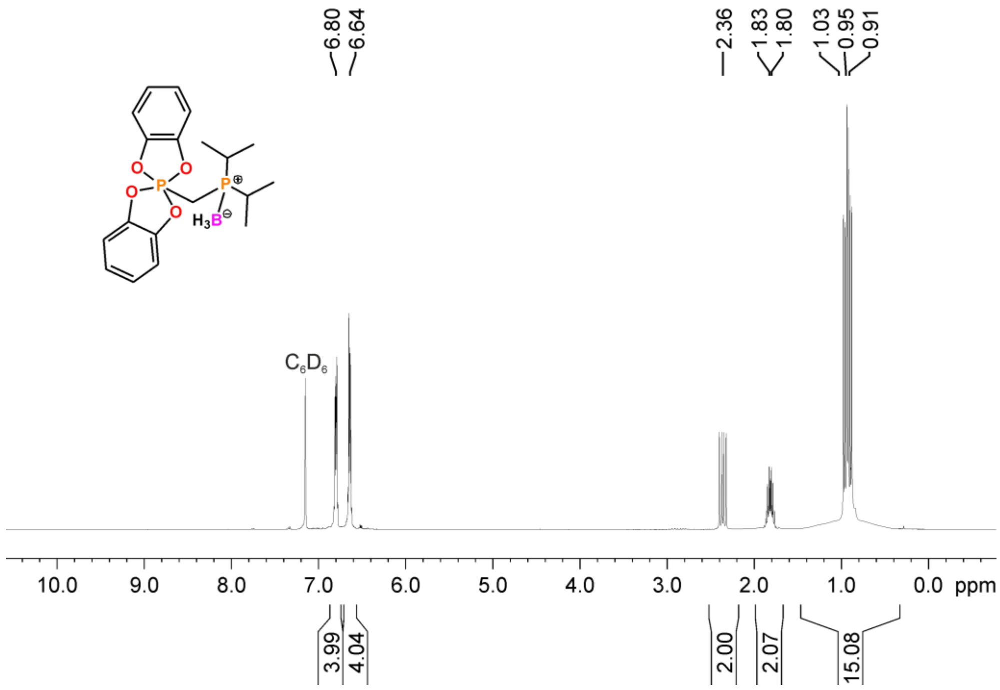

FIG. S58. ^{1}H NMR (C_6D_6) SPECTRUM OF **6A**

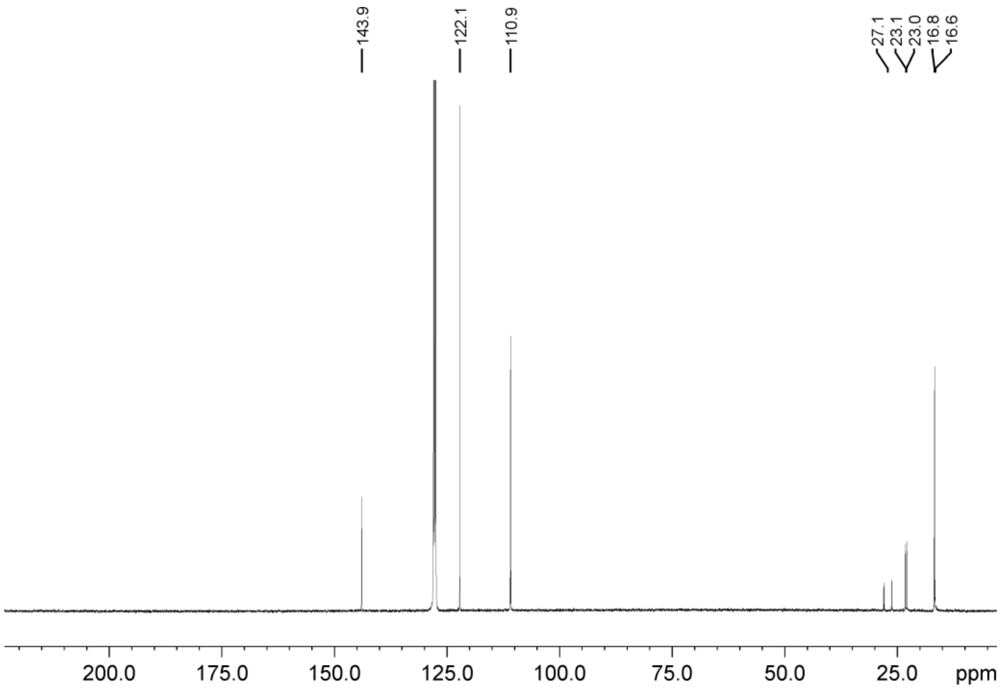

FIG. S59. $^{13}C\{^{1}H\}$ NMR (C_6D_6) SPECTRUM OF **6A**

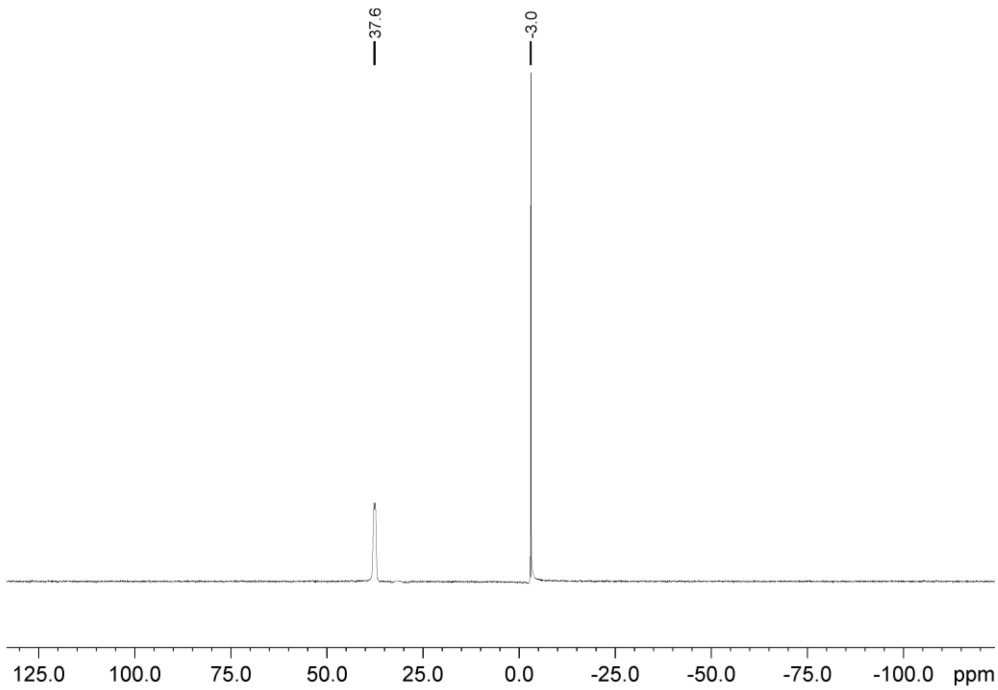

FIG. S60. $^{31}P\{^{1}H\}$ NMR (C_6D_6) SPECTRUM OF **6A**

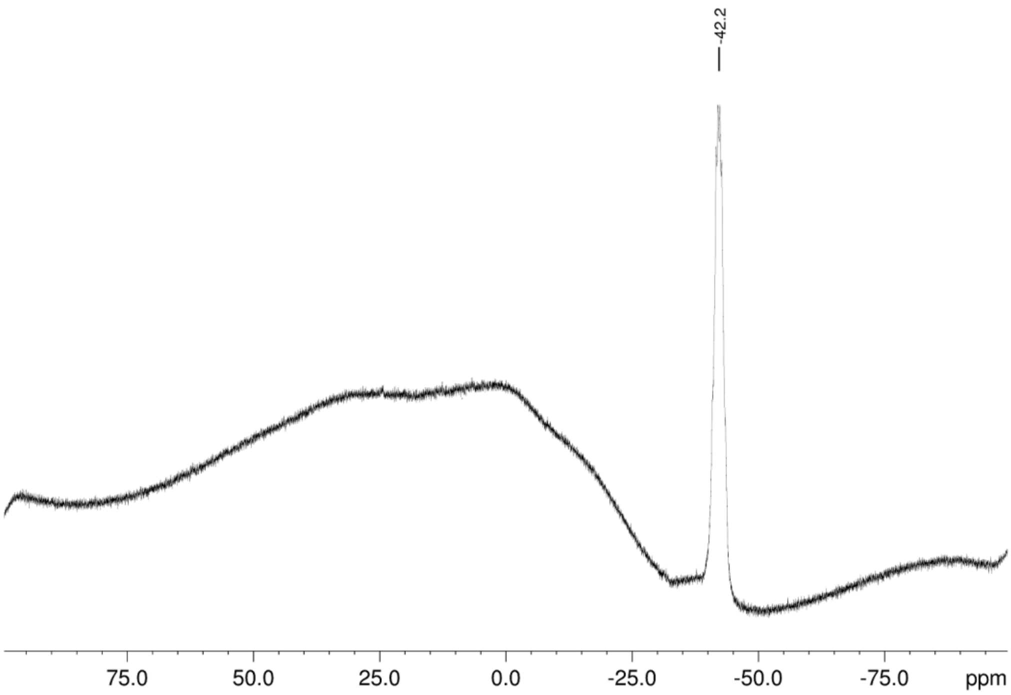

FIG. S61. ^{11}B NMR (C_6D_6) SPECTRUM OF **6A**

NMR spectra of 7

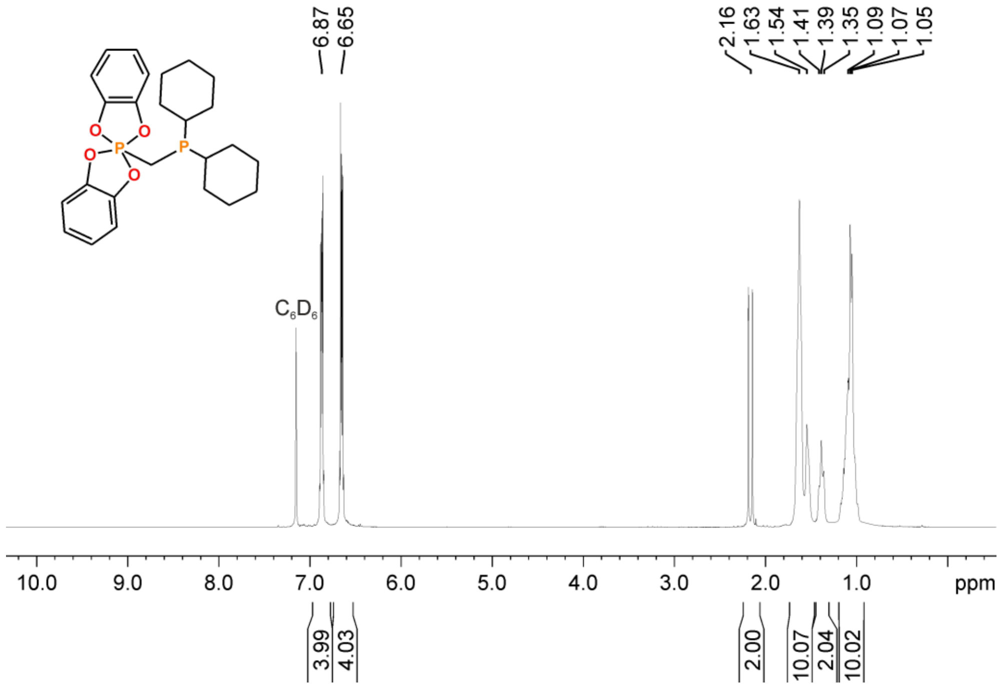

FIG. S62. ^{1}H NMR (C_6D_6) SPECTRUM OF **7**

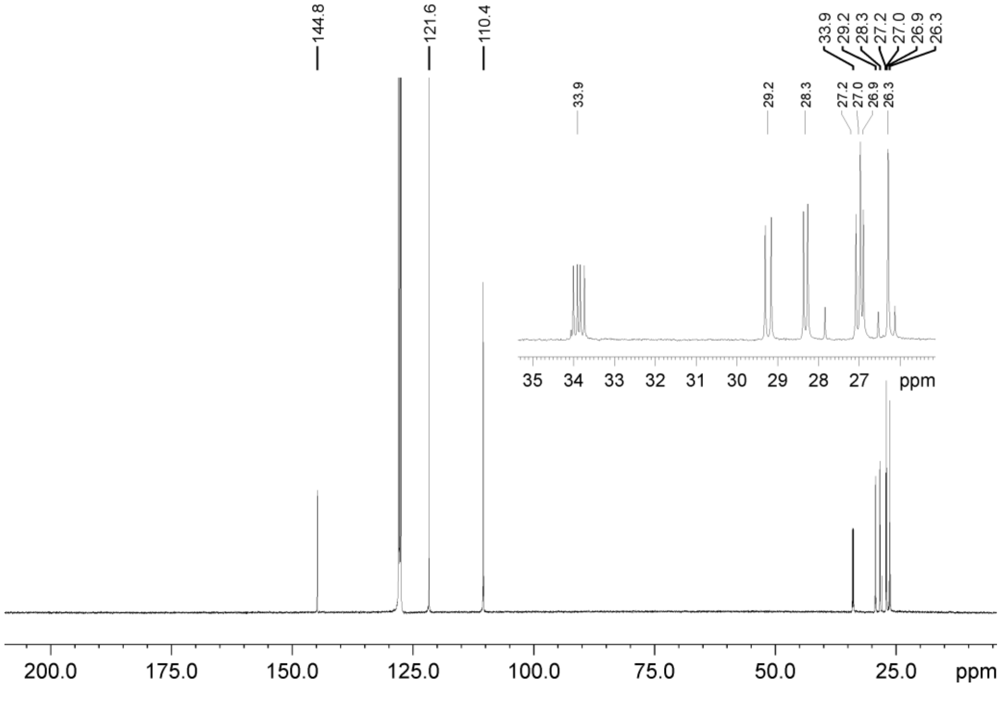

FIG. S63. $^{13}C\{^{1}H\}$ NMR (C_6D_6) SPECTRUM OF **7**

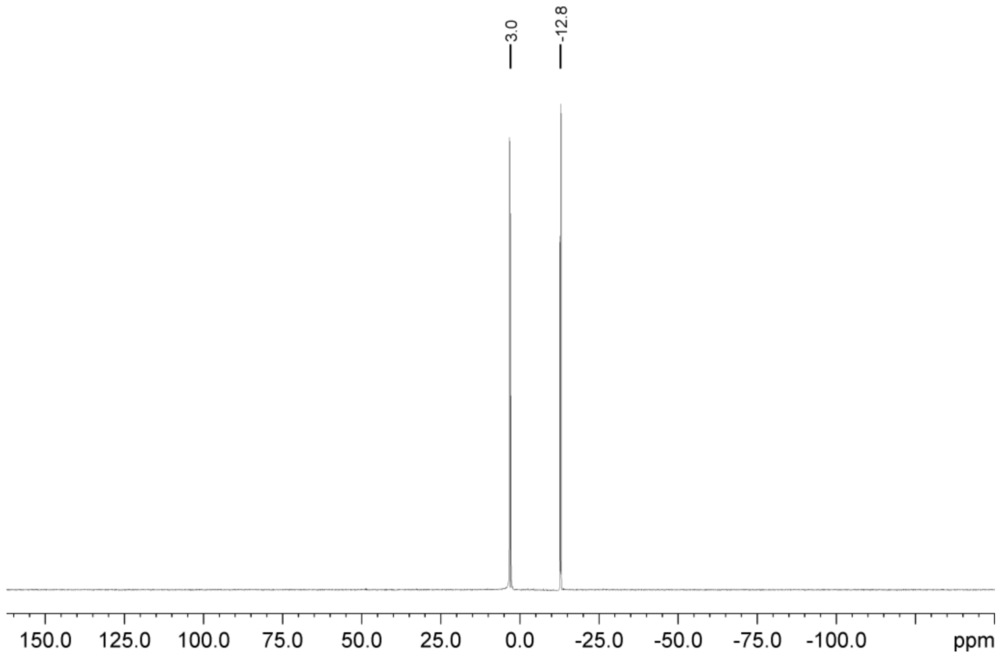

FIG. S64. $^{31}P\{^{1}H\}$ NMR (C_6D_6) SPECTRUM OF **7**

NMR spectra of 7a

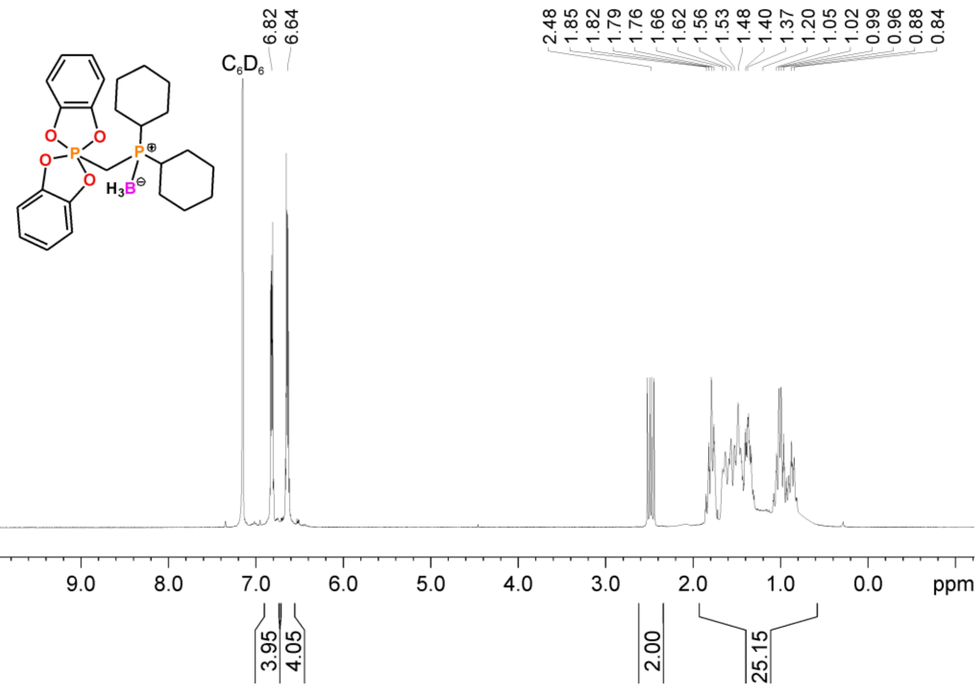

FIG. S65. ^{1}H NMR (C_6D_6) SPECTRUM OF **7A**

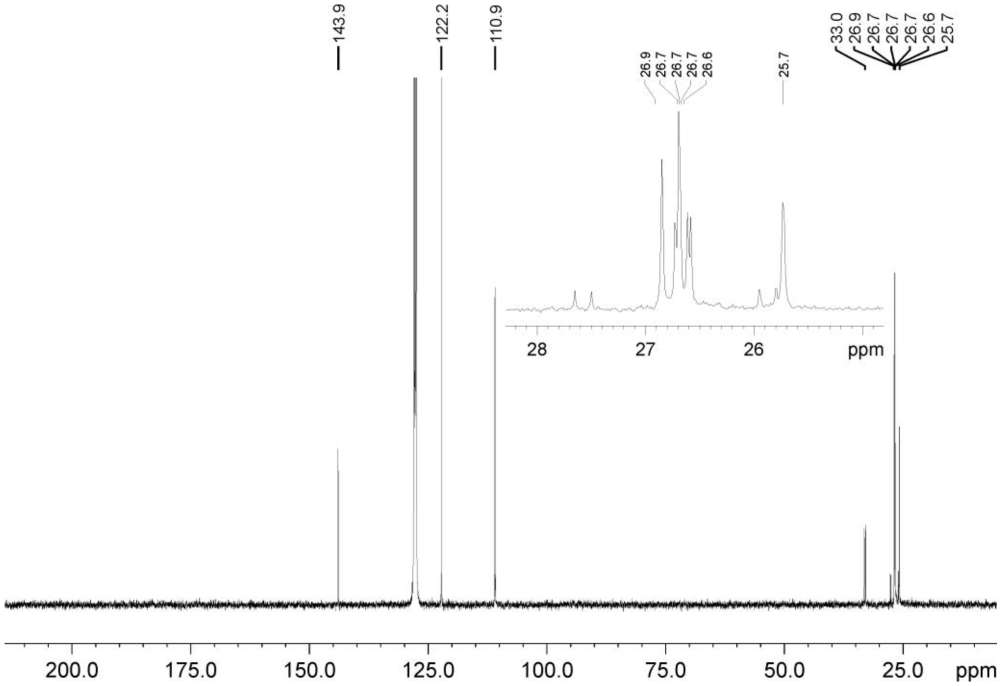

FIG. S66. $^{13}C\{^{1}H\}$ NMR (C_6D_6) SPECTRUM OF **7A**

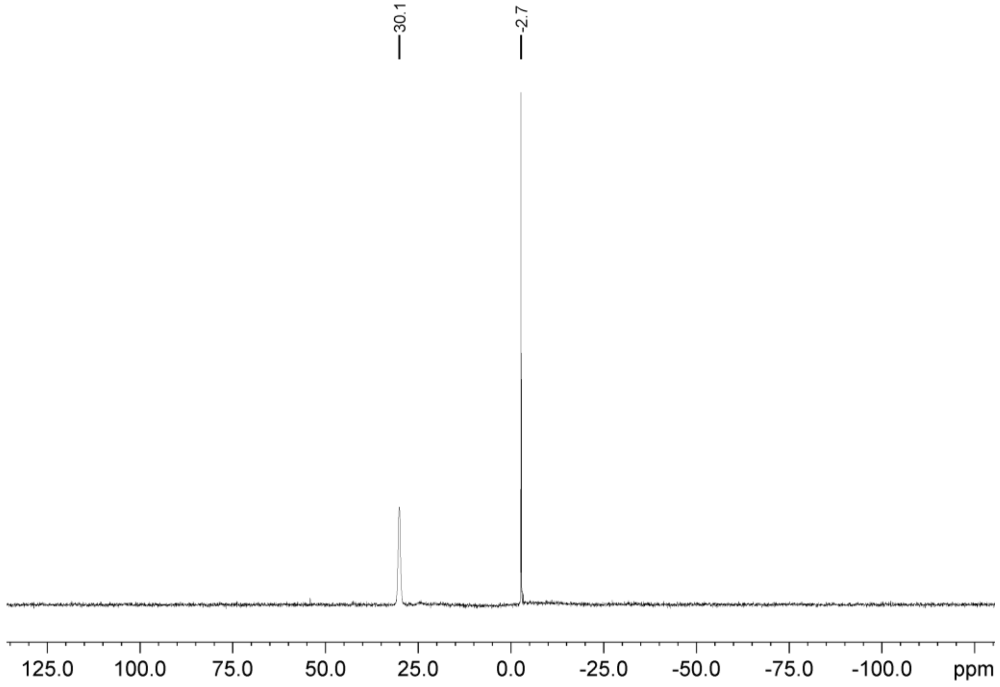

FIG. S67. $^{31}P\{^{1}H\}$ NMR (C_6D_6) SPECTRUM OF **7A**

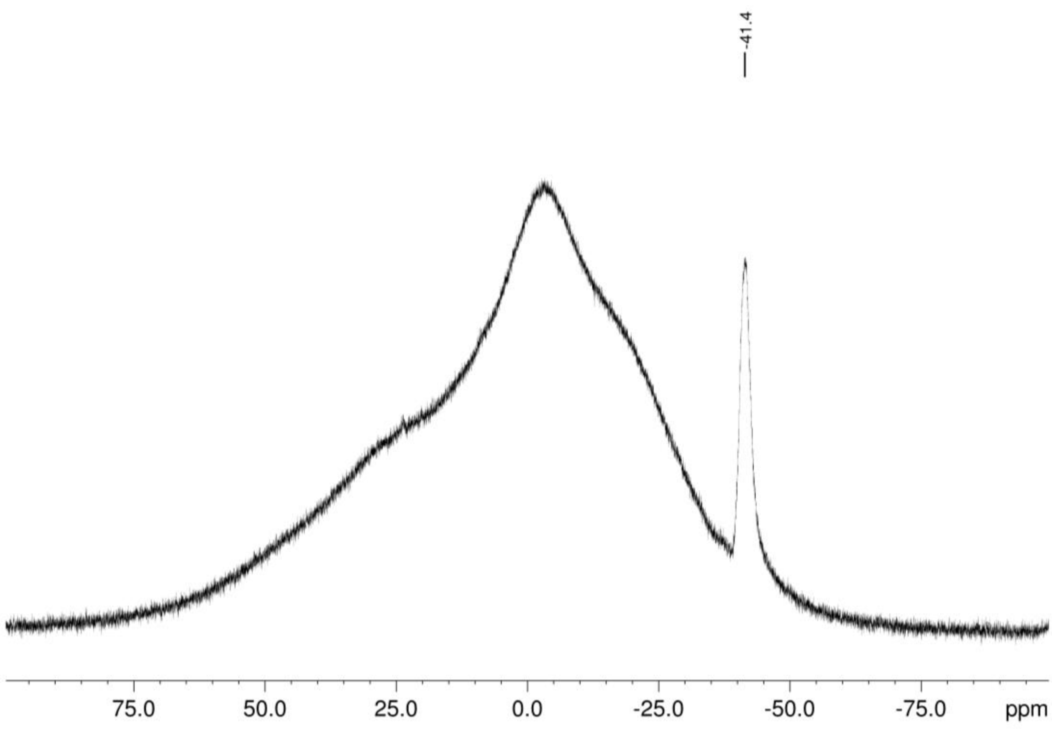

FIG. S68. ^{11}B NMR (C_6D_6) SPECTRUM OF **7A**

IR spectra of isolated compounds

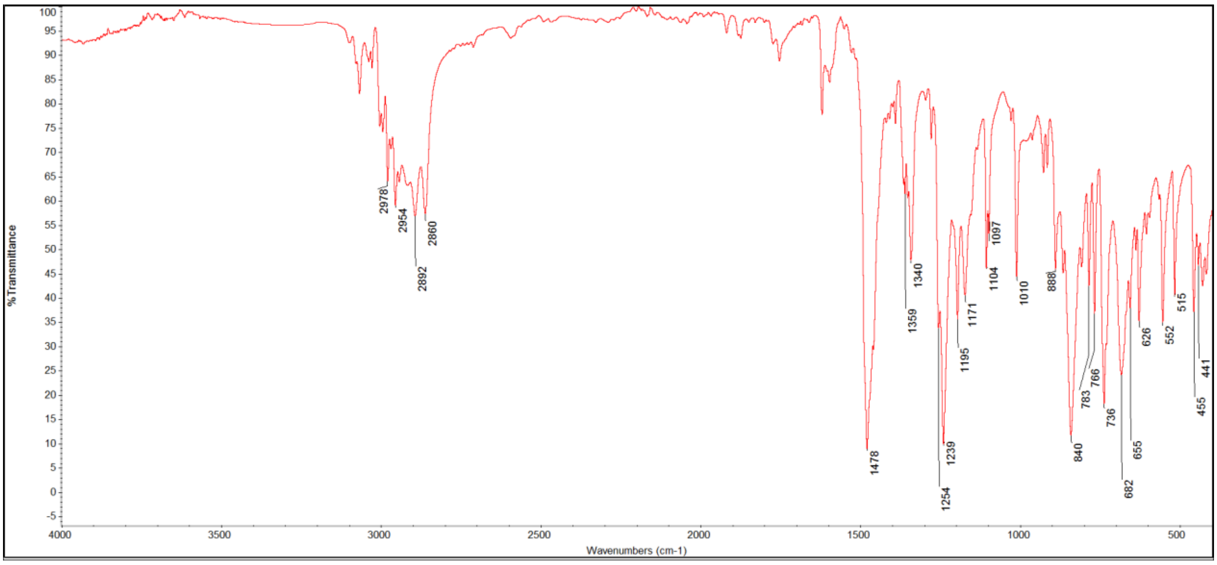

FIG. S69. IR SPECTRUM OF SOLID **1**

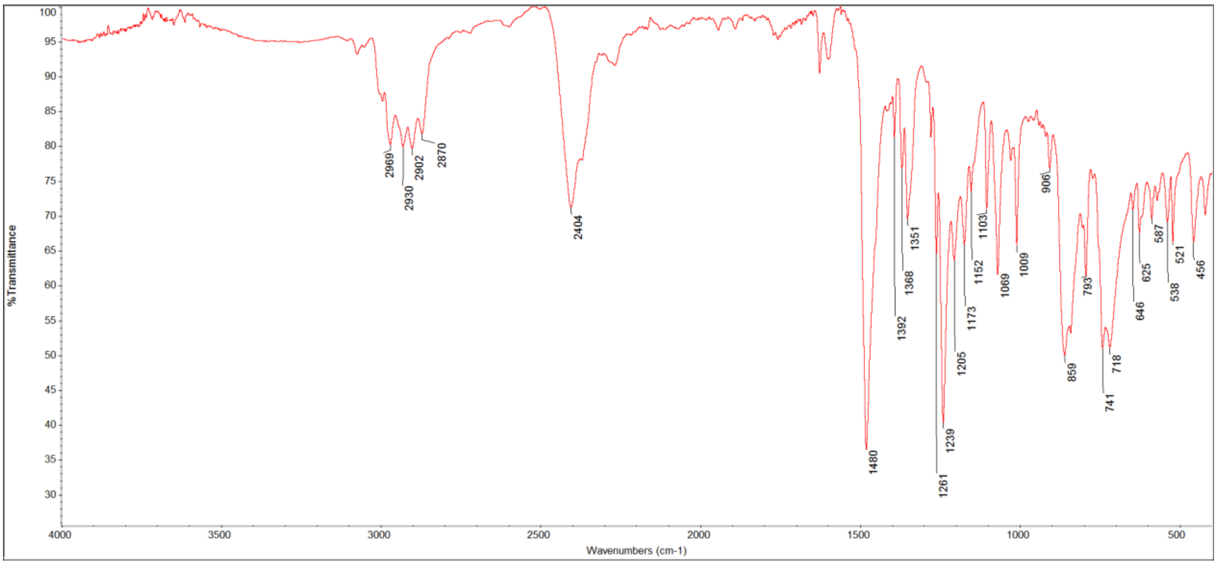

FIG. S70. IR SPECTRUM OF SOLID **1A**

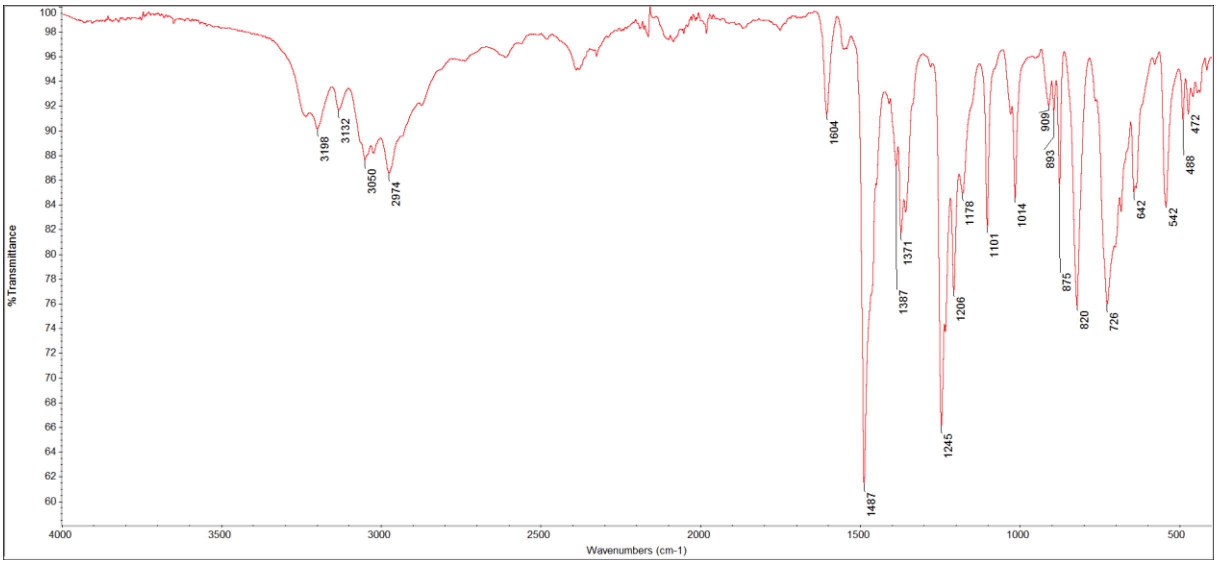

FIG. S71. IR SPECTRUM OF OIL **1B**

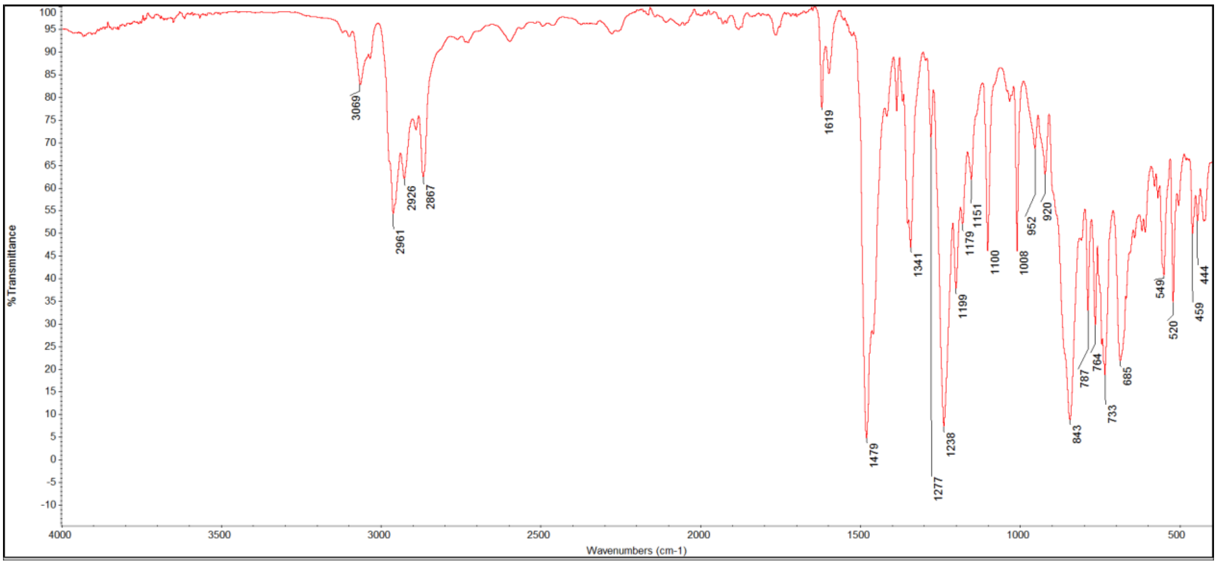

FIG. S72. IR SPECTRUM OF SOLID **2**

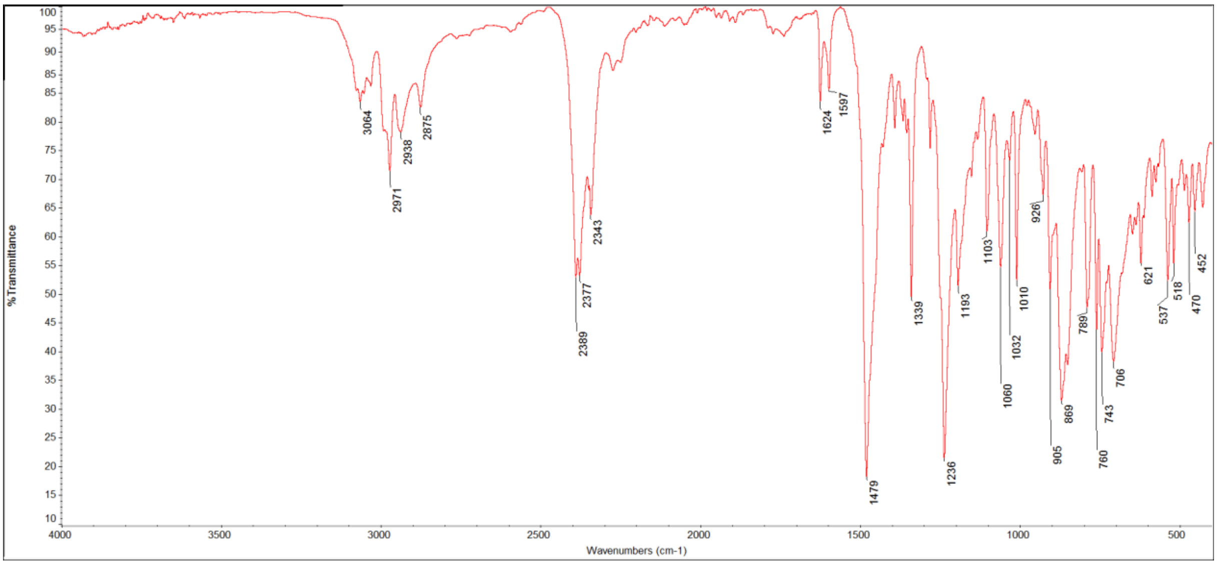

FIG. S73. IR SPECTRUM OF SOLID **2A**

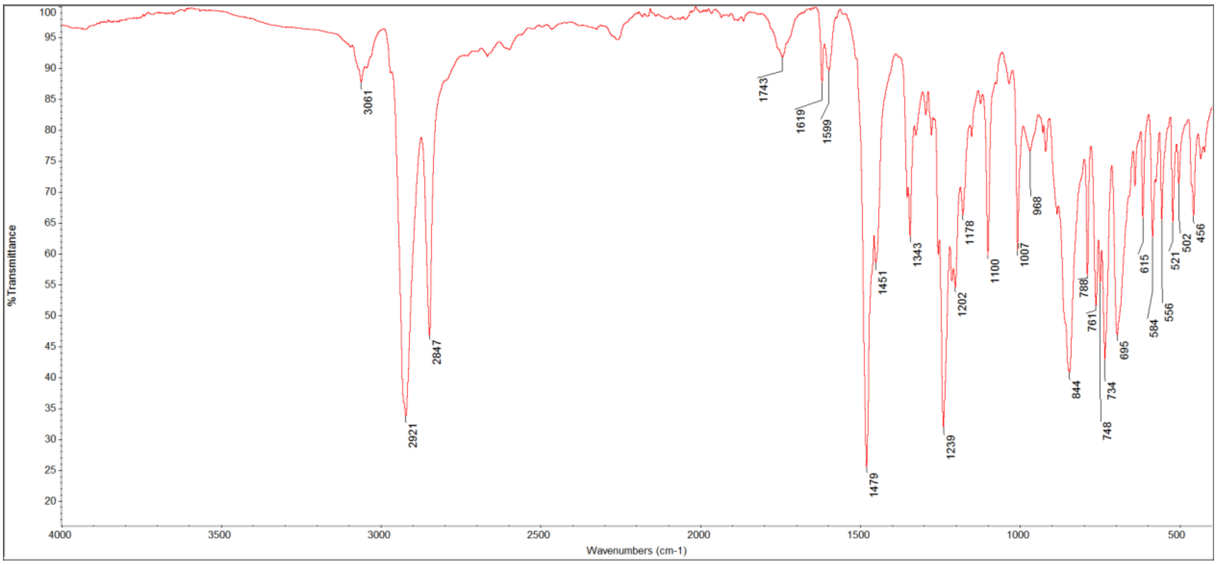

FIG. S74. IR SPECTRUM OF SOLID **3**

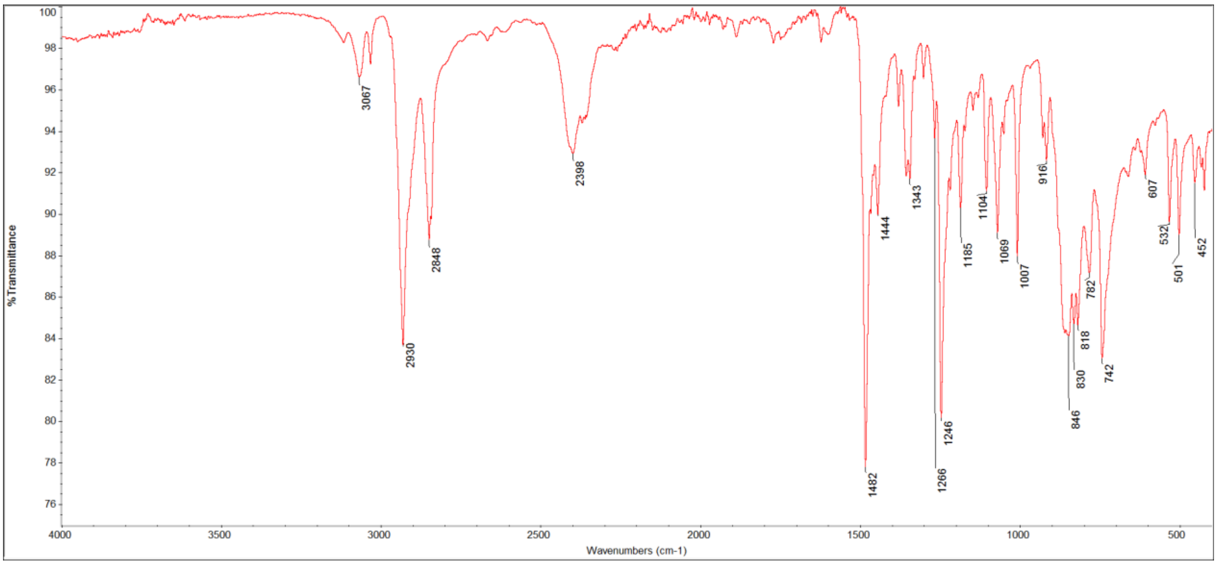

FIG. S75. IR SPECTRUM OF SOLID **3A**

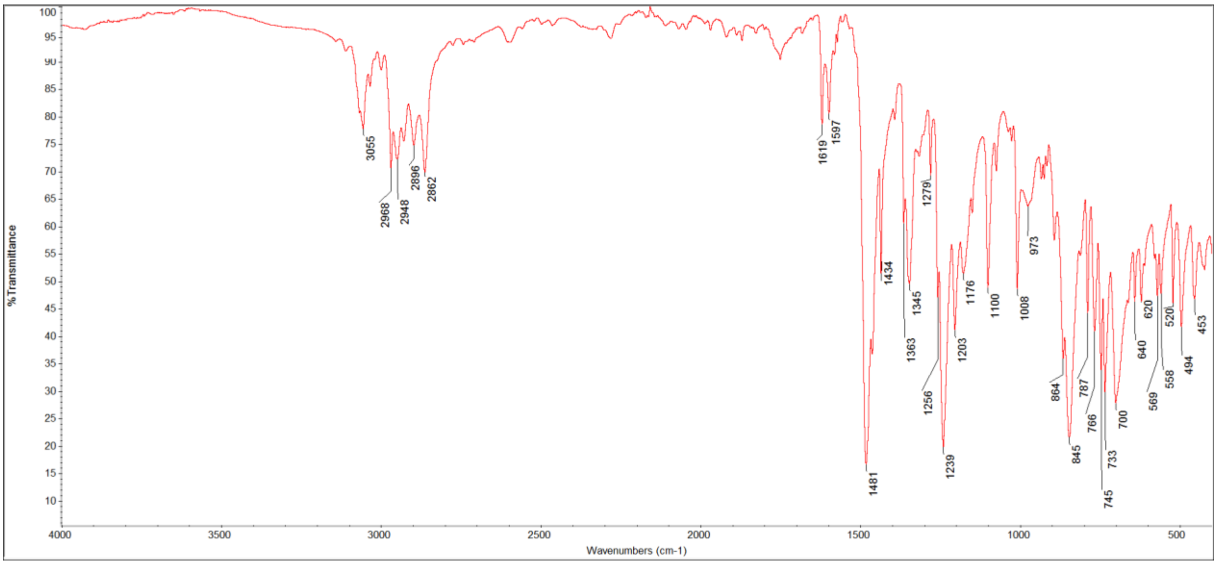

FIG. S76. IR SPECTRUM OF SOLID **4**

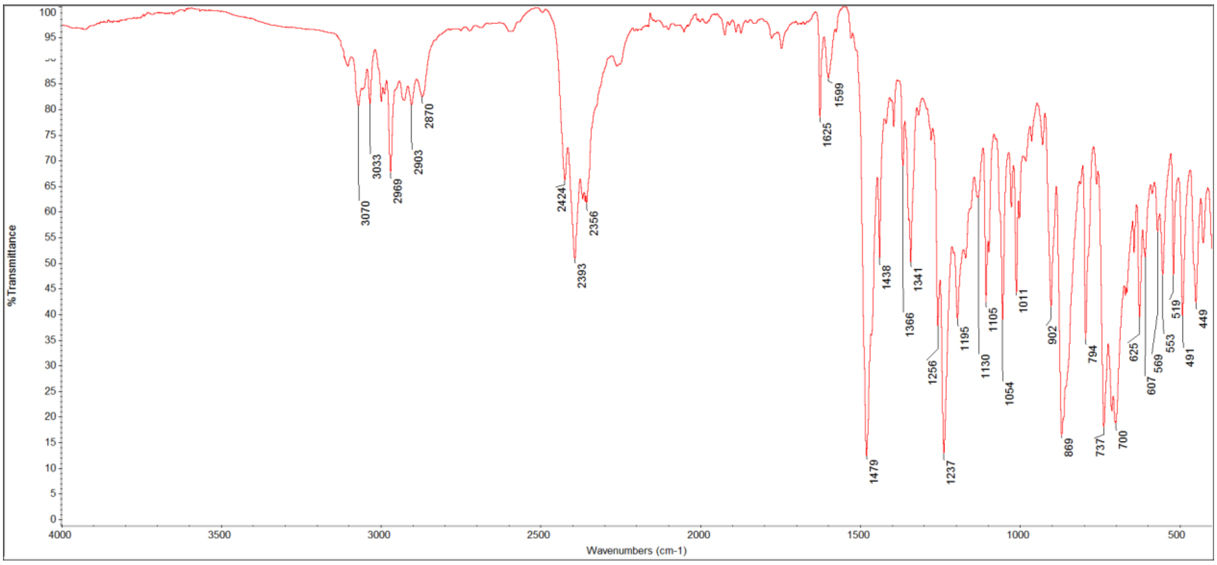

FIG. S77. IR SPECTRUM OF SOLID **4A**

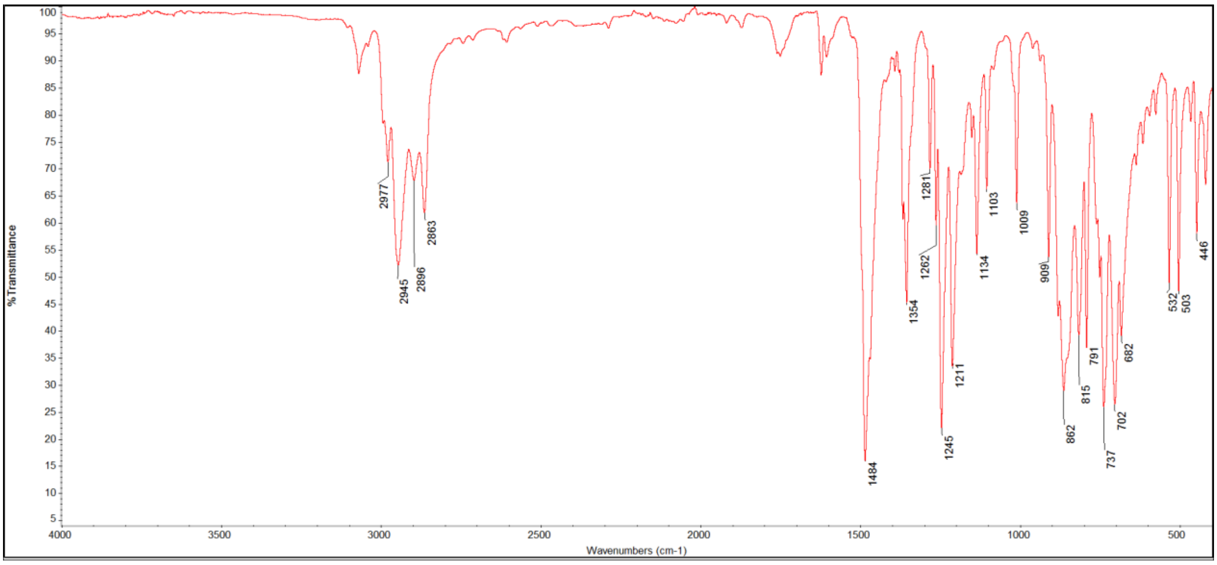

FIG. S78. IR SPECTRUM OF SOLID **5**

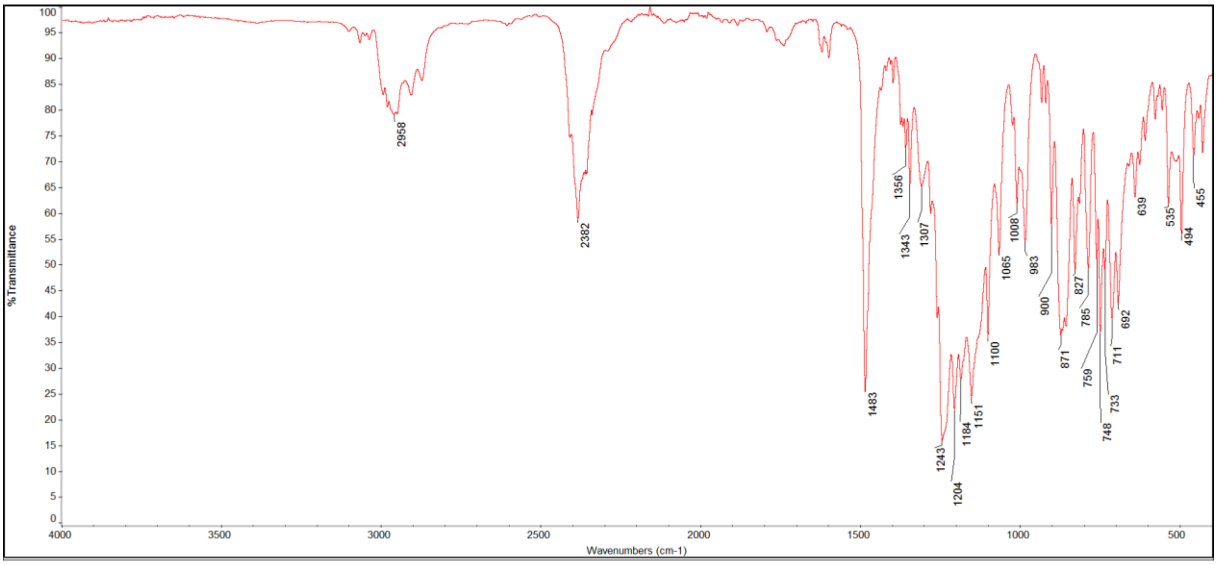

FIG. S79. IR SPECTRUM OF SOLID **5A**

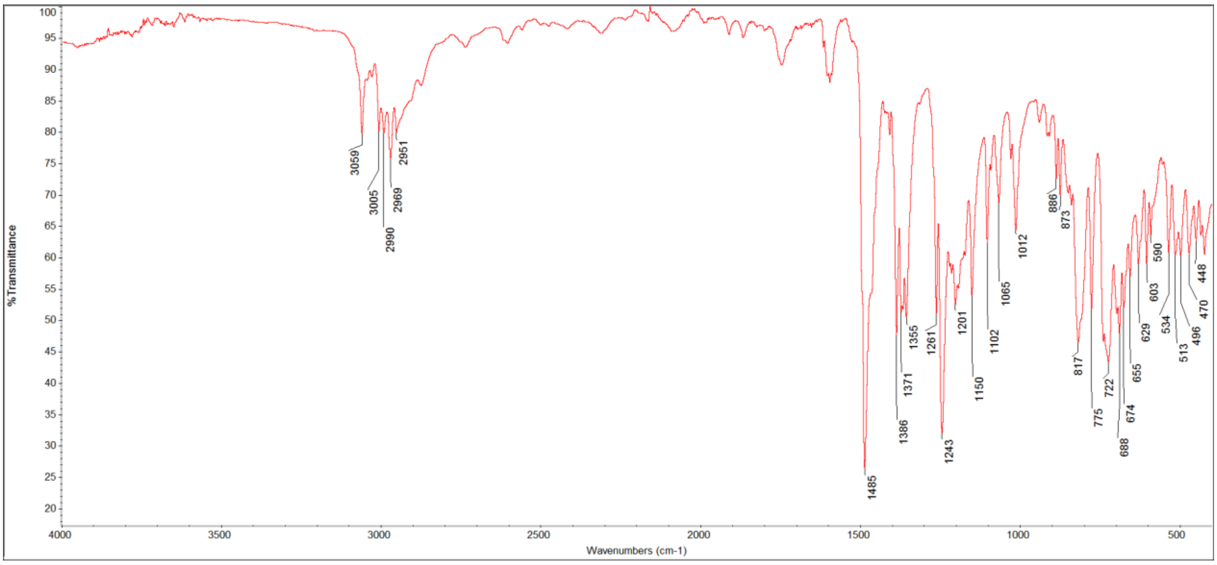

FIG. S80. IR SPECTRUM OF SOLID **5B**

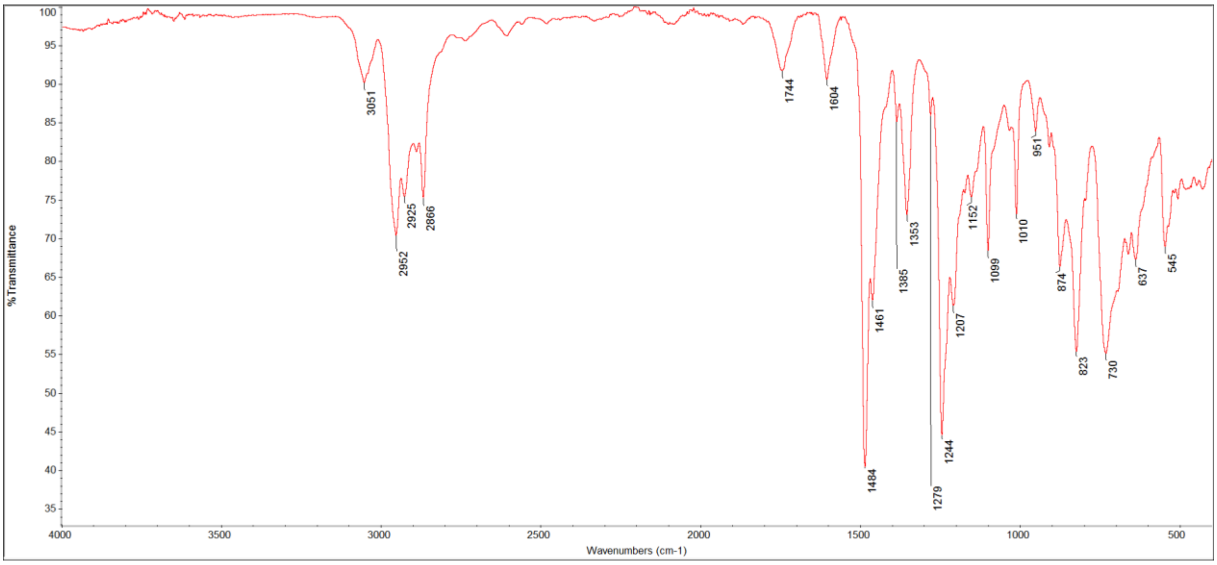

FIG. S81. IR SPECTRUM OF SOLID **6**

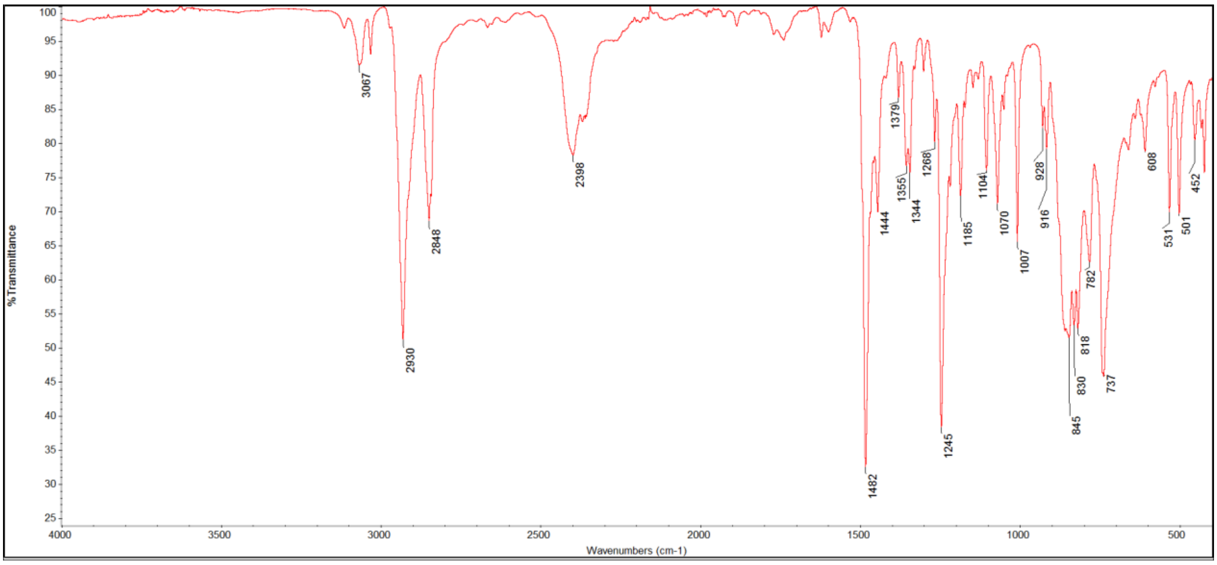

FIG. S82. IR SPECTRUM OF SOLID **6A**

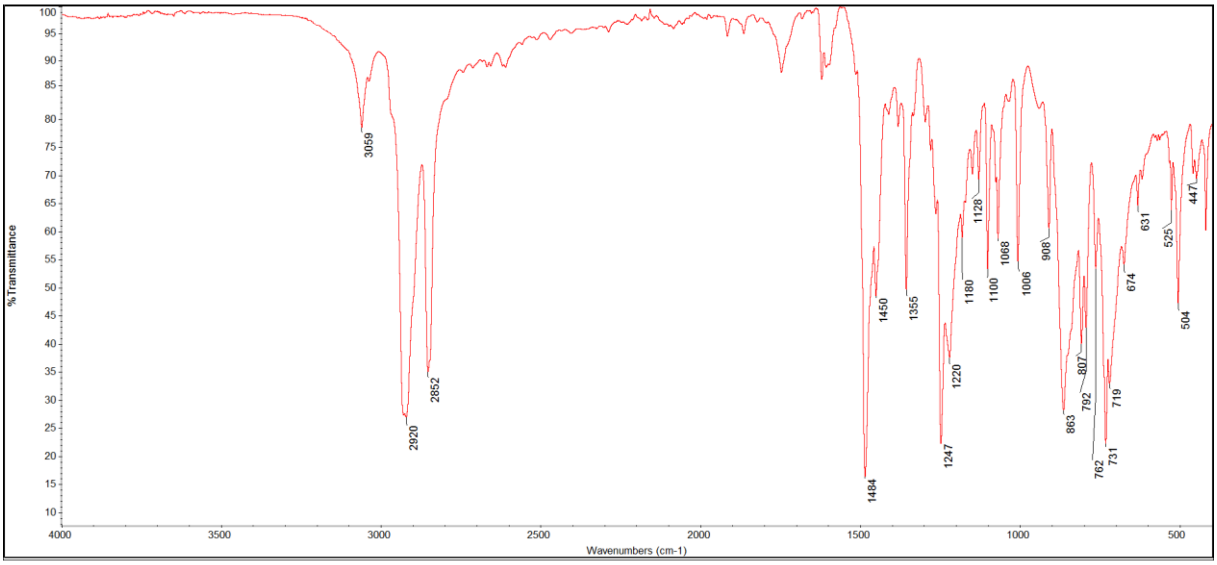

FIG. S83. IR SPECTRUM OF SOLID **7**

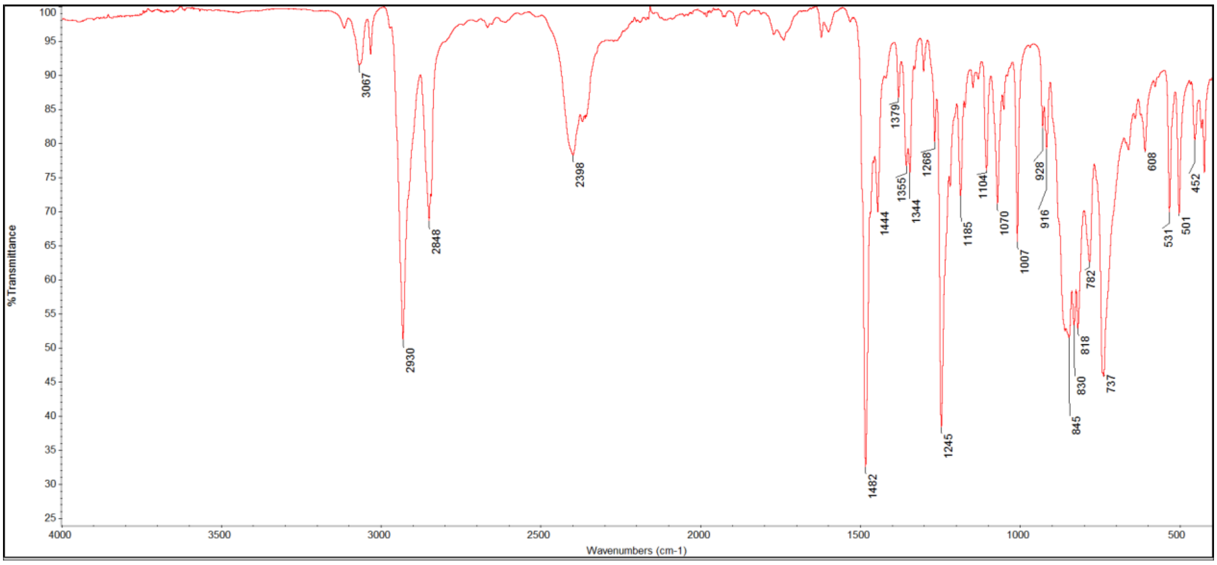

FIG. S84. IR SPECTRUM OF SOLID **7A**

DFT calculations

General methods

All calculations presented in the paper were performed using the Gaussian 09[8] program package. Molecular geometries of all compounds were optimized using density functional theory at the M06-2X functional[9] by with Def2TZVP (**1a-7a**) or 6-31+G(d,p) (**1b**, **5b**) basis set. Molecular geometries were energy-optimized, and the most stable (the lowest energy) conformer was identified during the potential energy surface scanning. Nature of the final gas-phase geometries as local minima (no imaginary frequencies) or transition states (one imaginary frequency) on the potential energy surface was then validated by harmonic frequency calculations at the same level of theory. Values of calculated energies, enthalpies and Gibbs free-energies derived from thermochemical calculations were corrected for the zero-point energy (ZPE). Local maxima related to transition states were established and validated by IRC calculations (to confirm that a located saddle points lie on the minimum energy path between assumed minima) and used to determine energy barriers between respective transformations. Values of energy barriers $\Delta G^{‡}$ and $\Delta H^{‡}$ of reactions **1a-7a**, **1b** and **5b** were determined as the difference between energy of rate-determining transition state and rate-determining intermediate as described in [10]. Harmonic frequency calculations were also used to identify frequencies (stretching vibrations) corresponding with fragments of incorporated molecules (B-H, C=N or C=S bonds) and correctly assign them to the bands on experimental IR spectra. NBO analysis was performed for non-optimized structures derived from X-ray analysis at M06-2X//6-31+G(d,p) level of theory by applying the NBO 3.1[11] module built-in Gaussian 09.

TABLE S22. SELECTED COMPUTATIONAL PARAMETERS OBTAINED FOR CONSIDERED SYSTEMS (IN ATOMIC UNITS A.U.): $\mathbf{E_0}$ - ELECTRONIC ENERGY; $\mathbf{E_{0+...}}$ - SUM OF ELECTRONIC AND: $\mathbf{E_{ZPE}}$ - ZERO-POINT ENERGIES, $\mathbf{E_{THERM}}$ - THERMAL ENERGIES, **H** – THERMAL ENTHALPIES, **G** - THERMAL FREE ENERGIES CALCULATED AT M06-2X//Def2TZVP LEVEL OF THEORY.

Compound	E_{electr} [A.U.]	ε_0 + E_{ZPE} [A.U.]	ε_0 + E_{therm} [A.U.]	ε_0 + H [A.U.]	ε_0 + G [A.U.]
**1**	-1761.415978	-1760.985811	-1760.960638	-1760.959694	-1761.038114
**1a$_{TS}$**	-2266.030040	-2265.491324	-2265.457553	-2265.456609	-2265.556973
**1a**	-1788.061718	-1787.598398	-1787.571483	-1787.570539	-1787.652237
**2**	-1682.808580	-1682.434689	-1682.411904	-1682.410960	-1682.486388
**2a$_{TS}$**	-2187.427053	-2186.944777	-2186.913590	-2186.912646	-2187.006643
**2a**	-1709.457209	-1709.051787	-1709.026841	-1709.025897	-1709.105081
**3**	-1916.265025	-1915.757505	-1915.731282	-1915.730338	-1915.814415
**3a$_{TS}$**	-2420.884150	-2420.268213	-2420.233644	-2420.232700	-2420.335723
**3a**	-1942.913613	-1942.374323	-1942.345993	-1942.345048	-1942.432741
**4**	-1835.232356	-1834.833933	-1834.809274	-1834.808329	-1834.888541
**4a$_{TS}$**	-2339.848959	-2339.342447	-2339.309269	-2339.308325	-2339.409665
**4a**	-1861.878661	-1861.448442	-1861.421673	-1861.420729	-1861.505136
**5**	-1800.733492	-1800.275730	-1800.249142	-1800.248197	-1800.331964
**5a$_{TS}$**	-2305.351612	-2304.784739	-2304.750190	-2304.749245	-2304.850817
**5a**	-1827.386346	-1826.895205	-1826.867139	-1826.866194	-1826.952059
**6**	-1722.122853	-1721.721364	-1721.697222	-1721.696278	-1721.776401
**6a$_{TS}$**	-2226.741182	-2226.231261	-2226.198567	-2226.197622	-2226.296754
**6a**	-1748.777905	-1748.344255	-1748.318071	-1748.317127	-1748.399706
**7**	-1955.578605	-1955.043106	-1955.015781	-1955.014837	-1955.101765
**7a$_{TS}$**	-2460.196490	-2459.552962	-2459.516987	-2459.516043	-2459.623900
**7a**	-1982.231638	-1981.663879	-1981.634530	-1981.633586	-1981.723844
**BH_3·SMe_2**	-504.627112	-504.518982	-504.512133	-504.511189	-504.548421
**SMe_2**	-477.989402	-477.913246	-477.908398	-477.907453	-477.940217

TABLE S23. SELECTED COMPUTATIONAL PARAMETERS OBTAINED FOR CONSIDERED SYSTEMS (IN ATOMIC UNITS A.U.): **E_0** - ELECTRONIC ENERGY; **$E_{0+...}$** - SUM OF ELECTRONIC AND: **E_{ZPE}** - ZERO-POINT ENERGIES, **E_{THERM}** - THERMAL ENERGIES, **H** – THERMAL ENTHALPIES, **G** - THERMAL FREE ENERGIES CALCULATED AT M06-2X//6-31+G(d,p) LEVEL OF THEORY.

Compound	E_{electr} [A.U.]	$\varepsilon_0 + E_{ZPE}$ [A.U.]	$\varepsilon_0 + E_{therm}$ [A.U.]	ε_0 + H [A.U.]	ε_0 + G [A.U.]
**1**	-1760.971144	-1760.540780	-1760.515454	-1760.514510	-1760.593235
**$1b_{TS1}$**	-2483.488658	-2482.955669	-2482.921989	-2482.921045	-2483.019868
**$1b_{I1}$**	-2483.502527	-2482.966771	-2482.933406	-2482.932462	-2483.029328
**$1b_{TS2}$**	-2483.497861	-2482.961864	-2482.929351	-2482.928407	-2483.023066
**$1b_{I2}$**	-2483.526931	-2482.990225	-2482.957302	-2482.956358	-2483.052159
**$1b_{TS3}$**	-2483.499061	-2482.963582	-2482.931071	-2482.930126	-2483.025536
**$1b_{I3}$**	-2483.509507	-2482.973864	-2482.940685	-2482.939741	-2483.037574
**$1b_{TS4}$**	-2483.508806	-2482.973731	-2482.941155	-2482.940211	-2483.036596
**1b**	-2483.528824	-2482.993393	-2482.960171	-2482.959226	-2483.057950
**5**	-1800.272651	-1799.814787	-1799.788005	-1799.787061	-1799.871602
**$5b_{TS}$**	-2522.834149	-2522.269202	-2522.235365	-2522.234420	-2522.336232
**5b**	-2522.834149	-2522.269202	-2522.235365	-2522.234420	-2522.336232
**5b'**	-2522.816583	-2522.252656	-2522.218456	-2522.217512	-2522.317933
**PhNCS**	-722.530616	-722.428334	-722.420778	-722.419834	-722.462484

Values of free energy of formation of considered products

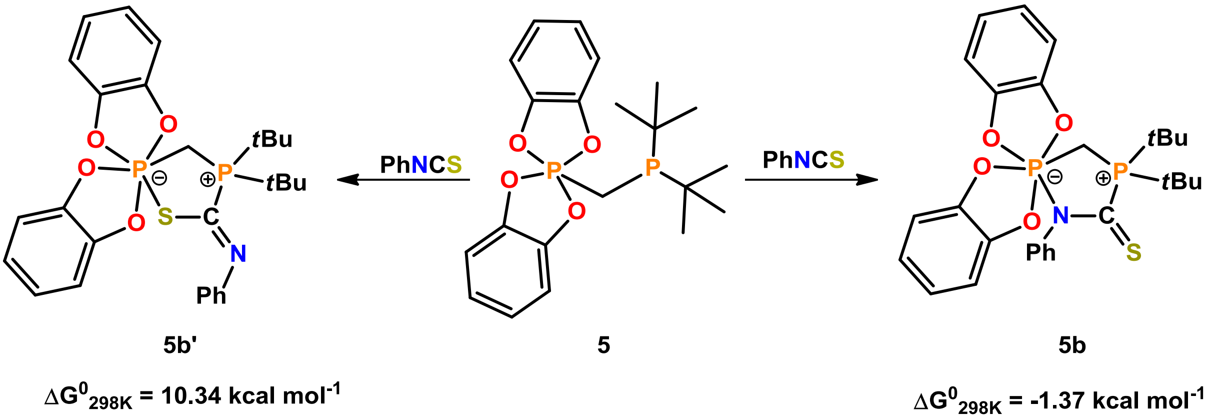

SCHEME. S1. FREE ENERGY VALUES OF FORMATION OF PRODUCTS RESULTING FROM THE REACTION OF **5** WITH PhNCS.

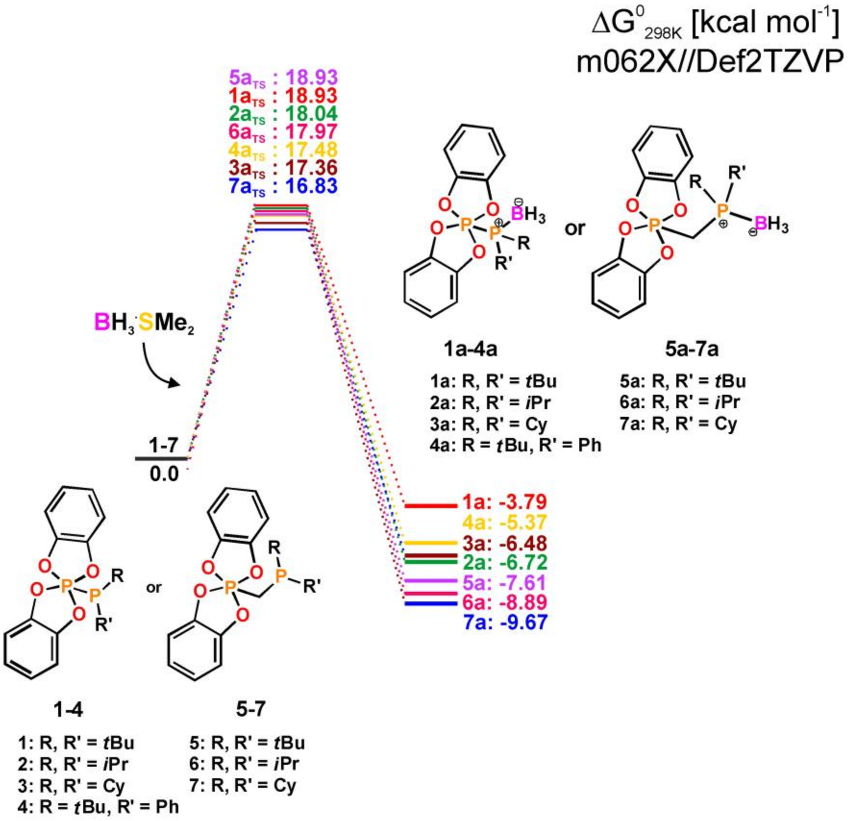

SCHEME. S2. FREE ENERGY VALUES OF FORMATION IN THE REACTION OF **1-7** WITH BH_3.

TABLE S24. SELECTED COMPUTATIONAL PARAMETERS OBTAINED FOR CONSIDERED SYSTEMS (IN ATOMIC UNITS A.U.): **$\Delta H^{\#}_{298K}$** – THERMAL ENTHALPHY OF ACTIVATION, **$\Delta G^{\#}_{298K}$** – GIBBS FREE ENERGY OF ACTIVATION, **k** – RATE CONSTANT, **ΔH^{0}_{298K}** – THERMAL ENTHALPHY OF REACTION, **ΔG^{0}_{298K}** – GIBBS FREE ENERGY OF REACTION, **K_{298K}** – EQUILIBRIUM CONSTANT OF THE REACTION CALCULATED AT M06-2X//Def2TZVP LEVEL OF THEORY.

Reaction	$\Delta H^{\#}_{298K}$ [kcal mol-1]	$\Delta G^{\#}_{298K}$ [kcal mol-1]	k [s-1]	ΔH^{0}_{298K} [kcal mol-1]	ΔG^{0}_{298K} [kcal mol-1]	K_{298K}
**1a**	9.14	18.93	0.15	-4.55	-3.79	5.30E+02
**2a**	6.09	18.04	0.68	-7.17	-6.72	6.72E+04
**3a**	5.65	17.36	2.07	-7.03	-6.48	4.55E+04
**4a**	7.17	17.48	1.70	-5.55	-5.37	7.27E+03
**5a**	6.49	18.93	0.15	-9.13	-7.61	2.97E+05
**6a**	6.30	17.97	0.75	-10.96	-9.67	8.91E+06
**7a**	6.39	16.83	4.96	-9.61	-8.89	2.43E+06

The value of rate constant was calculated based on transition state theory, using the relationship between the rate constant $\boldsymbol{k}(\mathbf{T})$ and the Gibbs free energy of activation $\boldsymbol{\Delta G^{\#}_{T}}$ where $\boldsymbol{h}$ is the Planck constant and $\boldsymbol{R}$ the molar gas constant:

$$k(T) = \frac{k_B T}{h} e^{\frac{-\Delta G^{\#}}{RT}} \qquad (S1)$$

Optimized structures and Cartesian coordinates

To ease following the reaction mechanisms and provide quick access to the structural properties of all substrates, intermediates, transition states and products, their Cartesian coordinates were collected in one cif file. It is added to this ESI pdf file as the attachment, which contains a list of DFT-optimized molecular structures from which the xyz file may be extracted if needed.

Optimized molecular structures.cif

References

(1) Fink, L.; Samigullin, K.; Bodach, A.; Alig, E.; Wagner, M.; Lerner, H. W. Donor-Unsupported Phosphanylmethanides Li[CH2PR2] (R = TBu, Ph) - Crystal Structure of Li[CH2PtBu2] Solved by XRPD and DFT-D Calculations. *Zeitschrift fur Anorg. und Allg. Chemie* **2016**, *642* (3), 282–287. https://doi.org/10.1002/zaac.201500811.

(2) Ramirez, F.; Bigler, A. J.; Smith, C. P. Phosphoranylations. Preparation of 5-Membered Cyclic Oxyphosphoranes and Spirooxyphosphoranes from the Reaction of Pentaphenoxyphosphorane with Catechol. *Tetrahedron* **1968**, *24* (14), 5041–5051. https://doi.org/10.1016/S0040-4020(01)88414-3.

(3) STOE &Cie GmbH, X-Area 1.75, STOE &Cie GmbH. Darmstadt, Germany 2015.

(4) Sheldrick, G. M. SHELXT - Integrated Space-Group and Crystal-Structure Determination. *Acta Crystallogr. Sect. A Found. Crystallogr.* **2015**, *A71*, 3–8.

(5) Sheldrick, G. M. Crystal Structure Refinement with SHELXL. *Acta Cryst. C* **2015**, *71*, 3–8. https://doi.org/10.1107/S2053229614024218.

(6) Westrip, S. P. PublCIF: Software for Editing, Validating and Formatting Crystallographic Information Files. *J. Appl. Crystallogr.* **2010**, *43*, 920–925.

(7) Dolomanov, O. V; Bourhis, L. J.; Gildea, R. J.; Howard, J. A. K.; Puschmann, H. OLEX2: A Complete Structure Solution, Refinement and Analysis Program. *J. Appl. Crystallogr.* **2009**, *42*, 339–341. https://doi.org/10.1107/S0021889808042726.

(8) Frisch, M. J.; Trucks, G. W.; Schlegel, H. B.; Scuseria, G. E.; Robb, M. A.; Cheeseman, J. R.; Scalmani, G.; Barone, V.; Petersson, G. A.; Nakatsuji, H.; Li, X.; Caricato, M.; Marenich, A.; Bloino, J.; Janesko, B. G.; Gomperts, R.; Mennucci, B.; Hratchian, H. P.; Ortritz, J. V.; Izmaylov, A. F.; Sonnenberg, J. L.; Williams-Young, D.; Ding, F.; Lipparini, F.; Egidi, F.; Goings, J.; Peng, B.; Petrone, A.; Henderson, T.; Ranasinghe, D.; Zakrzewski, V. G.; Gao, J.; Rega, N.; Zheng, G.; Liang, W.; Hada, M.; Ehara, M.; Toyota, K.; Fukuda, R.; Hasegawa, J.; Ishida, M.; Nakajima, T.; Honda, Y.; Kitao, O.; Nakai, H.; Vreven, T.; Throsse, K.; Foresman, J. B.; Fox, D. J. Gaussian09 Revision D.01. Gaussian, Inc.: Wallingford CT 2016.

(9) Zhao, Y.; Truhlar, D. G. The M06 Suite of Density Functionals for Main Group Thermochemistry, Thermochemical Kinetics, Noncovalent Interactions, Excited States, and Transition Elements: Two New Functionals and Systematic Testing of Four M06-Class Functionals and 12 Other Function. *Theor. Chem. Acc.* **2008**, *120* (1–3), 215–241. https://doi.org/10.1007/s00214-007-0310-x.

(10) Kozuch, S.; Shaik, S. How to Conceptualize Catalytic Cycles? The Energetic Span Model. *Acc. Chem. Res.* **2011**, *44* (2), 101–110. https://doi.org/10.1021/ar1000956.

(11) Glendening, E. D.; Reed, A. E.; Carpenter, J. E.; Weinhold, F. NBO Version 3.1.
